# Large-scale COVID-19 asymptomatic testing using lateral flow devices (LFDs) in reducing nosocomial infections and healthcare worker absenteeism: an observational study and cost-effectiveness analysis in NHS acute trusts in England

**DOI:** 10.1136/bmjph-2024-001618

**Published:** 2026-10-12

**Authors:** Siyu Chen, Rachel Hounsell, Liberty Cantrell, Lok Hei Tsui, Reshania Naidoo, Prabin Dahal, Richard Creswell, Sumali Bajaj, Jennifer A Flegg, Tom Fowler, Susan Hopkins, Ben Lambert, Merryn Voysey, Lisa White, Kasia Stepniewska, Rima Shretta, EY-Oxford Health Analytics Consortium, Ricardo Aguas

**Affiliations:** 1 Department of Health Data Science, University of Liverpool, Liverpool, UK; 2 Nuffield Department of Medicine, University of Oxford, Oxford, UK; 3 Department of Paediatrics, University of Oxford, Oxford, UK; 4 Department of Biology, University of Oxford, Oxford, UK; 5 Ernst & Young, London, UK; 6 Department of Computer Science, University of Oxford, Oxford, UK; 7 University of Melbourne School of Mathematics and Statistics, Parkville, Victoria, Australia; 8 UK Health Security Agency, London, UK; 9 University of Oxford, Oxford, UK; 10 Department of Statistics, University of Oxford, Oxford, UK

**Keywords:** COVID-19, Public Health, Community Health

## Abstract

**Introduction:**

Twice-weekly lateral flow device (LFD) testing for asymptomatic healthcare workers (HCWs) was introduced in the National Health Service (NHS) in England in November 2020. We retrospectively examine associations between LFD testing, nosocomial infections and HCW absenteeism at NHS acute trusts.

**Methods:**

We collected 16 million LFD testing results reported by NHS acute trusts from 1 October 2020 to 30 March 2022 and estimated the prevalence of nosocomial COVID-19 infections in NHS acute trusts using data from the International Severe Acute Respiratory and Emerging Infection Consortium (ISARIC). We used a mixed-effects linear model to examine the association between full-time equivalent (FTE) days lost due to COVID-19 and LFD test coverage. The relationship between weekly prevalence of nosocomial infections and LFD test coverage in the previous week was modelled using logistic regression weighted by the number of new COVID-19 cases reported in the ISARIC dataset for that week. We conducted an analysis of cost-effectiveness of testing for nosocomial infections.

**Results:**

Increased LFD test coverage was associated with decreases in FTE days lost due to COVID-19 from November 2020 to July 2021, with no association observed from August 2021 to March 2022. The proportion of nosocomial infections among new weekly cases in hospitalised patients was negatively associated with reported LFD testing levels.

**Conclusions:**

Higher LFD test coverage was associated with lower HCW absenteeism (November 2020–July 2021) and with a lower proportion of nosocomial infections among hospitalised patients. Given the retrospective, ecological design, these are associations rather than causal effects, and the cost-effectiveness estimates are valid only if the observed association reflects a causal effect of testing rather than confounding by co-occurring interventions or temporal trends in the pandemic. Under that assumption, LFD testing would have been a cost-effective measure for reducing nosocomial infections in NHS acute trusts.

**WHAT IS ALREADY KNOWN ON THIS TOPIC**

- Frontline healthcare workers (HCWs) had a significantly increased risk of SARS-CoV-2 infection.
- Infected HCWs could inadvertently transmit the virus to vulnerable patients, exacerbating the spread of the disease within the hospitals and increasing the burden of HCW absence.
- Regular testing for COVID-19 helped identify asymptomatic or presymptomatic cases among the healthcare workforce.

**WHAT THIS STUDY ADDS**

**HOW THIS STUDY MIGHT AFFECT RESEARCH, PRACTICE OR POLICY**

- In any future pandemic, HCWs testing interventions should incorporate collection of and/or timely access to relevant data, including HCW absenteeism, routine test results, community prevalence and hospitalisation and mortality data.
- The lessons learnt from this study could be used by relevant authorities to support the real-time assessment of any testing service and adjustment of the testing regimen.
- They could also be used to help develop more targeted and agile testing systems, which operationally would require the ability to turn mass testing off and on as an epidemic progressed.

## Introduction

 The COVID-19 pandemic led to a huge increase in the number of people requiring medical care including standard hospital treatment and critical care for acute infections,^1^ and for other conditions,^2^ especially in the beginning of 2020 when diagnoses for SARS-CoV-2 were sparse and vaccines and therapies for the diseases were underdeveloped. This had been putting immense pressure on healthcare workers (HCWs) and highlighted the importance of their resilience and well-being. In England, SARS-CoV-2 infection rates among HCWs were estimated to be at their highest between April and September 2020 and decreased progressively over time until October 2021.^3,4^ Infected HCWs could inadvertently transmit the virus to vulnerable patients, exacerbating the spread of the disease; during the early stages of the pandemic, nosocomial infections were estimated to be responsible for 20% of all identified COVID-19 cases in hospitalised patients.^5^ Regular testing for COVID-19 helped identify asymptomatic or presymptomatic cases among the healthcare workforce, allowing for prompt isolation and reducing the risk of transmission within healthcare settings using reverse transcription quantitative PCR^6^ and lateral flow devices (LFDs).^7,8^

Online supplemental table S1 provides a summary of the time periods of the different stages of the COVID-19 pandemic and their associated testing policies and epidemiological features in the National Health Service (NHS) in England; a more detailed summary of testing policy in English healthcare since January 2020 was reported elsewhere.^9,10^ LFD testing was introduced for routine asymptomatic testing of HCWs in England in November 2020; HCWs were requested to undertake twice-weekly LFD testing at home with a follow-up confirmatory PCR test in the case of a positive LFD result.^11^ This large-scale testing campaign was initially conducted by NHS Test and Trace, and later by the UK Health Security Agency (UKHSA), with the intention to support the NHS in its infection control risk reduction strategy and reduce staff absenteeism.^12^ HCWs with confirmed COVID-19 infections were initially required to self-isolate for 10 days,^13^ which was the same as the self-isolation guidance for the general population.^14^ Testing policy changed over time in response to the evolving epidemic and the vaccination campaign. The length of isolation was reduced from 10 to 7 days in December 2021 in the event of a negative LFD test result, with a further reduction to 5 days in January 2022.^15^ Confirmatory PCR tests following a positive LFD test result were no longer required from January 2022.^16^

In this study, we sought to determine the burden of absenteeism and nosocomial infections due to COVID-19 among HCWs in England between November 2020 and March 2022. We also explored correlations between these burdens and LFD test coverage in NHS acute trusts in England and the associated costs.

## Materials and methods

As part of a retrospective evaluation of England’s COVID-19 testing policy, we had access to asymptomatic testing data from NHS trusts (mainly acute trusts), which we later linked to testing cost, HCW absenteeism and estimates of nosocomial infections data. This enabled us to assess the association between level of testing reported in NHS acute trusts with HCW absenteeism and nosocomial infections. Furthermore, we compared the cost-effectiveness of counterfactual SARS-CoV-2 testing intensity levels in NHS acute trusts on resident quality-adjusted life-years (QALYs) gained. Our analysis period covered 1 October 2020 to 30 March 2022. More details on the data source and cleaning can be found in online supplemental appendices 1.1–1.10.

### Data acquisition and sources

#### LFD test volume and costs

LFD test volume data obtained via England’s Pillar 1 and Pillar 2 testing programmes^17^ from 1 October 2020 to 30 March 2022 from NHS acute trusts were provided by UKHSA, in the context of a retrospective evaluation of England’s COVID-19 testing policy.^9,18^ LFD test costs during the evaluation period were extracted from internal UKHSA documents and derived from the Cost Allocation Project conducted by UKHSA for the UK’s Office for National Statistics (ONS)^9^ (online supplemental figures S1–S5).

#### NHS trust headcount and full-time equivalent days lost

Monthly numbers of NHS Hospital and Community Health Service staff working in NHS trusts in England, presented as headcount and full-time equivalent (FTE) figures from 1 October 2020 to 30 March 2022, were retrieved from NHS Digital.^19^ FTE days lost due to COVID-19 for all staff, defined as the proportion of FTE days available that were lost due to COVID-19, were also downloaded from NHS Digital.

#### COVID-19 community prevalence in England

We calculated weekly estimates of COVID-19 community prevalence for all lower-tier local authorities (LTLA) within which each NHS acute trust was situated. These estimates were generated using a causal debiasing methodology,^20^ which used high-quality, randomised surveillance measures of swab-positive results provided via the REACT-1 survey^21^ to debias PCR testing data obtained through Pillar 2.

#### Variants of concern

Weekly proportions of SARS-CoV-2 lineages, including Alpha (B.1.1.7), Delta (B.1.617.2), Omicron (B.1.1.529) and ‘other’ lineages, by week and LTLA, were obtained from the Sanger Institute website.^22^

#### Healthcare worker vaccination

Data held in the NHS Electronic Staff Record (ESR) regarding the numbers of NHS acute trust HCWs who had been vaccinated for COVID-19 were retrieved from the NHS National Immunisation Management System.^23^ Cumulative vaccination data for HCWs were available from the end of August 2021, when high coverage for the first (85%) and second dose (75%) had already been achieved.

#### Average income deprivation scores

The English indices of deprivation at the LTLA level were extracted from the UK ONS on 1 November 2022. LFD test data, community COVID-19 prevalence and average income deprivation scores were provided per LTLA rather than per acute trust. Therefore, we used a function from the COVID-19.nhs.data package^24^ to calculate the number of tests per NHS acute trust in England, based on the proportional contribution of the number of tests conducted in LTLA regions within trusts (henceforth, we refer to this as the ‘mapping function’).

#### International Severe Acute Respiratory and emerging Infection Consortium database

Weekly numbers of new COVID-19 admissions or hospitalised patients newly diagnosed with COVID-19 from 136 NHS acute trusts were extracted through the International Severe Acute Respiratory and emerging Infection Consortium (ISARIC) dataset, which only contains patient medical records during hospitalisation. Among these cases, COVID-19 infections were classified as nosocomial infections if patients developed COVID-19 symptoms 7 days or more following their hospital admission. For a sensitivity analysis, we repeated these analyses using a 14-day cut-off. We included in our analysis all patients who were tested, had confirmed COVID-19 infection and were treated in an NHS acute trust in England. We excluded any re-admissions, records with conflicting dates, admissions outside of the study period (date of admission later than 31 March 2022 or date of discharge earlier than 1 October 2020) and COVID-19 cases outside of the study period (date of onset before 1 October 2020 or after 14 April 2022). For each week and for each acute trust in the ISARIC database (after exclusions), the total number of new COVID-19 infections, defined as new COVID-19 admissions or hospitalised patients with new COVID-19 symptoms, was calculated.

#### Hospitalisations and deaths data in England

Daily new hospitalised cases of COVID-19 in acute trusts in England were extracted from NHS Digital on 28 February 2023.^25^ Weekly total numbers of new cases per trust were calculated. COVID-19-related hospitalisations and deaths data in England between October 2020 and March 2022 were extracted from ONS.^26^ We used these data to calculate hospitalisation fatality ratios for our economic analysis. More details about the data accession processes are provided in the online supplemental appendices 1.1–1.4.

#### Patient and public involvement

As part of the COVID-19 testing initiative evaluation programme, addressing the issue of inequalities in COVID-19 testing engaged heavily with patient and the public. For example, we presented healthcare providers basic statistical findings to help encourage familiarity with research concepts and terminology. They described the barriers and motivations of implementing the testing policy, reporting the testing results and acting on a positive result in practice during the COVID-19 pandemic during stakeholder interviews. For example, stakeholders from healthcare organisations highlighted the “not all healthcare workers understood the testing guidance. A lack of understanding of the requirement to test was described as negatively affecting healthcare worker attitudes to the implementation of testing and motivations of implementing the testing policy, reporting the testing results and acting on a positive result in practice during the COVID-19 pandemic” (for more details, see the evaluation report^9^ and elsewhere^27,28^). We expanded the evaluation scope and refined the research findings to align them better with things that care providers cared about the most. Through these iterative processes, care providers made high-value contributions to both the design and the interpretation of the study, while we retained the rigours of the scientific work.

### Statistical and economic analysis

#### Analysis timeline

All analyses were conducted for four time periods to account for changes in healthcare testing policies, the vaccine rollout and the availability of data (online supplemental table S1). The four time periods were November 2020 to December 2020, January 2021 to July 2021, August 2021 to November 2021 and December 2021 to March 2022. Analyses were stratified by period, rather than pooled with period-by-coverage interactions, because the dominant variant, testing policy and covariate availability differed markedly across periods: the Alpha, Delta and Omicron prevalence variables are constant or close to zero outside the periods in which each variant circulated, and HCW vaccination data were available only from August 2021. Stratification therefore allowed each variant and time-varying covariate to be estimated within the period in which it varied; comparisons of the strength of associations across periods are descriptive rather than formal tests of effect modification.

#### Association between testing and HCW absenteeism

The primary outcome for our study was FTE days lost due to COVID-19, expressed as a proportion of total FTE days. This was calculated for each acute trust and month of the study, as only monthly absenteeism data were available. Because this proportion was right-skewed, it was modelled on the natural-log scale, with a small constant added before transformation to retain trust-months with no recorded COVID-related absence (for which the proportion was zero). The primary exposure variable was LFD test coverage, calculated as the number of tests reported per HCW, for each trust and month; this was also log-transformed and centred on each trust’s geometric mean, so that the model coefficients represent relative (multiplicative) changes (for further details, see online supplemental appendix 1.5). We fitted separate linear mixed models for each of the four time periods, each with a random intercept for trust and a fixed effect for month of evaluation, to examine the association between FTE days lost and LFD test coverage. The model was adjusted for community prevalence of COVID-19, average income deprivation score, relative prevalence of Alpha/Delta/Omicron variants (on a scale of 0–1) in England and acute trust LFD positivity rate (defined as the number of positive LFDs per headcount, calculated for each trust and month). Vaccination data were not included in the final model due to the sparsity of relevant data and low variability over time.

#### Association between testing and nosocomial infections

The secondary outcome of our investigation was the prevalence of nosocomial COVID-19 infections in NHS acute trusts, as reported to the ISARIC database (for further details, see online supplemental appendix 1.6). The relationship between weekly prevalence of nosocomial infections and LFD test coverage in the previous week was modelled using logistic regression with the weekly proportion of nosocomial infections as the outcome and the number of new COVID-19 cases as weights, equivalent to a binomial model with the case count as the denominator reported in the ISARIC dataset for that week. Week and trust were fitted as fixed effects (due to identifiability issues in fitting random effects). The model was adjusted for the total number of new COVID-19 cases reported per trust/week. It was also adjusted for possible confounders: community prevalence, average income deprivation score, relative prevalence of Alpha/Delta/Omicron variants (on a scale of 0–1) and LFD positivity rate per trust-month in the previous week (defined as the number of positive LFD tests for a given trust and month/headcount for a given trust and month×1000). As nosocomial infection was uncommon among hospitalised COVID-19 cases, the ORs from these models closely approximate risk ratios^29^; we therefore present these associations on the risk scale.

The model structures were compared by including cluster covariates (trust, month or week) and non-linear forms of covariates and interaction terms with transmission, and the final models were selected based on the Akaike information criterion and residuals. These models were then used to predict FTE days lost due to COVID-19 and the number of nosocomial infections in counterfactual scenarios where the LFD test intensity was set at 50%, 75%, 125% and 150% of the observed test level.

#### Economic analysis

The primary outputs of our economic analyses were the QALYs gained due to nosocomial infections averted within NHS acute trusts compared with various counterfactual LFD testing scenarios (50%, 75%, 125%, 150% and 200% of the actual testing volume). The counterfactual projections underpinning these economic outputs assume that the modelled associations reflect a causal effect of testing of the estimated magnitude, that there is no unmeasured confounding beyond the covariates adjusted for, that the functional form of the exposure-outcome relationship is correctly specified and that this relationship is stable across the range of counterfactual testing intensities considered (25%–200% of observed testing). These are strong assumptions that cannot be verified from observational data, and the economic estimates should be interpreted in that light. The evaluation was conducted from the healthcare provider’s perspective, as the UK government funded all direct costs of testing and treatment for COVID-19. The time horizon of the analysis (October 2020 to March 2022) was determined by the period of interest for the UKHSA-commissioned retrospective independent evaluation of COVID-19 testing in England.^9^ The secondary outputs of our economic analyses were the cost savings per nosocomial infection averted, per death averted and QALYs gained within NHS acute trusts under various counterfactual LFD testing scenarios. We also conducted a subanalysis for the two financial years (FY) in England covered by the study period, that is, FY21, comprising the 6-month period from October 2020 to March 2021; and FY22, comprising the full FY from April 2021 to March 2022.

We present here the incremental costs per nosocomial infection averted, incremental costs per death averted and incremental cost-effectiveness ratio (ICER) (defined as the incremental cost per QALY gained) due to testing. Given the short time horizon of the study, no discounting or adjustment for inflation was carried out. All costs and results were collected and are presented in pounds sterling (GBP) (for further details, see online supplemental appendix 1.7).

## Results

### HCW absenteeism

Monthly FTE days lost due to COVID-19 varied over time, by trust type and by staff group, ranging between 0% and 4% of the total corresponding FTE days available (figure 1A). Monthly FTE days lost due to COVID-19 accounted for 0%–60% of the total monthly FTE days lost for any reason (online supplemental figure S6). FTE days lost peaked at three time points: April 2020, January 2021 and January 2022 at 6.1%, 5.7% and 6.7%, respectively, with corresponding relative increases of 1.7-fold (90% CI 1.5 to 1.9), 1.2-fold (90% CI 1.2 to 1.3) and 1.4-fold (90% CI 1.3 to 1.5), respectively, compared with the median level from 2009 to 2019 (figure 1B).

**Figure 1:**
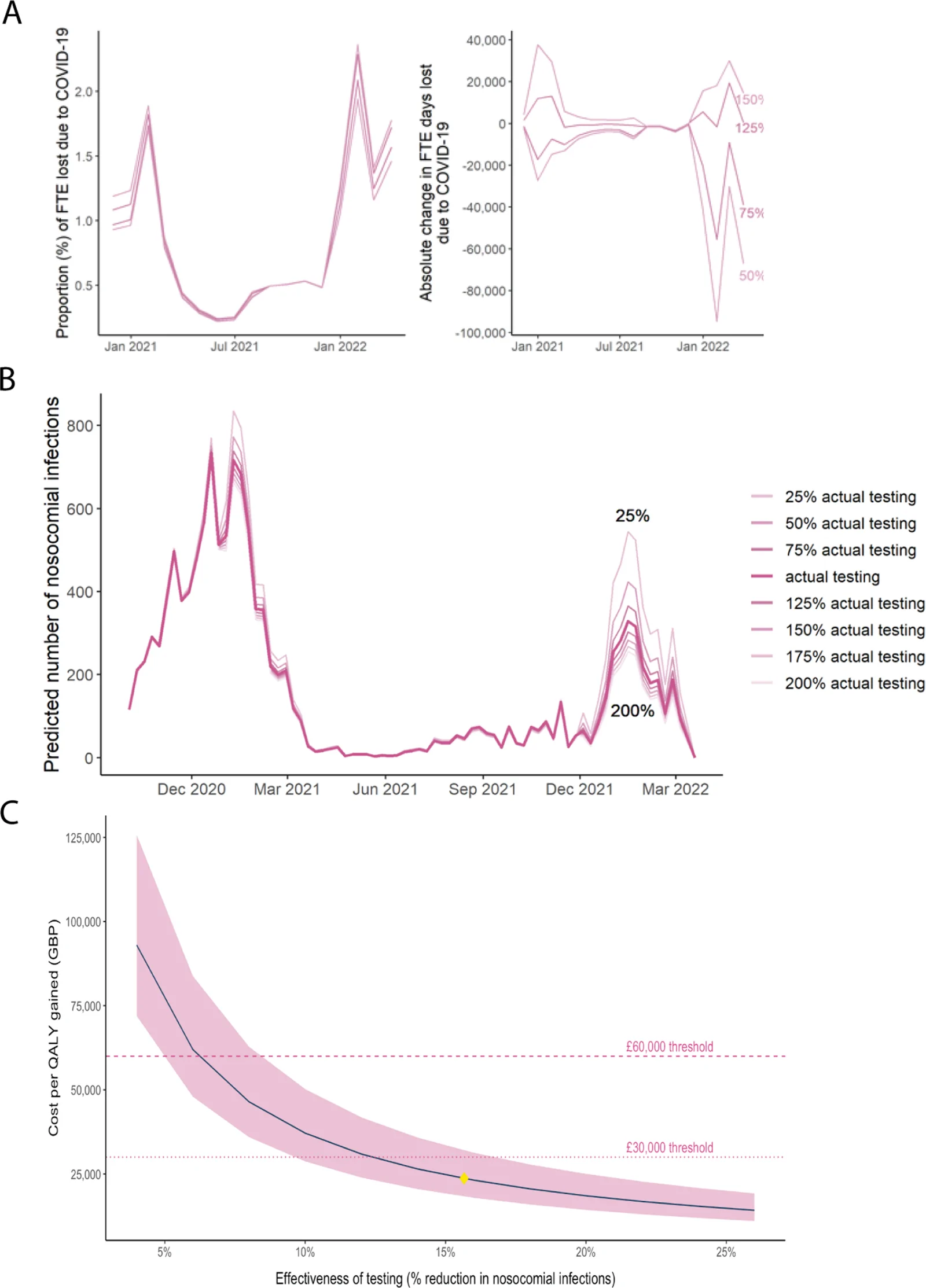
Monthly absenteeism and reported lateral flow device (LFD) tests in National Health Service (NHS) trusts in England from March 2020 to March 2022. (**A**) The percentage of monthly full-time equivalent (FTE) days lost due to COVID-19 by trust type and staff group over time. The percentage is calculated as the fraction between monthly FTE days lost due to COVID-19 and monthly FTE days available and then multiplied by 100%. The colours of lines represent different occupations of NHS staff. Four panels represent the four different types of NHS trusts. ACT, acute trust; AMT, ambulance trust; CMT, community trust; MHU, mental health trust. (**B**) Sickness absence rates in all staff groups at NHS acute trusts. The rates are defined as the fraction between number of days off sick and total workforce days contracted and then multiplied by 100%. The orange line represents the overall percentage of FTE days lost; the green line with the shaded area indicates the median and 90% range of sickness absence rates from 2009 to 2019. (**C**) Monthly number* of LFDs reported per healthcare worker (HCW) by trust type from March 2020 to March 2022*. *LFDs were reported in MHU trusts in England, with a median 1.0 (IQR 0–2.0) tests per HCW per week; 0.68 (0.081–1.29) in November–December 2020; 1.69 (1.37–2.45) in January–July 2021; 1.08 (1.03–1.28) in August 2021–November 2021 and 1.07 (0.85–1.65) in December 2021–March 2022. LFDs were reported in AMT trusts in England, with a median 0.58 (0–2.2) tests per HCW per week; 1.09 (0.24–1.93) in November–December 2020; 1.25 (0.90–2.50) in January–July 2021; 0.65 (0.53–0.68) in August 2021–November 2021 and 0.71 (0.55–1.23) in December 2021–March 2022. LFDs were reported in CMT trusts in England, with a median 2.1 (0–3.8) tests per HCW per week; 1.10 (0.13–2.07) in November–December 2020; 2.25 (2.05–3.09) in January–July 2021; 2.45 (2.22–2.96) in August 2021–November 2021 and 3.23 (2.85–4.71) in December 2021–March 2022.

NHS staff absences due to sickness (all causes) varied geographically, reflecting the sweep of the pandemic across England. In the East of England and the Northwest, absence rates increased more rapidly and to a higher peak than in other regions (online supplemental figure S7). Staff groups were also impacted differently by absences related to COVID-19, with doctors hit particularly hard in the first and third epidemic waves in England, in April 2020 and January 2022, respectively, when >40% of all doctors’ absences were due to COVID-19 (online supplemental figure S8). During the winter wave of January 2021, ambulance staff experienced the greatest number of FTE days lost due to COVID-19 (online supplemental figure S8). Variability between acute trusts was small and could possibly be explained by differences in staff composition (online supplemental table S2).

### Reported LFD test volumes, coverage and costs

Overall monthly volumes of reported LFDs followed similar trends across all healthcare settings except for community trusts and varied among the four time periods (figure 1C). A total of 16 352 554 LFDs were reported from October 2020 to March 2022, across the 136 acute trusts. A median of 0.83 (IQR 0.16–1.50) LFDs per HCW per week was reported between November and December 2020; 1.21 (IQR 0.87–2.17) between January and July 2021; 0.71 (IQR 0.62–0.79) between August and November 2021 and 0.77 (IQR 0.60–1.2) between December 2021 and March 2022. The level of reporting also varied among trusts across the four evaluation periods, with the second time period (January 2021 to July 2021) having the highest average coverage (online supplemental table S3). Only seven out of 136 trusts (5.0%) presented with less than one test per month for at least 1 month (online supplemental figure S9).

The total financial cost of the healthcare testing service for the full evaluation period was GBP 1.77 billion, representing 7.6% of the total testing expenditure in England. This cost did not include payments made to HCWs for isolating following a contact with a positive COVID-19 case. LFD costs comprised approximately 92.5% (GBP 1.64 billion) of the total costs of the HCW testing service. Approximately 55% of these costs were direct costs, 19% were indirect costs and the remainder were overhead costs. Overhead and indirect costs were marginally higher in FY21 than in FY22 due to the initial costs of setting up the testing service. The average unit cost of an LFD distributed under the healthcare testing service was GBP 11.68. Overhead costs were only attributed to Pillar 2 testing.

### Association between FTE days lost and test coverage

Associations between the proportion of FTE days lost due to COVID-19 and test coverage, as well as other covariates, are shown in online supplemental figures S10–S15. No apparent associations between test coverage and percentage FTE days lost due to COVID-19 were observed except during the time period January 2021 to July 2021 (online supplemental figure S10). The increase in LFD test coverage was associated with decreases in FTE days lost due to COVID-19 during the first two time periods (table 1, models 1 and 2). During the next two periods (table 1, models 3 and 4), no such association was observed. Higher community prevalence levels were associated with significant increases in FTE days lost due to COVID-19 in all periods except for the prevaccination period. Effect sizes ranged from 0.23% to 0.46% increases in FTE days lost for each 1% relative increase in the community prevalence of COVID-19. Similarly, LFD positivity rates in HCWs were positively associated with FTE days lost due to COVID-19. Average income deprivation scores were not associated with lost FTE days. The changes in testing levels (50%–150%) were estimated to result in modest changes in FTE days lost due to COVID-19 for all time periods (figure 2A).

**Figure 2:**
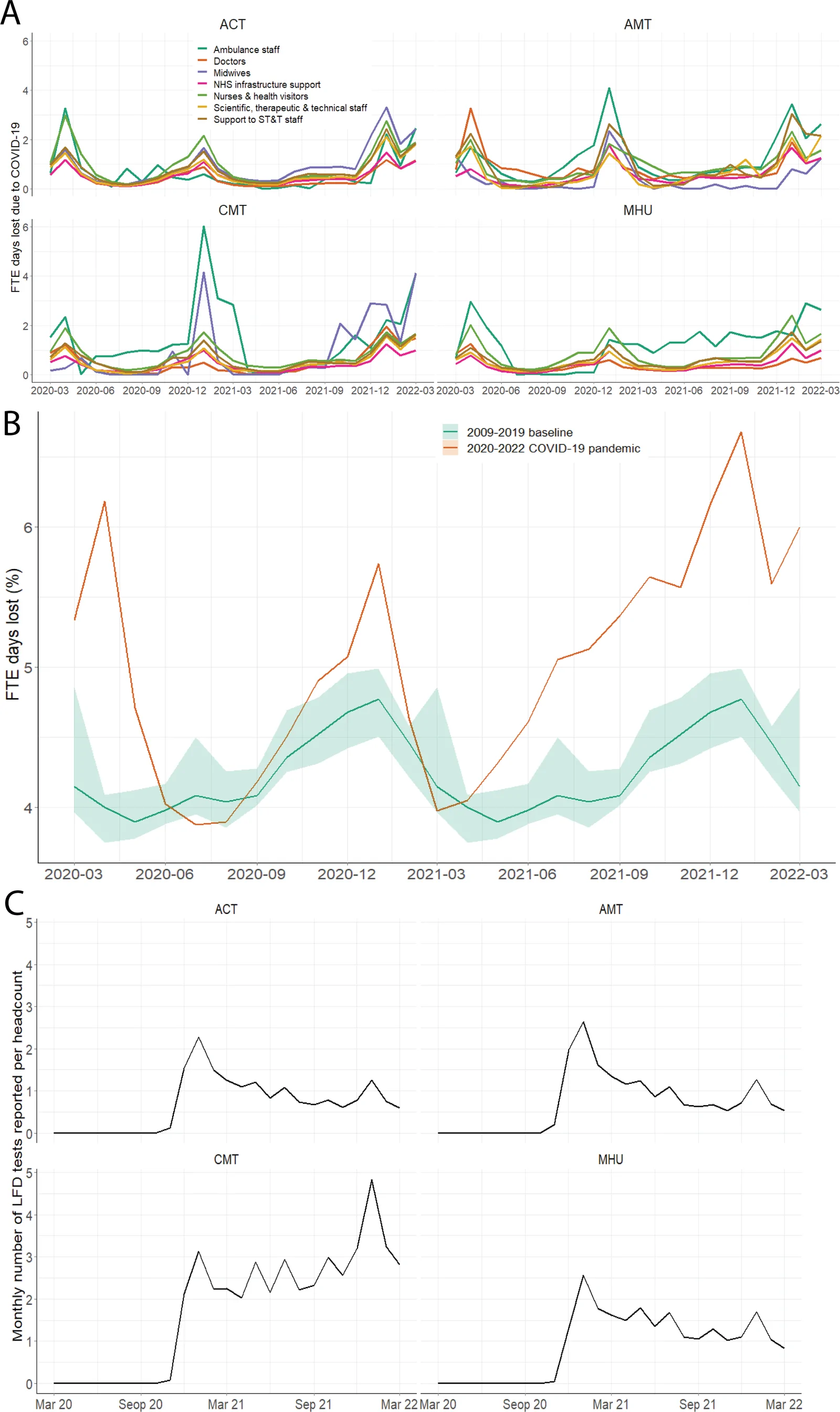
(**A**) Predicted proportion and absolute FTE days lost due to COVID-19 in all acute trusts. (**B**) Weekly number of nosocomial infections predicted for different testing scenarios, for 136 trusts included in the analysis. (**C**) Cost-effectiveness of weekly testing at different levels of testing effectiveness with respect to averting nosocomial COVID-19 infections in England. The shaded area indicates the cost per QALY gained at an upper value of 8.8 QALYs per death averted and a lower value of 4.98 QALYs per death averted. The line shows the analysis conducted at a value of 6.78 QALYs per death averted. The yellow point at 16.8% represents the statistically modelled level of testing effectiveness in reducing nosocomial infections.

**Table 1:** Estimated relative change (%, with 95% CI) in the proportion of the total available FTE days lost due to COVID-19 in the four time periods examined

	November 2020–December 2020 (prevaccination) (model 1)	January 2021–July 2021 (vaccine rollout) (model 2)	August 2021–November 2021 (Delta) (model 3)	December 2021–March 2022 (Omicron) (model 4)
Number of observations*	134	832	432	490
LFD coverage (per 1% increase)	−0.29 (−0.50 to −0.07)	−0.11 (−0.18 to −0.04)	−0.03 (−0.13 to 0.08)	−0.02 (−0.16 to 0.12)
Acute trust LFD positivity (per 1% increase)	0.28 (0.06 to 0.51)	0.19 (0.13 to 0.25)	0.07 (−0.01 to 0.15)	0.30 (0.21 to 0.39)
Local COVID-19 prevalence (per 1% increase)	0.07 (−0.54 to 0.68)	0.23 (0.12 to 0.35)	0.29 (0.14 to 0.44)	0.46 (0.24 to 0.68)
Alpha variant prevalence (per 0.1 increase)	8.44 (−1.88 to 19.84)	4.39 (−0.01 to 8.99)	N/A	N/A
Delta variant prevalence (per 0.1 increase)	N/A	1.92 (−2.96 to 7.04)	N/A	−0.30 (−11.71 to 12.58)
Omicron variant prevalence (per 0.1 increase)	N/A	N/A	N/A	8.11 (−5.19 to 23.28)
Average income deprivation score† per trust (per 0.01 unit increase)	1.51 (−3.72 to 7.03)	−2.47 (−5.48 to 0.64)	2.53 (−1.02 to 6.21)	0.10 (−4.12 to 4.51)

These estimates are from linear regression models as described in the text.

* Number of trust/months observations included in the model.

† Average income deprivation score: median (range) 0.123 (0.053–0.239) across trusts included in the analysis. A higher score means greater deprivation. 95% CIs are shown in brackets.

FTE, full-time equivalent ; LFD, lateral flow device; N/A, not available.

### Nosocomial infections in NHS acute trusts

In the ISARIC database, 136 NHS acute trusts were represented, with data available for a median of 54 (range 1–75) weeks. Overall, 3794 nosocomial infections after 7 days of admission (2049 after 14 days of admission (online supplemental table S4) were identified among 106 377 hospitalised patients with COVID-19 (online supplemental table S5). The median (range) number of new COVID-19 cases per week was 9 (1–531) with a median (range) of 0 (0–40) nosocomial infections identified using 7 days as the cut-off. Table 2 shows the distribution of trust-level parameters for hospitalised patients with COVID-19 with/without nosocomial infections.

**Table 2:** Summary statistics for measured covariates in hospitalised COVID-19 cases, by type of COVID-19 infection

Parameter	Nosocomial infection	Summary statistics				
		Minimum	p25*	Median	p75*	Maximum
November–December 2020						
LFD coverage (per 1000)	No	0	0	0.052	68.7	2121
	Yes	0	0	0	84.3	2121
Acute trust LFD positivity (per 1000)	No	0	0	0	1.37	64.4
	Yes	0	0	0	1.45	64.4
Local COVID-19 prevalence	No	0.001	0.008	0.011	0.016	0.031
	Yes	0.001	0.007	0.010	0.014	0.030
Alpha variant prevalence	No	0	0	0.008	0.215	1
	Yes	0	0	0.014	0.264	1
Average income deprivation score	No	0.053	0.115	0.141	0.167	0.240
	Yes	0.053	0.108	0.126	0.154	0.235
January–July 2021						
LFD coverage (per 1000)	No	0	176	352	523	1848
	Yes	0	221	417	618	1478
Acute trust LFD positivity (per 1000)	No	0	1.25	5.38	17.6	134
	Yes	0	2.00	7.43	22.9	134
Local COVID-19 prevalence	No	0.0001	0.007	0.010	0.015	0.034
	Yes	0.0001	0.006	0.010	0.015	0.029
Alpha variant prevalence	No	0	0.335	0.872	0.949	1
	Yes	0	0.699	0.898	0.956	1
Delta variant prevalence	No	0	0	0	0.323	1
	Yes	0	0	0	0	1
Omicron variant prevalence	No	0	0	0	0	0.012
	Yes	0	0	0	0	0.011
Average income deprivation score	No	0.053	0.110	0.137	0.167	0.239
	Yes	0.053	0.108	0.122	0.147	0.235
August–November 2021						
LFD coverage (per 1000)	No	0	58.8	127	225	1432
	Yes	0	75.3	126	220	1305
Acute trust LFD positivity (per 1000)	No	0	0.739	2.01	3.53	289
	Yes	0	0.858	2.22	3.58	14.6
Local COVID-19 prevalence	No	0.003	0.009	0.011	0.013	0.026
	Yes	0.003	0.010	0.011	0.013	0.021
Alpha variant prevalence	No	0	0	0	0	0.004
	Yes	0	0	0	0	0.0002
Delta variant prevalence	No	0.964	0.997	1	1	1
	Yes	0.975	0.998	1	1	1
Omicron variant prevalence	No	0	0	0	0	0.013
	Yes	0	0	0	0	0.008
Average income deprivation score	No	0.057	0.108	0.135	0.168	0.239
	Yes	0.057	0.108	0.141	0.184	0.235
December 2021–March 2022						
LFD coverage (per 1000)	No	0	61.9	146	276	2437
	Yes	0	85.1	158	276	1240
Acute trust LFD positivity (per 1000)	No	0	2.49	14.9	33.3	479
	Yes	0	5.13	24.1	41.9	144
Local COVID-19 prevalence	No	0.006	0.023	0.033	0.041	0.057
	Yes	0.008	0.028	0.035	0.039	0.051
Delta variant prevalence	No	0	0.001	0.015	0.199	1
	Yes	0	0.0002	0.006	0.038	1
Omicron variant prevalence	No	0	0.768	0.962	0.981	1
	Yes	0	0.936	0.976	0.985	1
Average income deprivation score	No	0.057	0.116	0.141	0.170	0.235
	Yes	0.066	0.122	0.141	0.166	0.235
						

LFD coverage, LFD positivity and prevalence data are per week.

* p25=25% centile; p75=75% centile.

LFD, lateral flow device.

### Association between nosocomial infections and test coverage

The proportion of nosocomial infections among new weekly cases in hospitalised patients (which included new admissions and cases diagnosed in hospital) was negatively associated with reported LFD testing levels (figure 2B). However, the strength of the association varied over time and was estimated to be highest during the Omicron period, with a doubling of the test coverage associated with 22% (95% CI 4% to 37%) fewer nosocomial infections (table 3). Our model predicted that the observed HCW testing/reporting was associated with 16.8% (95% CI 8.2% to 18.8%) fewer nosocomial infections than predicted under a counterfactual scenario of 25% of observed testing, under the assumptions stated in ‘Methods’ section (figure 2B, online supplemental figure S16 and online supplemental table S6). A 0.1 increase (a 10-percentage point increase) in the proportion of circulating Omicron, compared with other variants, was associated with approximately 1.35 times the risk of a hospitalised case being nosocomial (OR 1.35, 95% CI 1.07 to 1.70 presented in table 3 when changes in Omicron were compared with any other circulating variants). No association between nosocomial infections and LFD positivity rate in HCWs, or the population prevalence, was observed for any of the time periods. A negative association was observed between nosocomial infections and average income deprivation score, although this analysis was only conducted for the first two time periods, due to identifiability issues. Similar results were obtained in the sensitivity analysis when a nosocomial infection was defined as a patient who developed COVID-19 symptoms after 14 days since their admission (online supplemental table S7).

**Table 3:** Logistic regression model for the prevalence of nosocomial COVID-19 infections among all COVID-19 infections in hospitalised patients

	November 2020–December 2020 (prevaccination) (1)	January 2021–July 2021 (vaccine rollout) (2)	August 2021–November 2021 (Delta) (3)	December 2021–March 2022 (Omicron) (4)
Number of observations*	1314	2852	1489	829
LFD coverage (per 100% increase)	0.98 (0.96 to 0.99)	0.92 (0.89 to 0.95)	1.06 (0.89 to 1.26)	0.78 (0.63 to 0.96)
Acute trust LFD positivity (per 1/100 increase)	0.90 (0.82 to 1.00)	0.96 (0.89 to 1.04)	0.87 (0.53 to 1.42)	1.03 (0.97 to 1.09)
Local COVID-19 prevalence (per 1/100 increase)	0.90 (0.74 to 1.09)	0.76 (0.53 to 1.11)	0.84 (0.45 to 1.53)	1.02 (0.82 to 1.27)
Alpha variant prevalence (per 0.1 increase)	1.02 (0.96 to 1.08)	1.00 (0.92 to 1.08)	N/A	N/A
Delta variant prevalence (per 0.1 increase)	N/A	1.00 (0.80 to 1.24)	Delta variant prevalent	1.33 (0.67 to 2.66)
Omicron variant prevalence (per 0.1 increase)	N/A	N/A	N/A	1.72 (0.90 to 3.27)
Average income deprivation score† per acute trust (per 0.01-unit increase)	0.16 (0.07 to 0.33)	0.53 (0.27 to 1.07)	N/A	N/A

ORs (95% CI) are shown.

* Number of acute trust/week observations included in the model.

† Average income deprivation score, median (range) 0.130 (0.053–0.239) across trusts included in the analysis. A higher score means greater deprivation; 95% CIs are shown in brackets. The results are adjusted for trust and calendar week (as fixed effects) and the number of new COVID-19 cases identified at the trust that week.

LFD, lateral flow device; N/A, not applicable.

### Economic analysis of LFD testing on nosocomial infections averted

At a testing effectiveness of 8%–20%, between 17 500 and 43 800 nosocomial infections were averted during weekly testing of HCWs. The number of deaths averted ranged from 5500 to 13 800, with a saving per death averted of GBP 127 900–320 800. Between 38 100 and 95 100 QALYs were gained, translating to a value per QALY gained of GBP 18 500–46 400. A sensitivity analysis of the QALY values for deaths generated a saving of GBP 25 000–62 800 per QALY gained at a value of 4.98 QALYs per death averted and GBP 14 300–35 900 per QALY gained at a value of 8.8 QALYs per death averted at a testing effectiveness of 4%–26% (online supplemental tables S8 and S9).

At a daily testing effectiveness of 15%–30%, between 32 800 and 65 700 nosocomial infections were averted due to testing of HCWs. The number of deaths averted ranged from 10 300 to 20 700, with a cost per death averted of GBP 314 800–630 300. The cost per QALY gained was GBP 45 600–91 300. A sensitivity analysis of the QALY values per death averted found this would cost GBP 61 600–123 400 per QALY gained at a value of 4.98 QALYs per death averted and GBP 35 300–70 600 per QALY gained at a value of 8.8 QALYs per death averted (online supplemental tables S8 and S9).

The ICER (incremental costs per QALY gained) for various assumptions of testing effectiveness with respect to reducing nosocomial COVID-19 infections in acute trusts in England is illustrated in figure 2C. The range (shaded area) shown in the plot indicates the cost per QALY gained at an upper value of 8.8 QALYs per death averted and a lower value of 4.98 QALYs per death averted. The HCW testing service was estimated to be cost-effective at reducing nosocomial infections at values of effectiveness of 5.5% or more, at a willingness to pay threshold of GBP 70 000. Our statistical analysis estimated that HCW testing was associated with 16.8% (95% CI 8.2% to 18.8%) fewer nosocomial infections than predicted under a counterfactual scenario of 25% of actual levels. This suggests that for all values of effectiveness predicted in the 95% CI, the HCW testing service was cost-effective.

The yellow dot in figure 2C indicates that at an overall 16.8% decrease in nosocomial infections, the HCW testing service was cost-effective, at a cost of GBP 22 100 per QALY gained. This is well below the willingness to pay threshold of GBP 70 000 published by the UK government.^30^ The HCW testing service was also cost-effective at reducing nosocomial infections at values of effectiveness of 12.5% or more at the National Institute for Health and Care Excellence threshold of GBP 30 000.^31^ Compared with a hypothetical scenario of no testing, the HCW testing service contributed to an even greater reduction in nosocomial infections and hence was highly cost-effective.

## Discussion

There was substantial heterogeneity in FTE days lost by geographical locations of trusts, trust type and staff group, likely reflecting the heterogeneity of COVID-19 transmission and the risks associated with being an HCW.^32^ The estimated nosocomial infections in our study are around 1% higher than a previous estimate of nosocomial infections in England between June 2020 and March 2021^33^ but dramatically lower than reported from other studies that focused on the early days of the pandemic.^5,34–36^ The reported number of LFD tests from NHS acute trusts was likely to be an underestimate of the actual number of tests performed. Possible reasons for this discrepancy include biases in reporting, for example, some negative results may not have been reported as there was no infection prevention and control (IPC) action required for the individual HCW themselves or for their trust; furthermore, some individuals may not have reported positive results if there would have been an impact on their personal income or the income of their family due to them having to isolate^9,27,28^ (see online supplemental appendix 1.8 for more details). The underestimation of total volume of LFD tests and coverage of LFD per headcount might bias the correlation with HCW absenteeism and nosocomial infections, although the direction of bias (over or under) is unknown, and which might also be varying over epidemic progression. To reduce the consequence bias of LFD positivity, we adjusted the community prevalence of COVID-19 in the logistic regressions. Considering the heterogeneity of testing and reporting, we would be very cautious to make recommendation for future testing regime, for example, once a week vs twice a week.

Testing could potentially have two different effects on absenteeism, which work in opposite directions, where nosocomial infection is also an important mediator. Testing meant more cases would be identified and would need to isolate (both necessary isolation of asymptomatic cases and isolation of individuals who received false-positive results), leading to an increase in absenteeism. On the other hand, the isolation of individuals with true-positive results would reduce onward transmission and subsequent isolations, leading to a reduction in staff absenteeism. The balance of these two dynamics might change over time and at different comparator levels of testing. Our modelling results imply that increasing the volume of asymptomatic testing prior to August 2021 reduced absence rates among HCWs, but this impact was subsequently diminished by the high level of vaccination coverage. Similar observations have been reported elsewhere. For example, a hospital in the Brazilian city of Campinas experienced a significantly reduced level of hospital absenteeism due to COVID-19 from March 2020 to September 2020 by using PCR tests for screening and then requiring the isolation of asymptomatic HCWs who had tested positive, resulting in a reduction in transmission of COVID-19 to hospitalised patients.^37^ On the other hand, another modelling study showed that, compared with no testing, testing all HCWs would prevent most transmission, but at the expense of greatly increased staff absences and greater capacity for testing needed.^38^

Although the rollout of vaccination may have made ongoing testing appear to be marginally less cost-effective, continued testing should be considered a reasonable and overall cost-effective insurance strategy given the uncertainties around the long-term effectiveness of vaccines and the potential for future variants of concern (VOCs). This also raises the question of whether a standard health economics approach is appropriate for analyses such as this following the rollout of vaccination. In England, testing was continued based on the precautionary principle and because the potential impact of new variants was unknown. Traditional cost-effectiveness analyses do not take into account the willingness to pay for this type of assurance. We used a provider approach for our economic analysis. A societal approach that incorporates the economic cost of illness, including losses due to absenteeism, while not accounting for the assurance against uncertainty, is likely to result in testing being more cost-effective even after vaccination. At the same time, an analysis conducted for different time periods may have shown that the intervention was not cost-effective, especially with VOCs that were less severe or transmissible, for example, Omicron.

Higher community prevalence levels were associated with significant HCWs absenteeism even after adjusting for testing levels. This suggested that although testing could have a positive impact on mitigating outbreaks within healthcare settings and reducing HCWs’ absenteeism at a given level of community transmission rate, the overall community prevalence placed greater external pressure on outbreak management and HCW attendance. We might anticipate HCWs absence rates to be higher when there is greater community transmission, but if testing is particularly protective at that point, then testing would play a key role in outbreak mitigation within healthcare settings. The model estimates that increased LFD test coverage was associated with decreases in FTE days lost due to COVID-19 from November 2020 to July 2021, with no association observed from August 2021 to March 2022. This is perhaps due to heterogeneity in vaccination coverage or shifts in the proportions of COVID-19 strains.

The risk of developing nosocomial infections was estimated to vary for different VOCs. Another modelling study demonstrated that the risk of a highly transmissible (including presymptomatic transmission) variant could amplify nosocomial infections, for example, it was estimated a variant with 56% higher transmissibility would increase nosocomial transmissions by 303%.^39^

Our study has some limitations. Data were not available that would have enabled us to quantify the impact of patients with undetected COVID-19 being released into the community and potentially infecting others through secondary transmission. We could also not account for the effects of long COVID in our economic analysis, as these effects could not be quantified. Furthermore, the nosocomial infections estimated using the ISARIC dataset are likely to be biased by the inclusion criteria, in that only individuals who consented to participate in the study were included. More broadly, exposure was measured ecologically, at the trust and lower-tier local authority level rather than for individual HCWs, so the estimated associations are susceptible to ecological bias and residual confounding and should not be interpreted as individual-level causal effects. We were not able to link positive LFD results with any following (if any) PCR confirmatory testing results and thus were not able to assess the implementation of PCR confirmatory testing in practice and its associated impact on HCW absenteeism. A false-positive testing result from LFD corresponding to the specificity of LFD tests without PCR likely results in wasted units of HCW FTE. Due to the sparsity of vaccination data in our study, we were unable to assess the impact of vaccination on the correlation between testing and nosocomial infections and HCW absenteeism, although preliminary signals in 2021 show that high levels of vaccination coverage might dilute the effect of testing on reducing absenteeism. According to NHS Absences due to COVID-19 interactive report,^40^ the HCW FTE source data is “the Electronic Staff Record (ESR) Data Warehouse” and “FTE days lost due to COVID-19 are identified as records with a Related Reason category in ESR of ‘Coronavirus (COVID)’”. However, there is no publicly available exact definition of how cases were due to COVID-19. For the definition of nosocomial infections, the estimates could potentially be biased since patient might still acquire hospital-related infections already but tested positive only after discharge.

Our study has implications for the development of testing policies in the healthcare setting for future pandemics, particularly for respiratory pathogens similar to COVID-19. The negative association between nosocomial infections and average income deprivation scores suggests a target testing strategy for future epidemics and pandemics. There are many potential reasons for these negative correlations; for example, regions with higher deprivation scores tend to have more people with underlying health conditions,^41^ such as diabetes and chronic lung disease, which may increase nosocomial infections. In terms of healthcare quality, deprived regions tend to have less stringent hospital infection control, staff shortages, overcrowding and even environmental hygiene deficiencies,^42^ which are also associated with a higher likelihood of nosocomial infections. The finding highlights the importance and necessity of developing more targeted and agile testing systems, which operationally would require the ability to turn mass testing off and on as an epidemic progresses and was described as the primary intention of the retrospective pan-evaluation of the mass testing conducted during the COVID-19 pandemic.^43^ The rollout of HCW testing interventions through pilot studies, with collection of and/or timely access to data related to suitable endpoints (including HCW absenteeism, routine test results, community prevalence and hospitalisation and mortality data), could be used by relevant authorities to support the real-time assessment of any testing service and adjustment of testing interventions.

## Supplementary material

10.1136/bmjph-2024-001618 online supplemental file 1

## Data Availability

Data are available on reasonable request. Data may be obtained from a third party and are not publicly available.
