# Supplementary material for: Large-scale COVID-19 asymptomatic testing using lateral flow devices (LFDs) in reducing nosocomial infections and healthcare worker absenteeism: an observational study and cost-effectiveness analysis in NHS acute trusts in England
Source: BMJ Public Health. 2026 Oct 12;4(4):e001618. doi: 10.1136/bmjph-2024-001618 (PMC13662036; doi:10.1136/bmjph-2024-001618)
Supplement: Supplementary file 1 [file bmjph-4-4-s001.docx]

# Supporting Information

**Title:** Large-scale COVID-19 asymptomatic testing using lateral flow devices (LFDs) in reducing nosocomial infections and healthcare worker absenteeism: an observational study and cost-effectiveness analysis in NHS acute trusts in England

**Authors:** Siyu Chen^1,2*†^, Rachel Hounsell^1†^, Liberty Cantrell^3†^, Lok Hei Tsui^4†^, Reshania Naidoo^1,5†^, Prabin Dahal^1,6^, Richard Creswell^7^, Sumali Bajaj^4^, Jennifer A. Flegg^9^, Tom Fowler^10,11^, Susan Hopkins^10,12^, Ben Lambert^8^, Merryn Voysey^3^, Lisa J. White^4^, EY-Oxford Health Analytics Consortium^**^, Kasia Stepniewska^1,6^, Rima Shretta^1^

^1^Nuffield Department of Medicine, University of Oxford, Oxford, UK

^2^Department of Health Data Science, University of Liverpool, UK

^3^Department of Paediatrics, University of Oxford, Oxford, UK

^4^Department of Biology, University of Oxford, Oxford, UK

^5^Ernst & Young LLP London, UK

^6^Centre for Tropical Medicine and Global Health, University of Oxford, Oxford, UK

^7^Department of Computer Science, University of Oxford, Oxford, UK

^8^Department of Statistics, University of Oxford, Oxford, UK

^9^School of Mathematics and Statistics, University of Melbourne, Melbourne, Australia

^10^UK Health Security Agency, London, UK

^11^William Harvey Research Institute, Queen Mary University of London, London, UK

^12^NIHR Health Protection Research Unit in Healthcare associated infections and Antimicrobial Resistance, University of Oxford, Oxford, UK

^†^ These authors contributed equally.

*Corresponding author: Dr. Siyu Chen [stat.siyu@gmail.com](mailto:stat.siyu@gmail.com)

^**^EY-Oxford Health Analytics Consortium membership list

Ricardo Aguas, Ma’ayan Amswych, Billie Andersen-Waine, Sumali Bajaj, Kweku Bimpong, Adam Bodley, Liberty Cantrell, Siyu Chen, Richard Creswell, Prabin Dahal, Sophie Dickinson, Sabine Dittrich, Tracy Evans, Angus Ferguson-Lewis, Caroline Franco, Bo Gao, Rachel Hounsell, Muhammad Kasim, Claire Keene, Ben Lambert, Umar Mahmood, Melinda Mills, Ainura Moldokmatova, Sassy Molyneux, Reshania Naidoo, Randolph Ngwafor Anye, Jared Norman, Wirichada Pan-Ngum, Sarah Pinto-Duschinsky, Sunil Pokharel, Anastasiia Polner, Katarzyna Przybylska, Emily Rowe, Sompob Saralamba, Rima Shretta, Sheetal Silal, Kasia Stepniewska, Joseph Tsui, Merryn Voysey, Marta Wanat, Lisa J. White, Gulsen Yenidogan

Contents

[Supporting Information	1](#_Toc201056333)

[S1 Appendix – Methods	4](#_Toc201056334)

[1.1 Testing data	4](#_Toc201056335)

[1.2 Community prevalence of COVID-19 in England	4](#_Toc201056336)

[1.3. LFD costs	4](#_Toc201056337)

[1.4 Variable definitions	5](#_Toc201056338)

[1.5 Modelling the association between HCW absenteeism and LFD testing	6](#_Toc201056339)

[1.6 Modelling the association between nosocomial infections and LFD testing	7](#_Toc201056340)

[1.7 Cost-effectiveness analysis for nosocomial infections	8](#_Toc201056341)

[1.8 LFD testing contextual information	8](#_Toc201056342)

[1.9 Reported LFDs tests	9](#_Toc201056343)

[1.10 The positivity rate of LFD tests followed prevalence estimates	13](#_Toc201056344)

[S2 Appendix – supplementary figures and tables	14](#_Toc201056345)

[Fig S6. Percentage of monthly FTE days lost due to COVID-19 by trust type and staff group over time.	14](#_Toc201056346)

[Fig S7. Rates of NHS staff absences due to sickness (all causes) by region, March 2020 to March 2022.	15](#_Toc201056347)

[Fig S8. Ratios of monthly FTE days lost due to COVID-19 by staff group.	15](#_Toc201056348)

[Fig S9. Histogram of the number of LFD tests per healthcare worker by trust-month.	16](#_Toc201056349)

[Fig S10. Association between the proportion of FTE days lost due to COVID-19 and LFD testing coverage.	16](#_Toc201056350)

[Fig S11. Association between the proportion of FTE days lost due to COVID-19 and local COVID-19 prevalence.	17](#_Toc201056351)

[Fig S12. Association between the proportion of FTE days lost due to COVID-19 and trust LFD positivity.	17](#_Toc201056352)

[Fig S13. Association between the proportion of FTE days lost due to COVID-19 (in logarithmic 2 scale) and trust average income deprivation score.	18](#_Toc201056353)

[Fig S14. Association between the proportion of FTE days lost due to COVID-19 (in logarithmic 2 scale) and the prevalence of COVID-19 variants.	18](#_Toc201056354)

[Fig S15. Association between the prevalence of COVID-19 and LFD test coverage (in logarithmic 2 scale) for each of the four study periods.	19](#_Toc201056355)

[Fig S16. Lower and upper limits of the 95% confidence intervals (CIs) for the predicted weekly number of nosocomial infections for various testing scenarios**.**	19](#_Toc201056356)

[Table S1. Summary of time periods during the COVID-19 pandemic and their associated testing policies and epidemiological features.	19](#_Toc201056357)

[Table S2. Number of healthcare workers stratified by group across the entire evaluation period (1 October 2020 to 30 March 2022), in all NHS acute trusts.	21](#_Toc201056358)

[Table S3. Number of LFD tests per patient-facing staff member for the four study periods in all NHS acute trusts.	33](#_Toc201056359)

[Table S4. Summary of the numbers of nosocomial infections in 136 NHS trusts during the entire evaluation period, obtained from the ISARIC data.	45](#_Toc201056360)

[Table S5. Summary of hospitalised COVID-19 cases per week and per trust over the four evaluation periods.	50](#_Toc201056361)

[Table S6. Predicted total number of nosocomial infections in the acute trusts studied.	60](#_Toc201056362)

[Table S7. Sensitivity analysis.	60](#_Toc201056363)

[Table S8. Data inputs and assumptions for the testing service in healthcare	61](#_Toc201056364)

[Table S9. Summary of the cost effectiveness of weekly and daily testing with respect to nosocomial infections averted during the evaluation period (October 2020 to March 2022).	62](#_Toc201056365)

[Supporting information – references	62](#_Toc201056366)

# S1 Appendix – Methods

## 1.1 Testing data

We obtained separate data files for LFD and RT-PCR testing data conducted within Pillar 2 and one file for testing data conducted within Pillar 1 (for details on Pillars 1 and 2, see [1]). Pillar 1 data were filtered such that ‘RequestingOrganisationType’ = ‘HEALTHCARE WORKER TESTING’ and then aggregated by month and NHS trust name. Pillar 1 only provided testing data from acute trusts. Pillar 2 PCR and LFD data were both filtered such that ‘Channel’ = ‘Secondary Care’ and ‘OrganisationRole’ = ‘staff’. We aggregated testing data from Pillar 2 by month and lower-tier local authority (LTLA).

In the mixed-effects model, where we assessed the association between testing volume and NHS healthcare worker absenteeism, we focused solely on acute trusts by adding another filter criterion for Pillar 2 data, (‘OrganisationType’ = ‘ACT’). This was because only these trusts had LFD testing data available at the trust level from both Pillar 1 and Pillar 2. As Pillar 2 data were provided per LTLA rather than per trust, a function from the covid19.nhs.data package [2] was used to calculate the number of tests per trust, based on the proportional contribution of the number of tests conducted in LTLA regions within trusts (hereafter we refer to this as the ‘mapping function’). This mapping only maps to acute trusts.

## 1.2 Community prevalence of COVID-19 in England

To determine COVID-19 community prevalence, we used data from REACT, a randomised surveillance study conducted in the UK [3]. The REACT data consisted of numbers of tests conducted by week within each LTLA, along with the results of these tests. In week-LTLAs with fewer than five observations, the data were censored if there were any positive results to ensure the data were not identifiable. As the numbers of PCR tests conducted through the REACT programme in each LTLA in a given week were relatively low, this limited the direct usefulness of these data in our statistical models. Instead, we used the methods described by Nicholson and colleagues [4] to obtain estimates of greater precision of weekly LTLA-level COVID-19 prevalence by leveraging the Pillar 2 PCR testing data, which involved much greater testing volumes.

Specifically, this method uses prevalence data collected within larger-scale, ‘coarse’ regions to estimate the biases in mass testing data collected within the same regions. The models fitted to these coarse regions are then used to estimate prevalence at finer scales using only the mass testing data. By so doing, this method assumes that the biases in test-seeking behaviour inherent within the coarse regions are the same as in the fine-scale ones.

Here, we followed Nicholson and colleagues’ [4] approach by choosing the 9 Public Health England (PHE) regions of England to be the coarse regions and the 321 LTLAs in England to be the fine-scale regions. We fit the model to the PCR testing data and REACT data from 25 July 2020 to 2 April 2022. The measure of COVID-19 prevalence for each acute care home used within our models corresponded to the posterior mean prevalence estimated by the above method, corresponding to the LTLA encompassing each NHS trust. We also published relevant results elsewhere [5-6].

## 1.3. LFD costs

LFD testing data for the healthcare setting were provided by the UK Health Security Agency (UKHSA). We used data from the Cost Allocation Project (an unpublished internal project) conducted by UKHSA for the ONS as a starting point for our analysis. Testing cost data were primarily sourced from UKHSA general ledgers. Where funds were held by another department, such as the Department of Health and Social Care (DHSC), these had either been transferred or were added as an adjustment to the values at the point of analysis. We apportioned all costs to the various testing services (e.g. adult social care, healthcare workers) for each technology based on the applicable service volumes.

Test volume data (purchased and distributed) were provided by UKHSA. All costs and volumes used in the evaluation were for England only. We removed line items for the devolved administrations prior to conducting our analysis. The devolved administrations accounted for approximately 12% of test volumes distributed for financial year (FY)21 and 16% for FY22. We calculated unit costs for the full evaluation period (1 October 2020 to 30 March 2022), which captured the purchase price of tests, as well as all other direct, indirect and overhead costs associated with the testing programme. These included the logistics, human resources and other costs to deliver a test to the point of care. Unit costs excluded support payment costs and laboratory set-up costs. Unit costs decreased over time, thus the average costs differ by financial year.

## 1.4 Variable definitions

**Full-time equivalent (FTE) days lost.** The outcome variable, $y_{i}(t)$, was defined as the proportion of total FTE days available that were lost due to COVID-19 in the $i-th$ trust at the$t-th$ month. We only included monthly data from trusts whose FTE days available and percentage of FTE days lost due to COVID-19 were larger than 0 and the number of LFD tests per headcount was larger than 1 in that month. Due to the skewed distribution, this outcome was modelled following a log-transformation, $log(y_{i}\left( t \right))$:

$${log(y}_{i}\left( t \right))=log\left( \frac{FTE days lost due to covid in i-th trust at the t-th month}{total FTE days available in i-th trust at the t-th month} \right)$$

Equation (1)

In this study, $log$ refers to the natural logarithm.

The primary exposure variable of interest was **LFD test coverage**, $cov_{i}(t)$, i.e. the number of tests reported per healthcare worker in the $i-th$ trust in the$t-th$ month. For the analysis, we took the log-transformed due to having a skewed distribution and centred on the geometric mean within each trust as one of the covariates, $x_{1i}(t)$, in the mixed-effects linear model:

$$cov_{i}(t)=\frac{number of LFT tests at i-th trust at the t-th month}{number of headcount at i-th trust at the t-th month}$$

Equation (2)

$x_{1i}(t)$ *=*$log(cov_{i}(t)-mean(log\left( cov_{i}(t) \right) )$

Equation (3)

Separate linear regression models, with random effects for trust and fixed effects for the calendar month of the evaluation, were fitted for the four time periods described as Table 1 in the main text, at the monthly level.

**Trust LFD positivity per healthcare worker,** $x_{2i}(t)$. COVID-19 prevalence among healthcare workers in each trust for each month was estimated from LFD positivity rates and expressed per 1000 healthcare workers: number of LFD-positive tests in the $i-th$ trust at the$t-th$ month divided by headcount in the $i-th$ trust at the$t-th$ month × 1000:

$$x_{2i}\left( t \right)=\frac{number of reported positive LFD tests at i-th trust at the t-th month}{number of headcount at i-th trust at the t-th month}$$

Equation (4)

**Average income deprivation score** in the $i-th$ trust, $x_{3i}$. Average income deprivation scores were extracted from a dataset downloaded from the UK Office for National Statistics (ONS) [7]. This was the only deprivation score available at the LTLA level.

**Community COVID-19 prevalence** in the $i-th$ trust at the$t-th$ month, $x_{4i}\left( t \right)$. This was estimated at the LTLA- and week-level using the method described above.

**Relative prevalence of the Alpha/Delta/Omicron variants** $I_{\alpha}\left( t \right), I_{\delta}(t)$ and $I_{o}(t)$.

$I_{\alpha}\left( t \right), I_{\delta}(t)$ and $I_{o}(t)$ are dummy variables indicating whether the Alpha, Delta or Omicron variants were dominant.

England community COVID-19 prevalence, average income deprivation score and Alpha/Delta/Omicron variant relative prevalence were estimated at the acute trust level from LTLA estimates using the mapping function.

**Trust LFD coverage per healthcare worker per week**, $x_{5i}(t)$ is defined as the number of reported LFD tests in the $i-th$ trust at the$t-th$ week divided by the headcount in the $i-th$ trust at the$t-th$ week:

$$x_{5i}\left( t \right)=\frac{number of reported LFD tests at i-th trust at the t-th week}{number of headcount at i-th trust at the t-th week}$$

Equation (5)

**Vaccination data** for healthcare workers were only available from the end of August 2021, when high coverage for the first (85%) and second dose (75%) had already been achieved. Consequently, as we found no association between the number of vaccination doses and FTE days lost (in the adjusted model), and there were gaps in the data for some trusts, vaccination data were not included in the final model.

**The COVID-19 related mortality** was extracted from Office for national Statistics Deaths registered weekly in England and Wales, provisional Statistical bulletins [8]. According to ONS, mortality statistics in England and Wales are based on the details collected when deaths are certified and registered. Coronavirus (COVID-19) deaths are those deaths registered in England and Wales in the stated week where COVID-19 was mentioned on the death certificate. A doctor can certify the involvement of COVID-19 based on symptoms and clinical findings – a positive test result is not required [9].

**The COVID-19 overall hospitalisation data** were extracted from ONS Coronavirus (COVID-19) latest insights: Hospitals [10]. These data were collected by the UK Health Security Agency and available on the GOV.UK coronavirus dashboard [11] and national flu and COVID-19 surveillance reports [12]. This data come from the Severe Acute Respiratory Infection (SARI) Watch surveillance system, which monitors the number of patients with confirmed flu and COVID-19 admitted to hospital and critical care units (ICU and HDU) in England.

## 1.5 Modelling the association between HCW absenteeism and LFD testing

The mixed-effects linear model is defined as

$z_{i}\left( t \right)=x_{1i}\left( t \right)+{x_{2i}\left( t \right)+x_{3i}+x_{4i}\left( t \right)+I}_{\alpha}\left( t \right)+I_{\delta}\left( t \right)+I_{o}\left( t \right)+\left( 1|i \right)+\epsilon_{i}$

Equation (6)

Here, $(1|i)$ denotes the random effect from trusts, and $\epsilon_{i}$ denotes the regression residual following standard normal distribution.

The model with log-transformed LFD positivity by trust and community COVID-19 prevalence was selected as the most appropriate, based on Akaike information criterion (AIC) values and residuals.

## 1.6 Modelling the association between nosocomial infections and LFD testing

The logistic regression model is defined as

$$log\frac{p_{i}(t)}{(1-p_{i}(t))}=x_{5i}\left( t \right)+{x_{2i}\left( t \right)+x_{3i}+x_{4i}\left( t \right)+I}_{\alpha}\left( t \right)+I_{\delta}\left( t \right)+I_{o}\left( t \right)$$

Equation (7)

Here, $p_{i}(t)$ denotes the proportion of nosocomial infections at time $t$ among all hospitalised infections at trust $i$.

The logistic regression estimates represent odds ratios (OR) for a hospitalised COVID-19 infection being nosocomial. However, as there was a low prevalence of nosocomial infections among hospitalised infections, the OR is a good estimate of the risk ratio (RR) [13], and the narrative of the effects is presented in term of the risks (not the odds).

The results of the statistical models were used to project numbers of nosocomial infections under counterfactual testing scenarios of 50%, 75%, 125% and 150% LFD testing levels compared with the observed scenario. In any of the counterfactual scenarios we considered, any changes to the number of nosocomial infections were assumed to result in an equivalent change in the total number of new infections, i.e. the number of new COVID-19 admissions remained the same.

In the factual scenario, $p$ is the proportion of nosocomial infections among $N$new hospital cases, then

$$N_{noso}=p N$$

Equation (8)

is the corresponding number of nosocomial infections.

In a counterfactual scenario, $p_{new}$ is the model-estimated proportion of nosocomial infections, then

$$p_{new} =\frac{N_{noso}+x}{N+x}$$

Equation (9)

where $x$is the change in the nosocomial infections. Substituting the Equation (8) into Equation (9), we obtain:

$\frac{\left( pN +x \right)}{N+x}= p_{new}$

Equation (10)

then,

$$\frac{x=(p_{new}N-pN)}{(1-p_{new})}$$

Equation (11)

Therefore, the number of nosocomial infections under the new scenario $N_{noso}^{new}$ is calculated as:

$$N_{noso}^{new}=p N+x=\frac{p_{new} N \left( 1-p \right)}{\left( 1-p_{new} \right)}$$

Equation (12)

## 1.7 Cost-effectiveness analysis for nosocomial infections

Estimates of nosocomial infections averted in counterfactual scenarios of 50%, 75%, 125% and 150% LFD testing levels compared with the observed scenario were extracted from statistical models. The costs of these reduced and increased test volumes were adjusted proportionally. The overhead and indirect costs remained the same regardless of test volume. Only the direct costs and direct overhead costs were proportionally adjusted. A further sensitivity analysis was developed, assuming reductions in nosocomial infections of 4–26% due to weekly testing and 15–33% due to daily testing. Hospitalisation fatality ratios (HFRs) were calculated using ONS COVID-19 hospitalisation death data [14].

Probabilities and costs of requiring hospital treatment or intensive care unit (ICU) treatment following a COVID-19 infection were extracted from the literature [15] and are summarised in Table S7. Multiplying estimates of nosocomial infections averted by the probabilities of requiring hospital treatment, requiring ICU treatment, and HFRs, we calculated the number of hospital admissions averted, ICU admissions averted, and COVID-19-related deaths averted, respectively. QALYs for symptomatic COVID-19 infections and hospitalisations and deaths due to COVID-19 were also obtained from the literature [15]. We then used these values to calculate QALYs gained due to infections, hospitalisations and deaths averted.

Unit testing costs were calculated for the full evaluation period and captured the purchase price of tests, as well as all other direct, indirect and overhead costs associated with the programme, including the logistics, human resources and other costs to deliver tests to the point of care.

## 1.8 LFD testing contextual information

The healthcare workers testing service in England initially involved regular asymptomatic testing of patient facing staff of NHS trusts in November 2020, followed by primary care and independent healthcare providers shortly after. The healthcare workers testing service in England started with the rollout of the regular asymptomatic testing service for patient-facing staff of NHS trusts, which began in November 2020 [16], followed by primary care in January 2021 and independent healthcare providers in April 2021. Various technologies were explored as part of the effort to increase testing capacity, including LAMP (loop-mediated isothermal amplification) testing of saliva samples. The rollout of asymptomatic healthcare worker testing followed a pilot study involving 1200 staff members from five NHS trusts, which demonstrated that regular LAMP testing of saliva samples from asymptomatic staff was both feasible and acceptable [16]. Asymptomatic testing in healthcare settings was implemented in secondary care, primary care, the independent sector, maternity wards, emergency departments and end-of-life services [17]. Interviews with healthcare testing service stakeholders showed that once the first satisfactory results about the sensitivity and specificity of LFDs were available, LFDs became the main tests for the asymptomatic testing service. They offered a rapid result and enabled home testing. LAMP tests were only used in a limited number of trusts.

LFD tests were initially distributed to the NHS based on an on-demand ‘push’ model to trusts. With this model, trusts received a stock of tests and were responsible for test distribution among sites, storage, recording of orders and reporting of aggregated test results shared by their staff. This was later followed by a ‘pull’ model, driven by individual staff orders via a gov.uk website, with tests delivered directly to the homes of staff from July 2021. Staff were required to input the name of the trust where they worked to ensure the tests were registered as belonging to an NHS staff member. In parallel with the deployment of LFDs, LAMP tests were used on saliva samples. LAMP tests were recommended for staff members who were unable to perform an LFD test, for whatever reason, when they were available to the local NHS organisation.

Organisations and regions received management information from NHS Test and Trace to help them to understand what proportion of their staff had ordered and reported tests. However, trusts continued to receive a supply of LFDs for patient use as per existing use cases (emergency departments, maternity and neonatal departments, and end-of-life-care visitors) [18].

The general policies relating to asymptomatic testing and isolation were established by various teams within UKHSA, the Department of Health and Social Care (DHSC) and NHSE and were made available on the gov.uk website. Every health organisation was then responsible for clear communication of these policies to their staff members through their local organisations.

There was a single national standard operating procedure (SOP) and frequently asked questions (FAQs) document for twice-weekly asymptomatic testing with LFDs in NHS trusts, an SOP and FAQs for primary care, and an SOP for independent healthcare providers. In many instances, NHS staff were required to follow the general guidance for self-isolation. Testing and self-isolation guidance for close contacts of COVID-19 cases varied during the period covered by this evaluation.

**Site set-up.** For asymptomatic testing, where LAMP tests were piloted, healthcare workers were advised to conduct Direct LAMP tests on-site or LFD tests (if available and required) at home, ideally before attending work. From November 2020 until the updated SOP in July/August 2021, NHS trusts were required to set up an ‘internal distribution location for the issue of devices to eligible staff members, reporting template, printed copy of the instruction guide and any other written instructions including local information’ [17]. Stakeholder feedback has highlighted that tests may have been made available in staffrooms and similar communal spaces for staff to take home.

## 1.9 Reported LFDs tests

The number of tests reported for NHS trusts represented just a fraction of the number distributed, which may have been due to under-reporting of negative tests. As shown in Fig S1, much lower numbers of LFD tests were reported by NHS trusts in England than the numbers distributed. This is likely to reflect tests taken but not reported, influenced by changes in reporting systems over time and the lack of a consistent, centralised reporting system. The speed of the testing rollout was such that no central reporting system for results of LFD tests was initially available, so each individual trust developed their own reporting system for results and uploaded the data to Public Health England (PHE). Furthermore, and based on findings from our healthcare stakeholder conversations, organisations used a wide range of solutions for reporting LFD results, with some developing their own mobile apps for reporting, some uploading their collated results via Excel spreadsheets and others reporting via emails that included data tables and lists of surnames.

Stakeholders suggested that a possible explanation for the discrepancy between the number of LFD tests distributed and reported could be that there was no rationale stated for reporting negative results, despite this being mandatory and required for the NHS to be able to carry out performance management. Stakeholders also noted that healthcare workers had not associated recording a negative test result with the normal standards required for health-record keeping, where reporting all tests results is required. Therefore, healthcare workers may have viewed reporting a negative COVID-19 test result as being a burden and that they were being required to report something that did not have any impact. It is worth noting that it was unclear from our literature review whether there were evaluated healthcare worker training materials that detailed when and how to report, which may also have impacted reporting rates.

**Fig S1. Cumulative volume of LFD tests distributed and reported across all English healthcare settings.**

The cumulative volume of PCR tests registered and reported has not been included here as, due to a change in the approach to data collection, there was no differentiation by use case (patients versus healthcare workers) for reported PCR tests. Furthermore, the majority of the healthcare workers testing service was conducted using LFD tests. Healthcare workers comprised 95.2% of total LFDs distributed to the NHS and just 9.5% of registered Pillar 1 PCR tests.

An SOP published in September 2021, by NHSE, for primary care and acute trusts, noted that it was important to instruct healthcare workers to report results via a single route, to avoid duplication [19]. Duplicate reporting may have led to additional work pressures on PHE to remove any duplicates from the data. Duplicates were likely to have been reported by healthcare workers who worked for trusts, as they could have double-reported their test results, via both the gov.uk website and the existing reporting route in their trust.

**Fig S2. Weekly volume of LFD tests reported by NHS trust type.** ACT = acute trusts, AMT = ambulance trusts, CMT = community trusts, MHU = mental health trusts. The shaded areas correspond to time periods when the Delta (dark pink) or Omicron (light pink) variants were predominant. Note that the y-axis scales are different for each panel.

Weekly volumes of reported LFD and PCR tests followed similar trends across all healthcare settings except for community trusts. The changes in weekly testing volumes reported shown in Fig S2 and Fig S3 align with changes in healthcare worker testing protocols and variants over time. The number of LFDs reported peaked in all healthcare settings in the week of 9 January 2021, with 667,791 LFDs, likely coinciding with the third national lockdown, which began on 6 January 2021 (Fig S2 and Fig S3). The highest weekly number of LFDs was reported in acute trusts, with 481,415 tests in the week of 9 January 2021. After this time, reporting decreased. The lowest number of LFDs reported was in ambulance trusts, with 6,217 tests in the week of 5 March 2022. With the data that were received it was not possible to point to a potential hypothesis that might explain why a different trend was observed for community trusts.

**Fig S3. Weekly volume of LFD tests reported per worker by trust type (Pillar 1 and 2).** ACT = acute trusts, AMT = ambulance trusts, CMT = community trusts, MHU = mental health trusts. The shaded areas correspond to time periods when the Delta (dark pink) or Omicron (light pink) variants were predominant. Monthly volume of LFD testes were reported in the maintext Fig 1A.

There was an increase in LFD tests reported by healthcare workers in November 2021, likely as a result of changes in policy brought about in response to the emergence of the Omicron variant. The average number of LFD tests was approximately 0.5 tests per worker per week in December 2020 (Fig S3). This decreased to approximately 0.25 tests reported per worker per week over time in all trust types, except for community trusts where it remained at this level until the Omicron variant period, when it increased considerably (up to one test per worker per week). In other trust types, the increase seen during the Omicron period was not so pronounced, although reported LFD tests per worker still increased. The increase in test reporting seen after November 2021 could have been due to the arrival of the more transmissible and increasingly prevalent Omicron variant and a national drive for contacts to test for seven days, irrespective of vaccination status [20].

**Fig S4. Weekly volume of PCR tests reported by trust type.** ACT = acute trusts, AMT = ambulance trusts, CMT = community trusts, MHU = mental health trusts. The shaded areas correspond to time periods when the Delta (dark pink) or Omicron (light pink) variants were prevalent.

The number of tests reported increased from November 2020 to January 2021 (Fig S4), as the rollout of asymptomatic testing for patient-facing healthcare workers began in 34 trusts, benefiting more than 250,000 staff, with the full rollout following soon after. Eligible patient-facing healthcare workers were required to undertake twice-weekly, at-home testing with a self-administered LFD or LAMP test with a confirmatory PCR, ideally before attending work. The rapid acceptance and increase in the use of LFD tests may have been due to the fact that LFDs enabled testing at home, which was described by healthcare workers as preferable to testing at work [21]. A spike in testing was seen around January 2022, coinciding with the Omicron variant period.

There is an inherent tension between local risk-assessment and national guidance. Due to the flexible approach to defining which healthcare workers were eligible for testing, it was challenging to understand the effectiveness of service implementation, as who was included in the specific target population eligible for testing was not reported. Therefore, for this evaluation we had to rely on assumptions related to the total population of the workforce eligible for testing.

The move, in July 2021, to an individual healthcare worker initiated pull model for LFD supply may have contributed to the decrease seen in the number of tests reported after this time According to the November 2020 SOP for asymptomatic testing of healthcare workers in NHS trusts [17], Innova LFDs were supplied to NHS organisations in England to meet the requirements of the staff population to be tested. This was achieved through an agreed ordering schedule with NHSE and constituted a ‘push’ system for ordering tests.

From 5 July 2021, the NHS moved from this push model to an individual staff member-initiated ‘pull’ model for the supply of LFDs [22]. Healthcare workers were now required to order kits for self-testing at home, via the universal testing service, while LFDs for staff were no longer distributed to trusts. Staff were advised to follow this system once they had used up their supply of Innova LFDs. Staff were required to order their own tests online and input the name of the trust where they worked to ensure the tests were registered as belonging to an NHS member of staff. A box of tests was posted to staff at their home address. Although the responsibility for requesting tests moved to NHS staff members, this did not alter the trend in the number of tests distributed and reported.

As noted in our stakeholder interviews, the move to a pull model was implemented to reduce the burden on trusts; however, a major issue was the time needed to build new IT infrastructure within the context of other IT priorities for the government. While the shift to the individual pull model was beneficial from an operational point of view, it removed access to data at the granular level needed for the effective surveillance of nosocomial infections and the corresponding management of staffing. The data that were shared back from UKHSA to trusts were not sufficiently granular for this purpose, due to data sharing constraints and the limitations of the IT capacity.

When universal testing was made available to the entire population in April 2021, there may have been instances when healthcare workers ordered their tests via gov.uk as a member of the public, before this way of ordering and reporting was officially launched for healthcare workers in the summer of 2021. Similarly, healthcare workers with children may have likely utilised the schools testing supply route. As noted in the stakeholder interviews, there is only anecdotal evidence to support this assumption; however, it is likely that this may have been happening when trusts were running out of stock and the new mass supply of tests had not yet arrived. It could also have happened among those groups of healthcare workers who were not eligible for testing as per localised definitions of eligibility. Additionally, where healthcare workers were eligible for testing, the test kits allocated to healthcare workers may have been used by other household members.

## 1.10 The positivity rate of LFD tests followed prevalence estimates

**Fig S5. Weekly proportion of all reported tests that were positive for COVID-19 in English NHS trusts, by type of trust and test type (LFD or PCR).** ACT = acute trusts, AMT = ambulance trusts, CMT = community trusts, MHU = mental health trusts. Grey line: estimated prevalence of COVID-19 in the population, from ONS data [10].

The shaded areas correspond to time periods when the Delta (dark pink) or Omicron (light pink) variants were predominant. The positivity rate of LFD tests generally followed COVID-19 prevalence estimates, while the PCR positivity rate was more erratic, with much higher positivity rates observed, likely due to the small sample size and selection bias. In ambulance and acute trusts, between January and March 2022, LFD positivity was much higher than the population prevalence, indicating possible bias in reporting and higher prevalence in healthcare workers due to the Omicron variant. In addition to declining prevalence, intermittent decreases seen in PCR test reporting were potentially driven by the recommendation to cease home testing for 90 days after any positive result was confirmed by PCR [17].

PCR positivity rates were high in early 2021, before decreasing rapidly, followed by further increases during each of the Delta and Omicron periods of dominance. Only one of these periods, during the dominance of Omicron, reflected prevalence. In January 2022, the requirement for confirmatory PCR tests was suspended [23]. Therefore, the increase in positive PCR test results seen in Figure S1 represents only symptomatic PCR testing, with a much smaller population denominator, likely driven by Omicron prevalence [17].

# S2 Appendix – supplementary figures and tables

## Fig S6. Percentage of monthly FTE days lost due to COVID-19 by trust type and staff group over time.

The fraction is defined as monthly FTE days lost due to COVID-19 divided by monthly FTE days lost in total and then multiplied by 100%. The x-axis presents the time course between March 2020 and March 2022 while y-axis represents the FTE days lost due to COVID-19. The colour of lines represents occupations of NHS staff within the trust. Different panel depicts different types of trusts. ACT, acute trust; AMT, ambulance trust; CMT, community trust; MHU, mental health trust. The raw data was described in the main text and extract from NHS Digital [24].

## Fig S7. Rates of NHS staff absences due to sickness (all causes) by region, March 2020 to March 2022.

The rates are defined as the fraction between the number of days off sick and total workforce days contracted and then multiplied by 100%. The x-axis represents the time course between March 2020 to March 2022 while y-axis represents the FTE days lost in percentage. The colour of lines represents different regions of England, e.g., East of England, London, Midlands, North East and Yorkshire, North West, South East and South West.

## Fig S8. Ratios of monthly FTE days lost due to COVID-19 by staff group.

The ratios are defined as the fraction between monthly FTE days lost due to COVID-19 and monthly FTE days lost in total and then multiplied by 100%. The x-axis represents the time course between March 2020 to March 2022 while y-axis represents the FTE days lost due to COVID-19 in percentage. The colour of lines represents different occupation of NHS staff, e.g., ambulance staff, doctors, midwives, NHS infrastructure support, nurse and health visitors, scientific, therapeutic and technical staff and support to STT staff reported by NHS Digital [24].

## Fig S9. Histogram of the number of LFD tests per healthcare worker by trust-month.

In total, 16,352,554 LFDs were reported from October 2020 to March 2022, across the 136 acute trusts. The x-axis is number of reported LFD tests per healthcare worker within a trust while y-axis is the volume of trust-month, e.g., 1 trust and 1 month gives 1 trust-month while 10 trusts and 2 month gives 20 trust-month.

## Fig S10. Association between the proportion of FTE days lost due to COVID-19 and LFD testing coverage.

Different panels show different time period, e.g., November 2020 to December 2020, January 2021 to July 2021, August 2021 to November 2021, and December 2021 to March 2022.

## Fig S11. Association between the proportion of FTE days lost due to COVID-19 and local COVID-19 prevalence.

Different panels show different time period, e.g., November 2020 to December 2020, January 2021 to July 2021, August 2021 to November 2021, and December 2021 to March 2022.

## Fig S12. Association between the proportion of FTE days lost due to COVID-19 and trust LFD positivity.

Different panels show different time period, e.g., November 2020 to December 2020, January 2021 to July 2021, August 2021 to November 2021, and December 2021 to March 2022.

## Fig S13. Association between the proportion of FTE days lost due to COVID-19 (in logarithmic 2 scale) and trust average income deprivation score.

Different panels show different time period, e.g., November 2020 to December 2020, January 2021 to July 2021, August 2021 to November 2021, and December 2021 to March 2022.

## Fig S14. Association between the proportion of FTE days lost due to COVID-19 (in logarithmic 2 scale) and the prevalence of COVID-19 variants.

Different panels show different time period, e.g., November 2020 to December 2020, January 2021 to July 2021, August 2021 to November 2021, and December 2021 to March 2022.

## Fig S15. Association between the prevalence of COVID-19 and LFD test coverage (in logarithmic 2 scale) for each of the four study periods.

The blue lines and grey shaded areas represent median, and 95% confidence intervals based on a simple linear regression. The p-values, based on Pearson’s correlation test, for each study period were 0.7, 0.11, 0.91 and 0.39, i.e. they were all non-significant.

## Fig S16. Lower and upper limits of the 95% confidence intervals (CIs) for the predicted weekly number of nosocomial infections for various testing scenarios**.**

## Table S1. Summary of time periods during the COVID-19 pandemic and their associated testing policies and epidemiological features.

| Time period | Testing policy | Circulating variant | Vaccination availability |
| --- | --- | --- | --- |
| November 2020–December 2020 | Testing at home with an LFD twice weekly, with a follow-up confirmatory PCR in case of a positive LFD result; testing was voluntary, reporting of all results was a statutory duty; in the case of a positive LFD and PCR result, staff were required to self-isolate in line with the government guidance at that time (10 days). | Wild-type | No vaccine available. |
| January 2021– July 2021 | The testing policy was as before. | Alpha | Vaccination rollout to HCWs was ongoing, but there were no available data on HCWs’ vaccination levels per trust. |
| August 2021–November 2021 | Fully vaccinated members of staff who were identified as a contact of a positive COVID-19 case were no longer expected to isolate, while unvaccinated staff members were required to self-isolate for the full 10-day period. | Delta | High coverage of vaccination; monthly data on vaccination coverage among staff were available at the trust level. |
| December 2021–March 2022 | Confirmatory PCR testing was temporarily suspended for people who received a positive LFD result; individuals who tested positive via an LFD were required to self-isolate for seven days; self-isolation in the case of a positive LFD result was reduced from seven to six days. | Omicron | High coverage of vaccination; monthly data on vaccination coverage among staff were available at the trust level. |

## Table S2. Number of healthcare workers stratified by group across the entire evaluation period (1 October 2020 to 30 March 2022), in all NHS acute trusts.

| **NHS acute trust** | **Doctors** | **Midwives** | **Nurses and health visitors** | **Ambulance staff** | **Scientific, therapeutic and technical staff** | **Support to scientific, therapeutic and technical staff** | **NHS infrastructure support** |
| --- | --- | --- | --- | --- | --- | --- | --- |
| AIREDALE NHS FOUNDATION TRUST | 14.5% | 3.7% | 27.4% | 0.2% | 18.1% | 11.3% | 24.7% |
| ALDER HEY CHILDREN'S NHS FOUNDATION TRUST | 15.3% | 0.0% | 35.7% | 0.0% | 21.1% | 5.8% | 22.1% |
| ASHFORD AND ST PETER'S HOSPITALS NHS FOUNDATION TRUST | 22.1% | 4.2% | 29.1% | 0.1% | 14.3% | 6.6% | 23.6% |
| BARKING, HAVERING AND REDBRIDGE UNIVERSITY HOSPITALS NHS TRUST | 22.2% | 5.7% | 35.9% | 0.0% | 16.5% | 9.4% | 10.4% |
| BARNSLEY HOSPITAL NHS FOUNDATION TRUST | 17.3% | 4.9% | 31.3% | 0.0% | 15.7% | 9.3% | 21.4% |
| BARTS HEALTH NHS TRUST | 22.9% | 4.2% | 34.3% | 0.0% | 16.4% | 5.1% | 17.1% |
| BEDFORDSHIRE HOSPITALS NHS FOUNDATION TRUST | 22.0% | 5.3% | 35.5% | 0.1% | 14.6% | 7.4% | 15.2% |
| BIRMINGHAM WOMEN'S AND CHILDREN'S NHS FOUNDATION TRUST | 14.6% | 7.4% | 34.5% | 0.0% | 20.8% | 5.6% | 17.1% |
| BLACKPOOL TEACHING HOSPITALS NHS FOUNDATION TRUST | 13.2% | 1.9% | 36.4% | 0.0% | 18.9% | 6.8% | 22.8% |
| BOLTON NHS FOUNDATION TRUST | 13.6% | 5.7% | 38.7% | 0.0% | 16.7% | 6.9% | 18.3% |
| BRADFORD TEACHING HOSPITALS NHS FOUNDATION TRUST | 18.2% | 5.1% | 30.2% | 0.0% | 14.3% | 5.8% | 26.5% |
| BRIDGEWATER COMMUNITY HEALTHCARE NHS FOUNDATION TRUST | 7.0% | 2.2% | 47.6% | 0.0% | 22.6% | 3.2% | 17.3% |
| BRIGHTON AND SUSSEX UNIVERSITY HOSPITALS NHS TRUST | 20.8% | 3.8% | 33.4% | 0.0% | 15.8% | 6.1% | 20.1% |
| BUCKINGHAMSHIRE HEALTHCARE NHS TRUST | 19.6% | 3.9% | 37.0% | 0.1% | 18.7% | 7.8% | 12.9% |
| CALDERDALE AND HUDDERSFIELD NHS FOUNDATION TRUST | 15.9% | 4.1% | 32.9% | 0.0% | 16.1% | 9.1% | 21.8% |
| CAMBRIDGE UNIVERSITY HOSPITALS NHS FOUNDATION TRUST | 18.7% | 2.6% | 35.8% | 0.0% | 16.9% | 9.7% | 16.3% |
| CENTRAL LONDON COMMUNITY HEALTHCARE NHS TRUST | 2.1% | 0.0% | 60.2% | 0.1% | 25.5% | 4.4% | 7.7% |
| CHELSEA AND WESTMINSTER HOSPITAL NHS FOUNDATION TRUST | 27.3% | 8.0% | 36.8% | 0.0% | 12.4% | 2.9% | 12.5% |
| CHESTERFIELD ROYAL HOSPITAL NHS FOUNDATION TRUST | 19.5% | 4.5% | 35.5% | 0.0% | 19.1% | 6.8% | 14.6% |
| COUNTESS OF CHESTER HOSPITAL NHS FOUNDATION TRUST | 18.8% | 3.6% | 31.9% | 0.0% | 16.7% | 8.4% | 20.5% |
| COUNTY DURHAM AND DARLINGTON NHS FOUNDATION TRUST | 16.8% | 4.2% | 38.2% | 0.5% | 15.1% | 6.0% | 19.2% |
| CROYDON HEALTH SERVICES NHS TRUST | 22.4% | 4.0% | 36.5% | 0.1% | 14.9% | 6.4% | 15.7% |
| CUMBRIA, NORTHUMBERLAND, TYNE AND WEAR NHS FOUNDATION TRUST | 10.2% | 0.0% | 49.7% | 0.0% | 19.9% | 6.9% | 13.3% |
| DARTFORD AND GRAVESHAM NHS TRUST | 20.4% | 7.1% | 36.9% | 0.0% | 15.7% | 9.3% | 10.6% |
| DONCASTER AND BASSETLAW TEACHING HOSPITALS NHS FOUNDATION TRUST | 14.6% | 3.8% | 30.9% | 0.1% | 13.5% | 10.3% | 26.7% |
| DORSET COUNTY HOSPITAL NHS FOUNDATION TRUST | 20.6% | 4.2% | 32.8% | 0.3% | 15.8% | 6.2% | 20.1% |
| EAST AND NORTH HERTFORDSHIRE NHS TRUST | 18.5% | 3.8% | 30.1% | 0.0% | 12.6% | 6.1% | 28.8% |
| EAST CHESHIRE NHS TRUST | 16.6% | 3.5% | 34.6% | 0.0% | 20.1% | 8.8% | 16.3% |
| EAST KENT HOSPITALS UNIVERSITY NHS FOUNDATION TRUST | 18.3% | 3.8% | 28.2% | 0.0% | 16.0% | 7.9% | 25.7% |
| EAST LANCASHIRE HOSPITALS NHS TRUST | 15.6% | 4.6% | 34.9% | 0.0% | 16.5% | 7.5% | 20.8% |
| EAST SUFFOLK AND NORTH ESSEX NHS FOUNDATION TRUST | 16.9% | 3.5% | 34.5% | 0.1% | 16.4% | 9.7% | 18.8% |
| EAST SUSSEX HEALTHCARE NHS TRUST | 15.2% | 2.9% | 35.6% | 0.1% | 15.3% | 6.6% | 24.3% |
| EPSOM AND ST HELIER UNIVERSITY HOSPITALS NHS TRUST | 23.1% | 5.0% | 34.5% | 0.3% | 17.1% | 6.9% | 13.1% |
| FRIMLEY HEALTH NHS FOUNDATION TRUST | 18.7% | 4.9% | 33.7% | 0.0% | 12.8% | 8.4% | 21.6% |
| GATESHEAD HEALTH NHS FOUNDATION TRUST | 13.8% | 2.6% | 33.3% | 0.0% | 16.6% | 10.5% | 23.1% |
| GEORGE ELIOT HOSPITAL NHS TRUST | 16.5% | 5.2% | 32.3% | 0.2% | 11.8% | 6.5% | 27.5% |
| GLOUCESTERSHIRE HOSPITALS NHS FOUNDATION TRUST | 18.0% | 4.8% | 32.6% | 0.0% | 15.9% | 10.6% | 18.1% |
| GREAT ORMOND STREET HOSPITAL FOR CHILDREN NHS FOUNDATION TRUST | 17.9% | 0.0% | 37.9% | 0.0% | 21.4% | 6.8% | 16.0% |
| GREAT WESTERN HOSPITALS NHS FOUNDATION TRUST | 19.9% | 4.7% | 36.5% | 0.5% | 14.0% | 8.6% | 15.8% |
| GUY'S AND ST THOMAS' NHS FOUNDATION TRUST | 18.0% | 2.0% | 33.2% | 0.0% | 18.0% | 2.8% | 26.0% |
| HAMPSHIRE HOSPITALS NHS FOUNDATION TRUST | 19.1% | 4.6% | 33.3% | 0.0% | 13.8% | 8.2% | 21.0% |
| HARROGATE AND DISTRICT NHS FOUNDATION TRUST | 14.7% | 2.4% | 45.0% | 0.1% | 16.5% | 5.7% | 15.7% |
| HOMERTON UNIVERSITY HOSPITAL NHS FOUNDATION TRUST | 18.7% | 7.3% | 32.1% | 0.1% | 23.0% | 8.5% | 10.4% |
| HOUNSLOW AND RICHMOND COMMUNITY HEALTHCARE NHS TRUST | 1.9% | 0.0% | 39.1% | 0.5% | 34.3% | 9.1% | 15.2% |
| HULL UNIVERSITY TEACHING HOSPITALS NHS TRUST | 17.6% | 2.6% | 29.5% | 0.0% | 14.4% | 8.6% | 27.3% |
| IMPERIAL COLLEGE HEALTHCARE NHS TRUST | 21.0% | 3.5% | 32.8% | 0.0% | 15.7% | 5.4% | 21.5% |
| ISLE OF WIGHT NHS TRUST | 13.3% | 2.6% | 35.3% | 2.7% | 16.4% | 8.4% | 21.3% |
| JAMES PAGET UNIVERSITY HOSPITALS NHS FOUNDATION TRUST | 16.7% | 3.3% | 34.1% | 0.0% | 11.9% | 7.0% | 27.0% |
| KENT COMMUNITY HEALTH NHS FOUNDATION TRUST | 2.8% | 0.0% | 33.3% | 0.5% | 23.5% | 9.0% | 30.9% |
| KETTERING GENERAL HOSPITAL NHS FOUNDATION TRUST | 18.3% | 4.4% | 35.8% | 0.2% | 11.8% | 8.2% | 21.4% |
| KING'S COLLEGE HOSPITAL NHS FOUNDATION TRUST | 26.0% | 4.2% | 39.8% | 0.0% | 16.2% | 3.9% | 9.8% |
| KINGSTON HOSPITAL NHS FOUNDATION TRUST | 24.1% | 7.7% | 36.0% | 0.1% | 13.2% | 6.0% | 12.9% |
| LANCASHIRE TEACHING HOSPITALS NHS FOUNDATION TRUST | 16.6% | 3.0% | 32.7% | 0.0% | 15.8% | 9.4% | 22.5% |
| LEEDS TEACHING HOSPITALS NHS TRUST | 18.0% | 3.0% | 27.8% | 0.0% | 19.6% | 8.4% | 23.1% |
| LEWISHAM AND GREENWICH NHS TRUST | 20.7% | 6.5% | 38.7% | 0.0% | 15.7% | 8.0% | 10.4% |
| LINCOLNSHIRE COMMUNITY HEALTH SERVICES NHS TRUST | 2.0% | 0.9% | 51.6% | 1.5% | 16.4% | 10.3% | 17.3% |
| LIVERPOOL HEART AND CHEST HOSPITAL NHS FOUNDATION TRUST | 11.6% | 0.0% | 37.3% | 0.0% | 17.0% | 7.0% | 27.2% |
| LIVERPOOL UNIVERSITY HOSPITALS NHS FOUNDATION TRUST | 18.0% | 0.0% | 31.3% | 0.0% | 17.4% | 8.9% | 24.3% |
| LIVERPOOL WOMEN'S NHS FOUNDATION TRUST | 15.0% | 24.8% | 32.7% | 0.0% | 6.4% | 3.4% | 17.6% |
| LONDON NORTH WEST UNIVERSITY HEALTHCARE NHS TRUST | 26.5% | 2.1% | 41.5% | 0.0% | 16.1% | 5.2% | 8.5% |
| MAIDSTONE AND TUNBRIDGE WELLS NHS TRUST | 19.3% | 4.9% | 29.3% | 0.1% | 15.6% | 7.0% | 23.7% |
| MANCHESTER UNIVERSITY NHS FOUNDATION TRUST | 16.6% | 3.1% | 37.9% | 0.0% | 19.6% | 8.7% | 14.0% |
| MEDWAY NHS FOUNDATION TRUST | 20.1% | 5.6% | 34.0% | 0.1% | 10.3% | 5.3% | 24.5% |
| MID AND SOUTH ESSEX NHS FOUNDATION TRUST | 19.9% | 4.7% | 35.4% | 0.0% | 12.6% | 6.9% | 20.5% |
| MID CHESHIRE HOSPITALS NHS FOUNDATION TRUST | 12.7% | 3.6% | 34.0% | 0.0% | 18.4% | 10.0% | 21.3% |
| MID YORKSHIRE HOSPITALS NHS TRUST | 16.2% | 3.9% | 30.2% | 0.0% | 17.3% | 9.5% | 23.0% |
| MILTON KEYNES UNIVERSITY HOSPITAL NHS FOUNDATION TRUST | 19.5% | 4.8% | 29.1% | 0.0% | 12.6% | 7.6% | 26.3% |
| MOORFIELDS EYE HOSPITAL NHS FOUNDATION TRUST | 22.0% | 0.0% | 26.1% | 0.0% | 19.9% | 8.7% | 23.3% |
| NORFOLK AND NORWICH UNIVERSITY HOSPITALS NHS FOUNDATION TRUST | 18.9% | 3.5% | 35.5% | 0.1% | 17.7% | 9.7% | 14.6% |
| NORTH BRISTOL NHS TRUST | 17.7% | 4.1% | 30.0% | 0.0% | 16.6% | 10.3% | 21.3% |
| NORTH CUMBRIA INTEGRATED CARE NHS FOUNDATION TRUST | 13.2% | 3.0% | 34.1% | 0.3% | 17.9% | 8.4% | 22.9% |
| NORTH EAST LONDON NHS FOUNDATION TRUST | 5.8% | 0.0% | 41.9% | 0.1% | 29.6% | 7.1% | 15.5% |
| NORTH MIDDLESEX UNIVERSITY HOSPITAL NHS TRUST | 24.5% | 5.7% | 39.5% | 0.0% | 13.6% | 4.9% | 11.7% |
| NORTH TEES AND HARTLEPOOL NHS FOUNDATION TRUST | 13.5% | 3.2% | 30.6% | 0.2% | 15.6% | 8.0% | 28.9% |
| NORTH WEST ANGLIA NHS FOUNDATION TRUST | 19.2% | 4.6% | 37.1% | 0.1% | 15.1% | 7.9% | 16.0% |
| NORTHAMPTON GENERAL HOSPITAL NHS TRUST | 16.9% | 4.6% | 33.5% | 0.2% | 13.3% | 8.5% | 22.9% |
| NORTHERN DEVON HEALTHCARE NHS TRUST | 17.0% | 3.2% | 32.1% | 0.6% | 18.8% | 11.8% | 16.5% |
| NORTHERN LINCOLNSHIRE AND GOOLE NHS FOUNDATION TRUST | 16.0% | 4.0% | 32.7% | 0.3% | 14.3% | 8.4% | 24.2% |
| NORTHUMBRIA HEALTHCARE NHS FOUNDATION TRUST | 13.4% | 2.8% | 37.6% | 0.0% | 20.2% | 5.7% | 20.2% |
| NOTTINGHAM UNIVERSITY HOSPITALS NHS TRUST | 19.0% | 3.7% | 35.4% | 0.0% | 17.2% | 4.3% | 20.4% |
| OXFORD UNIVERSITY HOSPITALS NHS FOUNDATION TRUST | 22.2% | 3.3% | 35.3% | 0.0% | 19.1% | 7.6% | 12.4% |
| PENNINE ACUTE HOSPITALS NHS TRUST | 17.2% | 4.7% | 35.2% | 0.1% | 13.0% | 6.4% | 23.4% |
| PORTSMOUTH HOSPITALS UNIVERSITY NATIONAL HEALTH SERVICE TRUST | 19.8% | 3.7% | 38.0% | 0.0% | 16.3% | 13.7% | 8.5% |
| QUEEN VICTORIA HOSPITAL NHS FOUNDATION TRUST | 24.8% | 0.0% | 28.0% | 0.2% | 17.4% | 6.7% | 22.9% |
| ROYAL BERKSHIRE NHS FOUNDATION TRUST | 20.4% | 4.0% | 35.6% | 0.1% | 14.7% | 5.8% | 19.4% |
| ROYAL BROMPTON & HAREFIELD NHS FOUNDATION TRUST | 15.7% | 0.0% | 43.2% | 0.0% | 18.5% | 4.9% | 17.8% |
| ROYAL CORNWALL HOSPITALS NHS TRUST | 19.8% | 4.3% | 30.9% | 0.0% | 16.2% | 10.3% | 18.5% |
| ROYAL DEVON AND EXETER NHS FOUNDATION TRUST | 15.6% | 2.1% | 33.6% | 0.0% | 16.7% | 8.0% | 23.9% |
| ROYAL FREE LONDON NHS FOUNDATION TRUST | 24.1% | 4.6% | 33.8% | 0.0% | 15.1% | 6.6% | 15.7% |
| ROYAL NATIONAL ORTHOPAEDIC HOSPITAL NHS TRUST | 17.7% | 0.0% | 31.5% | 0.0% | 21.1% | 5.3% | 24.5% |
| ROYAL PAPWORTH HOSPITAL NHS FOUNDATION TRUST | 15.6% | 0.0% | 41.5% | 0.0% | 14.0% | 12.1% | 16.9% |
| ROYAL SURREY COUNTY HOSPITAL NHS FOUNDATION TRUST | 20.8% | 3.2% | 32.6% | 0.1% | 20.7% | 7.6% | 14.9% |
| ROYAL UNITED HOSPITALS BATH NHS FOUNDATION TRUST | 17.5% | 4.4% | 31.2% | 0.0% | 16.2% | 10.4% | 20.3% |
| SALFORD ROYAL NHS FOUNDATION TRUST | 13.3% | 0.0% | 32.9% | 0.0% | 19.9% | 7.6% | 26.2% |
| SALISBURY NHS FOUNDATION TRUST | 16.4% | 3.4% | 32.4% | 0.0% | 17.5% | 7.2% | 23.1% |
| SANDWELL AND WEST BIRMINGHAM HOSPITALS NHS TRUST | 18.3% | 3.8% | 34.4% | 0.1% | 13.6% | 5.4% | 24.3% |
| SHEFFIELD CHILDREN'S NHS FOUNDATION TRUST | 16.0% | 0.0% | 37.2% | 0.0% | 23.0% | 9.7% | 14.1% |
| SHEFFIELD TEACHING HOSPITALS NHS FOUNDATION TRUST | 15.3% | 2.1% | 32.7% | 0.0% | 18.9% | 7.5% | 23.4% |
| SHERWOOD FOREST HOSPITALS NHS FOUNDATION TRUST | 19.0% | 3.8% | 33.7% | 0.1% | 15.0% | 8.9% | 19.5% |
| SHREWSBURY AND TELFORD HOSPITAL NHS TRUST | 16.5% | 5.6% | 32.0% | 0.1% | 15.3% | 9.5% | 21.0% |
| SOMERSET NHS FOUNDATION TRUST | 13.1% | 2.4% | 34.6% | 0.2% | 19.6% | 5.9% | 24.1% |
| SOUTH TEES HOSPITALS NHS FOUNDATION TRUST | 17.5% | 3.3% | 38.8% | 0.1% | 18.4% | 8.9% | 13.0% |
| SOUTH TYNESIDE AND SUNDERLAND NHS FOUNDATION TRUST | 15.3% | 2.6% | 41.6% | 0.0% | 16.4% | 5.4% | 18.6% |
| SOUTH WARWICKSHIRE NHS FOUNDATION TRUST | 13.2% | 4.0% | 41.6% | 0.0% | 19.6% | 6.9% | 14.6% |
| SOUTHPORT AND ORMSKIRK HOSPITAL NHS TRUST | 16.6% | 4.9% | 30.9% | 0.0% | 15.4% | 7.4% | 24.7% |
| ST GEORGE'S UNIVERSITY HOSPITALS NHS FOUNDATION TRUST | 21.5% | 2.7% | 33.1% | 0.0% | 20.2% | 9.3% | 13.2% |
| ST HELENS AND KNOWSLEY TEACHING HOSPITALS NHS TRUST | 17.3% | 3.1% | 32.3% | 0.0% | 13.8% | 7.8% | 25.7% |
| STOCKPORT NHS FOUNDATION TRUST | 14.3% | 4.1% | 35.2% | 0.0% | 16.8% | 6.7% | 22.8% |
| SURREY AND SUSSEX HEALTHCARE NHS TRUST | 20.5% | 5.1% | 36.2% | 0.6% | 12.6% | 6.2% | 18.9% |
| SUSSEX COMMUNITY NHS FOUNDATION TRUST | 2.4% | 0.0% | 42.1% | 0.3% | 27.1% | 9.2% | 18.9% |
| TAMESIDE AND GLOSSOP INTEGRATED CARE NHS FOUNDATION TRUST | 15.3% | 4.3% | 34.2% | 0.0% | 15.6% | 7.7% | 22.8% |
| THE CLATTERBRIDGE CANCER CENTRE NHS FOUNDATION TRUST | 9.8% | 0.0% | 30.8% | 0.0% | 31.1% | 7.8% | 20.6% |
| THE DUDLEY GROUP NHS FOUNDATION TRUST | 17.8% | 4.5% | 41.3% | 0.4% | 16.8% | 8.7% | 10.5% |
| THE HILLINGDON HOSPITALS NHS FOUNDATION TRUST | 20.2% | 6.0% | 31.7% | 0.0% | 11.2% | 6.7% | 24.2% |
| THE NEWCASTLE UPON TYNE HOSPITALS NHS FOUNDATION TRUST | 17.7% | 2.4% | 36.9% | 0.0% | 18.8% | 7.6% | 16.6% |
| THE PRINCESS ALEXANDRA HOSPITAL NHS TRUST | 22.0% | 6.1% | 32.3% | 0.2% | 11.9% | 5.4% | 22.2% |
| THE QUEEN ELIZABETH HOSPITAL, KING'S LYNN, NHS FOUNDATION TRUST | 20.2% | 3.7% | 33.7% | 0.2% | 11.8% | 6.1% | 24.3% |
| THE ROBERT JONES AND AGNES HUNT ORTHOPAEDIC HOSPITAL NHS FOUNDATION TRUST | 12.6% | 0.0% | 28.4% | 0.0% | 20.4% | 9.2% | 29.4% |
| THE ROTHERHAM NHS FOUNDATION TRUST | 15.0% | 3.4% | 34.2% | 0.2% | 18.9% | 10.2% | 18.0% |
| THE ROYAL MARSDEN NHS FOUNDATION TRUST | 14.5% | 0.0% | 32.2% | 0.0% | 23.5% | 5.6% | 24.3% |
| THE ROYAL ORTHOPAEDIC HOSPITAL NHS FOUNDATION TRUST | 15.5% | 0.0% | 32.1% | 0.0% | 15.1% | 5.4% | 31.9% |
| THE ROYAL WOLVERHAMPTON NHS TRUST | 15.8% | 3.3% | 32.6% | 0.0% | 17.0% | 11.9% | 19.4% |
| THE WALTON CENTRE NHS FOUNDATION TRUST | 17.4% | 0.0% | 36.6% | 0.0% | 20.3% | 0.7% | 25.0% |
| TORBAY AND SOUTH DEVON NHS FOUNDATION TRUST | 13.1% | 2.1% | 26.1% | 0.2% | 21.9% | 11.1% | 25.6% |
| UNITED LINCOLNSHIRE HOSPITALS NHS TRUST | 20.4% | 3.9% | 32.6% | 0.1% | 14.3% | 8.7% | 20.0% |
| UNIVERSITY COLLEGE LONDON HOSPITALS NHS FOUNDATION TRUST | 23.8% | 3.0% | 34.8% | 0.0% | 18.3% | 4.1% | 16.1% |
| UNIVERSITY HOSPITAL SOUTHAMPTON NHS FOUNDATION TRUST | 21.9% | 3.0% | 39.3% | 0.2% | 16.6% | 5.6% | 13.4% |
| UNIVERSITY HOSPITALS BIRMINGHAM NHS FOUNDATION TRUST | 17.7% | 2.6% | 32.6% | 0.0% | 16.4% | 7.0% | 23.8% |
| UNIVERSITY HOSPITALS BRISTOL AND WESTON NHS FOUNDATION TRUST | 19.8% | 2.5% | 34.5% | 0.0% | 17.0% | 6.3% | 19.8% |
| UNIVERSITY HOSPITALS COVENTRY AND WARWICKSHIRE NHS TRUST | 19.5% | 3.7% | 37.9% | 0.0% | 18.1% | 10.3% | 10.5% |
| UNIVERSITY HOSPITALS DORSET NHS FOUNDATION TRUST | 19.1% | 2.8% | 34.4% | 0.1% | 17.5% | 8.7% | 17.5% |
| UNIVERSITY HOSPITALS OF DERBY AND BURTON NHS FOUNDATION TRUST | 18.7% | 4.1% | 35.1% | 0.1% | 16.5% | 9.7% | 15.8% |
| UNIVERSITY HOSPITALS OF LEICESTER NHS TRUST | 19.8% | 3.5% | 32.4% | 0.1% | 14.5% | 6.5% | 23.4% |
| UNIVERSITY HOSPITALS OF MORECAMBE BAY NHS FOUNDATION TRUST | 13.6% | 3.7% | 36.9% | 0.1% | 15.2% | 6.1% | 24.4% |
| UNIVERSITY HOSPITALS OF NORTH MIDLANDS NHS TRUST | 18.7% | 3.1% | 36.8% | 0.0% | 15.9% | 8.2% | 17.3% |
| UNIVERSITY HOSPITALS PLYMOUTH NHS TRUST | 18.6% | 2.3% | 31.6% | 0.0% | 15.5% | 7.3% | 24.7% |
| WALSALL HEALTHCARE NHS TRUST | 15.7% | 4.8% | 34.6% | 0.2% | 13.3% | 6.7% | 24.6% |
| WARRINGTON AND HALTON TEACHING HOSPITALS NHS FOUNDATION TRUST | 15.8% | 3.7% | 29.1% | 0.1% | 18.0% | 10.4% | 22.9% |
| WEST HERTFORDSHIRE HOSPITALS NHS TRUST | 21.9% | 4.3% | 34.8% | 0.0% | 14.7% | 8.4% | 15.9% |
| WEST SUFFOLK NHS FOUNDATION TRUST | 17.2% | 3.3% | 33.0% | 0.0% | 18.5% | 6.2% | 21.8% |
| WESTERN SUSSEX HOSPITALS NHS FOUNDATION TRUST | 17.6% | 4.2% | 32.4% | 0.1% | 14.1% | 9.8% | 21.8% |
| WHITTINGTON HEALTH NHS TRUST | 16.0% | 4.6% | 27.4% | 0.0% | 23.3% | 7.3% | 21.5% |
| WIRRAL UNIVERSITY TEACHING HOSPITAL NHS FOUNDATION TRUST | 16.4% | 3.2% | 32.3% | 0.0% | 16.0% | 7.0% | 25.0% |
| WORCESTERSHIRE ACUTE HOSPITALS NHS TRUST | 17.8% | 5.1% | 36.2% | 0.1% | 17.4% | 8.1% | 15.4% |
| WRIGHTINGTON, WIGAN AND LEIGH NHS FOUNDATION TRUST | 14.2% | 3.0% | 34.5% | 0.1% | 15.3% | 6.1% | 26.7% |
| WYE VALLEY NHS TRUST | 17.6% | 3.3% | 35.0% | 0.1% | 16.4% | 10.8% | 16.9% |
| YEOVIL DISTRICT HOSPITAL NHS FOUNDATION TRUST | 20.0% | 3.5% | 33.7% | 0.4% | 12.6% | 7.1% | 22.7% |
| YORK TEACHING HOSPITAL NHS FOUNDATION TRUST | 15.8% | 3.1% | 29.4% | 0.0% | 16.5% | 9.3% | 25.9% |

## Table S3. Number of LFD tests per patient-facing staff member for the four study periods in all NHS acute trusts.

|  | **November 2020–December 2020** | | | **January 2021–July 2021** | | | **August 2021–November 2021** | | | **December 2021–March 2022** | | |
| --- | --- | --- | --- | --- | --- | --- | --- | --- | --- | --- | --- | --- |
| **NHS acute trust** | **Median** | **Max** | **Min** | **Median** | **Max** | **Min** | **Median** | **Max** | **Min** | **Median** | **Max** | **Min** |
| AIREDALE NHS FOUNDATION TRUST | 0.62 | 1.24 | 0.00 | 1.12 | 2.07 | 0.79 | 0.52 | 0.69 | 0.39 | 0.35 | 0.68 | 0.17 |
| ALDER HEY CHILDREN'S NHS FOUNDATION TRUST | 2.80 | 5.50 | 0.10 | 0.17 | 4.32 | 0.08 | 0.03 | 0.05 | 0.03 | 0.02 | 0.07 | 0.01 |
| ASHFORD AND ST PETER'S HOSPITALS NHS FOUNDATION TRUST | 0.89 | 1.78 | 0.00 | 4.18 | 4.84 | 4.10 | 3.31 | 3.85 | 2.98 | 3.14 | 3.42 | 2.46 |
| BARKING, HAVERING AND REDBRIDGE UNIVERSITY HOSPITALS NHS TRUST | 1.01 | 1.87 | 0.15 | 0.49 | 1.88 | 0.41 | 0.18 | 0.35 | 0.14 | 0.25 | 0.39 | 0.19 |
| BARNSLEY HOSPITAL NHS FOUNDATION TRUST | 0.24 | 0.48 | 0.00 | 0.05 | 0.14 | 0.04 | 0.33 | 0.35 | 0.32 | 0.39 | 0.64 | 0.28 |
| BARTS HEALTH NHS TRUST | 0.37 | 0.75 | 0.00 | 0.79 | 1.48 | 0.59 | 0.75 | 0.79 | 0.73 | 1.22 | 1.60 | 0.87 |
| BEDFORDSHIRE HOSPITALS NHS FOUNDATION TRUST | 0.62 | 1.24 | 0.00 | 1.42 | 2.58 | 0.83 | 0.36 | 0.39 | 0.34 | 0.56 | 0.74 | 0.40 |
| BIRMINGHAM WOMEN'S AND CHILDREN'S NHS FOUNDATION TRUST | 0.71 | 1.38 | 0.03 | 0.34 | 1.31 | 0.11 | 0.01 | 0.03 | 0.01 | 0.01 | 0.02 | 0.01 |
| BLACKPOOL TEACHING HOSPITALS NHS FOUNDATION TRUST | 0.00 | 0.00 | 0.00 | 0.00 | 0.04 | 0.00 | 0.04 | 0.04 | 0.04 | 0.09 | 0.22 | 0.07 |
| BOLTON NHS FOUNDATION TRUST | 0.85 | 1.70 | 0.00 | 1.59 | 3.08 | 0.98 | 0.49 | 0.76 | 0.26 | 0.33 | 0.51 | 0.19 |
| BRADFORD TEACHING HOSPITALS NHS FOUNDATION TRUST | 0.57 | 1.15 | 0.00 | 0.79 | 1.90 | 0.64 | 0.46 | 0.57 | 0.36 | 0.32 | 0.82 | 0.18 |
| BRIDGEWATER COMMUNITY HEALTHCARE NHS FOUNDATION TRUST | 0.00 | 0.00 | 0.00 | 0.00 | 0.01 | 0.00 | 0.01 | 0.01 | 0.01 | 0.02 | 0.04 | 0.02 |
| BRIGHTON AND SUSSEX UNIVERSITY HOSPITALS NHS TRUST | 0.88 | 1.71 | 0.04 | 1.24 | 2.70 | 0.67 | 0.51 | 0.61 | 0.44 | 0.48 | 0.54 | 0.40 |
| BUCKINGHAMSHIRE HEALTHCARE NHS TRUST | 0.92 | 1.84 | 0.01 | 2.07 | 2.76 | 1.49 | 0.80 | 1.23 | 0.45 | 0.25 | 0.36 | 0.12 |
| CALDERDALE AND HUDDERSFIELD NHS FOUNDATION TRUST | 0.16 | 0.33 | 0.00 | 0.71 | 1.28 | 0.68 | 0.45 | 0.63 | 0.15 | 0.25 | 0.42 | 0.20 |
| CAMBRIDGE UNIVERSITY HOSPITALS NHS FOUNDATION TRUST | 0.00 | 0.00 | 0.00 | 0.00 | 0.04 | 0.00 | 0.08 | 0.14 | 0.07 | 0.31 | 0.43 | 0.27 |
| CENTRAL LONDON COMMUNITY HEALTHCARE NHS TRUST | 0.00 | 0.00 | 0.00 | 0.00 | 0.00 | 0.00 | 0.00 | 0.00 | 0.00 | 0.00 | 0.00 | 0.00 |
| CHELSEA AND WESTMINSTER HOSPITAL NHS FOUNDATION TRUST | 2.05 | 3.61 | 0.50 | 0.89 | 3.27 | 0.73 | 1.06 | 1.40 | 0.81 | 1.43 | 1.83 | 0.74 |
| CHESTERFIELD ROYAL HOSPITAL NHS FOUNDATION TRUST | 1.04 | 2.08 | 0.00 | 1.42 | 2.99 | 1.04 | 0.71 | 0.92 | 0.55 | 0.51 | 0.65 | 0.30 |
| COUNTESS OF CHESTER HOSPITAL NHS FOUNDATION TRUST | 0.60 | 1.21 | 0.00 | 0.06 | 1.68 | 0.01 | 0.06 | 0.06 | 0.05 | 0.16 | 0.27 | 0.11 |
| COUNTY DURHAM AND DARLINGTON NHS FOUNDATION TRUST | 0.30 | 0.59 | 0.00 | 1.16 | 1.65 | 0.91 | 0.82 | 0.95 | 0.60 | 0.86 | 1.42 | 0.59 |
| CROYDON HEALTH SERVICES NHS TRUST | 0.22 | 0.43 | 0.00 | 0.63 | 0.90 | 0.41 | 0.37 | 0.63 | 0.30 | 0.24 | 0.49 | 0.15 |
| CUMBRIA, NORTHUMBERLAND, TYNE AND WEAR NHS FOUNDATION TRUST | 0.00 | 0.00 | 0.00 | 0.00 | 0.00 | 0.00 | 0.00 | 0.00 | 0.00 | 0.00 | 0.00 | 0.00 |
| DARTFORD AND GRAVESHAM NHS TRUST | 0.70 | 1.41 | 0.00 | 1.94 | 2.77 | 1.27 | 1.13 | 1.34 | 0.95 | 1.02 | 1.33 | 0.55 |
| DONCASTER AND BASSETLAW TEACHING HOSPITALS NHS FOUNDATION TRUST | 1.23 | 2.47 | 0.00 | 2.50 | 3.46 | 1.87 | 1.17 | 1.46 | 1.01 | 1.10 | 1.42 | 0.81 |
| DORSET COUNTY HOSPITAL NHS FOUNDATION TRUST | 0.79 | 1.57 | 0.01 | 1.15 | 2.82 | 0.80 | 0.74 | 0.97 | 0.61 | 1.21 | 1.59 | 1.05 |
| EAST AND NORTH HERTFORDSHIRE NHS TRUST | 0.70 | 1.38 | 0.01 | 1.44 | 2.14 | 1.19 | 0.97 | 1.13 | 0.82 | 1.30 | 1.73 | 1.22 |
| EAST CHESHIRE NHS TRUST | 0.77 | 1.54 | 0.00 | 1.69 | 2.28 | 1.26 | 1.31 | 1.37 | 1.19 | 0.88 | 1.60 | 0.35 |
| EAST KENT HOSPITALS UNIVERSITY NHS FOUNDATION TRUST | 0.90 | 1.80 | 0.00 | 2.09 | 3.29 | 1.94 | 1.60 | 1.84 | 1.49 | 1.65 | 2.17 | 1.32 |
| EAST LANCASHIRE HOSPITALS NHS TRUST | 0.49 | 0.96 | 0.02 | 0.39 | 0.88 | 0.09 | 0.07 | 0.07 | 0.07 | 0.11 | 0.21 | 0.09 |
| EAST SUFFOLK AND NORTH ESSEX NHS FOUNDATION TRUST | 1.30 | 2.57 | 0.03 | 2.01 | 2.54 | 1.71 | 1.20 | 1.63 | 0.97 | 0.99 | 1.18 | 0.76 |
| EAST SUSSEX HEALTHCARE NHS TRUST | 0.90 | 1.80 | 0.00 | 1.40 | 2.99 | 1.04 | 0.91 | 1.11 | 0.80 | 0.98 | 1.24 | 0.88 |
| EPSOM AND ST HELIER UNIVERSITY HOSPITALS NHS TRUST | 1.04 | 1.91 | 0.17 | 0.89 | 1.70 | 0.71 | 0.88 | 1.09 | 0.73 | 1.52 | 2.14 | 1.17 |
| FRIMLEY HEALTH NHS FOUNDATION TRUST | 1.88 | 3.76 | 0.01 | 2.45 | 3.78 | 1.82 | 1.16 | 1.44 | 1.00 | 1.50 | 1.80 | 1.33 |
| GATESHEAD HEALTH NHS FOUNDATION TRUST | 0.27 | 0.53 | 0.00 | 0.30 | 0.56 | 0.21 | 0.21 | 0.24 | 0.19 | 0.24 | 0.35 | 0.15 |
| GEORGE ELIOT HOSPITAL NHS TRUST | 0.87 | 1.74 | 0.00 | 1.06 | 1.87 | 0.76 | 0.42 | 0.72 | 0.17 | 0.22 | 0.33 | 0.14 |
| GLOUCESTERSHIRE HOSPITALS NHS FOUNDATION TRUST | 0.02 | 0.02 | 0.02 | 0.46 | 0.82 | 0.04 | 0.35 | 0.39 | 0.30 | 0.48 | 0.59 | 0.40 |
| GREAT ORMOND STREET HOSPITAL FOR CHILDREN NHS FOUNDATION TRUST | 0.33 | 0.67 | 0.00 | 0.39 | 1.75 | 0.24 | 0.27 | 0.29 | 0.26 | 0.72 | 0.86 | 0.05 |
| GREAT WESTERN HOSPITALS NHS FOUNDATION TRUST | 1.01 | 1.99 | 0.03 | 0.79 | 1.90 | 0.40 | 0.35 | 0.36 | 0.30 | 0.60 | 0.84 | 0.52 |
| GUY'S AND ST THOMAS' NHS FOUNDATION TRUST | 1.21 | 2.39 | 0.02 | 1.54 | 2.30 | 1.35 | 0.94 | 1.43 | 0.69 | 1.25 | 1.48 | 1.06 |
| HAMPSHIRE HOSPITALS NHS FOUNDATION TRUST | 0.68 | 1.36 | 0.00 | 0.95 | 2.10 | 0.00 | 0.45 | 0.59 | 0.29 | 0.69 | 0.85 | 0.51 |
| HARROGATE AND DISTRICT NHS FOUNDATION TRUST | 1.06 | 2.11 | 0.00 | 1.23 | 1.88 | 0.84 | 0.20 | 0.35 | 0.19 | 0.28 | 0.37 | 0.22 |
| HOMERTON UNIVERSITY HOSPITAL NHS FOUNDATION TRUST | 0.71 | 1.43 | 0.00 | 0.95 | 2.09 | 0.83 | 0.83 | 0.93 | 0.81 | 0.99 | 1.20 | 0.81 |
| HOUNSLOW AND RICHMOND COMMUNITY HEALTHCARE NHS TRUST | 0.00 | 0.00 | 0.00 | 0.00 | 0.05 | 0.00 | 0.11 | 0.13 | 0.10 | 0.32 | 0.45 | 0.24 |
| HULL UNIVERSITY TEACHING HOSPITALS NHS TRUST | 0.70 | 1.39 | 0.00 | 0.91 | 1.90 | 0.06 | 0.09 | 0.10 | 0.05 | 0.32 | 0.41 | 0.14 |
| IMPERIAL COLLEGE HEALTHCARE NHS TRUST | 1.52 | 2.76 | 0.28 | 1.22 | 3.03 | 0.80 | 1.10 | 1.22 | 1.02 | 0.26 | 0.53 | 0.13 |
| ISLE OF WIGHT NHS TRUST | 0.00 | 0.00 | 0.00 | 0.00 | 0.06 | 0.00 | 0.10 | 0.11 | 0.06 | 0.44 | 0.51 | 0.29 |
| JAMES PAGET UNIVERSITY HOSPITALS NHS FOUNDATION TRUST | 2.19 | 4.19 | 0.19 | 2.36 | 3.67 | 1.07 | 0.23 | 0.62 | 0.15 | 0.19 | 0.27 | 0.16 |
| KENT COMMUNITY HEALTH NHS FOUNDATION TRUST | 0.00 | 0.00 | 0.00 | 0.00 | 0.00 | 0.00 | 0.00 | 0.00 | 0.00 | 0.00 | 0.00 | 0.00 |
| KETTERING GENERAL HOSPITAL NHS FOUNDATION TRUST | 0.87 | 1.71 | 0.03 | 0.87 | 1.24 | 0.67 | 0.59 | 0.65 | 0.49 | 0.43 | 0.58 | 0.32 |
| KING'S COLLEGE HOSPITAL NHS FOUNDATION TRUST | 0.42 | 0.83 | 0.00 | 0.22 | 0.68 | 0.20 | 0.17 | 0.21 | 0.16 | 0.35 | 0.49 | 0.21 |
| KINGSTON HOSPITAL NHS FOUNDATION TRUST | 0.00 | 0.00 | 0.00 | 0.78 | 1.45 | 0.41 | 0.24 | 0.25 | 0.23 | 0.54 | 0.68 | 0.37 |
| LANCASHIRE TEACHING HOSPITALS NHS FOUNDATION TRUST | 1.05 | 2.09 | 0.00 | 1.36 | 1.76 | 0.33 | 0.68 | 0.95 | 0.58 | 0.77 | 1.33 | 0.69 |
| LEEDS TEACHING HOSPITALS NHS TRUST | 0.20 | 0.40 | 0.00 | 1.52 | 1.94 | 1.17 | 0.83 | 1.02 | 0.66 | 0.61 | 0.79 | 0.44 |
| LEWISHAM AND GREENWICH NHS TRUST | 1.11 | 2.21 | 0.00 | 1.22 | 2.70 | 0.91 | 0.90 | 1.18 | 0.74 | 1.07 | 1.70 | 0.75 |
| LINCOLNSHIRE COMMUNITY HEALTH SERVICES NHS TRUST | 0.00 | 0.00 | 0.00 | 0.00 | 0.05 | 0.00 | 0.07 | 0.08 | 0.07 | 0.19 | 0.22 | 0.11 |
| LIVERPOOL HEART AND CHEST HOSPITAL NHS FOUNDATION TRUST | 1.71 | 3.40 | 0.02 | 0.09 | 3.35 | 0.02 | 0.76 | 1.61 | 0.08 | 1.90 | 2.42 | 1.58 |
| LIVERPOOL UNIVERSITY HOSPITALS NHS FOUNDATION TRUST | 0.03 | 0.05 | 0.00 | 0.03 | 0.65 | 0.01 | 0.04 | 0.05 | 0.03 | 0.12 | 0.19 | 0.09 |
| LIVERPOOL WOMEN'S NHS FOUNDATION TRUST | 1.59 | 3.08 | 0.10 | 0.65 | 2.91 | 0.06 | 0.51 | 0.58 | 0.35 | 0.92 | 1.24 | 0.43 |
| LONDON NORTH WEST UNIVERSITY HEALTHCARE NHS TRUST | 0.51 | 0.92 | 0.10 | 0.61 | 1.35 | 0.34 | 0.17 | 0.17 | 0.16 | 0.42 | 0.60 | 0.32 |
| MAIDSTONE AND TUNBRIDGE WELLS NHS TRUST | 0.78 | 1.54 | 0.01 | 2.85 | 3.58 | 2.36 | 2.79 | 2.99 | 2.50 | 2.84 | 3.77 | 2.40 |
| MANCHESTER UNIVERSITY NHS FOUNDATION TRUST | 1.11 | 2.20 | 0.02 | 0.94 | 2.60 | 0.81 | 0.94 | 1.14 | 0.86 | 1.17 | 1.85 | 0.60 |
| MEDWAY NHS FOUNDATION TRUST | 0.66 | 1.31 | 0.00 | 1.13 | 2.76 | 0.80 | 1.35 | 1.42 | 1.27 | 1.48 | 1.64 | 1.21 |
| MID AND SOUTH ESSEX NHS FOUNDATION TRUST | 0.89 | 1.64 | 0.14 | 0.46 | 4.14 | 0.24 | 0.64 | 0.69 | 0.59 | 1.34 | 1.91 | 1.06 |
| MID CHESHIRE HOSPITALS NHS FOUNDATION TRUST | 1.65 | 3.30 | 0.00 | 4.70 | 5.66 | 1.30 | 1.15 | 2.08 | 0.98 | 3.11 | 4.20 | 2.48 |
| MID YORKSHIRE HOSPITALS NHS TRUST | 0.32 | 0.64 | 0.00 | 0.63 | 1.08 | 0.50 | 0.78 | 0.89 | 0.58 | 1.77 | 2.73 | 1.42 |
| MILTON KEYNES UNIVERSITY HOSPITAL NHS FOUNDATION TRUST | 3.69 | 6.85 | 0.53 | 1.71 | 3.90 | 1.22 | 1.13 | 1.66 | 0.92 | 1.14 | 1.45 | 0.52 |
| MOORFIELDS EYE HOSPITAL NHS FOUNDATION TRUST | 0.32 | 0.63 | 0.00 | 0.32 | 1.47 | 0.20 | 0.24 | 0.27 | 0.22 | 0.36 | 0.51 | 0.16 |
| NORFOLK AND NORWICH UNIVERSITY HOSPITALS NHS FOUNDATION TRUST | 0.92 | 1.83 | 0.00 | 0.72 | 1.62 | 0.19 | 0.14 | 0.16 | 0.13 | 0.22 | 0.29 | 0.20 |
| NORTH BRISTOL NHS TRUST | 0.88 | 1.73 | 0.03 | 1.75 | 2.50 | 1.25 | 1.04 | 1.81 | 0.50 | 0.66 | 0.85 | 0.51 |
| NORTH CUMBRIA INTEGRATED CARE NHS FOUNDATION TRUST | 0.00 | 0.00 | 0.00 | 0.00 | 0.03 | 0.00 | 0.18 | 0.21 | 0.10 | 0.39 | 0.57 | 0.27 |
| NORTH EAST LONDON NHS FOUNDATION TRUST | 0.00 | 0.00 | 0.00 | 0.00 | 0.00 | 0.00 | 0.00 | 0.00 | 0.00 | 0.00 | 0.01 | 0.00 |
| NORTH MIDDLESEX UNIVERSITY HOSPITAL NHS TRUST | 0.23 | 0.46 | 0.00 | 0.03 | 0.50 | 0.02 | 0.05 | 0.06 | 0.05 | 0.16 | 0.24 | 0.12 |
| NORTH TEES AND HARTLEPOOL NHS FOUNDATION TRUST | 0.33 | 0.67 | 0.00 | 0.91 | 1.54 | 0.53 | 0.51 | 0.73 | 0.41 | 0.33 | 0.56 | 0.20 |
| NORTH WEST ANGLIA NHS FOUNDATION TRUST | 1.29 | 2.57 | 0.01 | 3.02 | 3.72 | 1.08 | 0.56 | 0.64 | 0.56 | 0.77 | 1.02 | 0.61 |
| NORTHAMPTON GENERAL HOSPITAL NHS TRUST | 0.75 | 1.49 | 0.00 | 1.27 | 1.50 | 0.65 | 0.00 | 0.21 | 0.00 | 0.00 | 0.00 | 0.00 |
| NORTHERN DEVON HEALTHCARE NHS TRUST | 0.37 | 0.75 | 0.00 | 1.35 | 1.99 | 1.05 | 0.69 | 0.72 | 0.63 | 0.74 | 0.84 | 0.50 |
| NORTHERN LINCOLNSHIRE AND GOOLE NHS FOUNDATION TRUST | 0.60 | 1.21 | 0.00 | 2.60 | 3.13 | 1.97 | 2.25 | 2.56 | 1.84 | 1.41 | 2.38 | 0.58 |
| NORTHUMBRIA HEALTHCARE NHS FOUNDATION TRUST | 1.04 | 2.07 | 0.00 | 3.31 | 3.54 | 2.97 | 2.45 | 2.66 | 2.36 | 2.11 | 2.78 | 1.21 |
| NOTTINGHAM UNIVERSITY HOSPITALS NHS TRUST | 0.44 | 0.88 | 0.00 | 1.17 | 1.45 | 0.96 | 1.01 | 1.17 | 0.87 | 0.91 | 1.18 | 0.40 |
| OXFORD UNIVERSITY HOSPITALS NHS FOUNDATION TRUST | 1.14 | 2.27 | 0.01 | 1.97 | 3.11 | 1.17 | 1.08 | 1.17 | 1.01 | 1.28 | 1.74 | 1.13 |
| PENNINE ACUTE HOSPITALS NHS TRUST | 0.31 | 0.59 | 0.03 | 0.38 | 0.65 | 0.31 | 0.42 | 0.46 | 0.28 | 0.32 | 0.56 | 0.17 |
| PORTSMOUTH HOSPITALS UNIVERSITY NATIONAL HEALTH SERVICE TRUST | 1.18 | 2.24 | 0.13 | 2.73 | 2.93 | 2.28 | 1.81 | 2.49 | 1.49 | 1.70 | 2.19 | 1.12 |
| QUEEN VICTORIA HOSPITAL NHS FOUNDATION TRUST | 0.01 | 0.01 | 0.00 | 0.10 | 0.14 | 0.02 | 0.00 | 0.01 | 0.00 | 0.00 | 0.00 | 0.00 |
| ROYAL BERKSHIRE NHS FOUNDATION TRUST | 0.24 | 0.48 | 0.00 | 0.53 | 0.67 | 0.42 | 0.37 | 0.40 | 0.35 | 0.48 | 0.64 | 0.32 |
| ROYAL BROMPTON & HAREFIELD NHS FOUNDATION TRUST | 3.40 | 6.77 | 0.02 | 0.00 | 3.31 | 0.00 | 0.00 | 0.00 | 0.00 | 0.00 | 0.00 | 0.00 |
| ROYAL CORNWALL HOSPITALS NHS TRUST | 0.55 | 1.11 | 0.00 | 1.50 | 1.65 | 1.47 | 0.09 | 0.36 | 0.09 | 0.19 | 0.22 | 0.14 |
| ROYAL DEVON AND EXETER NHS FOUNDATION TRUST | 1.40 | 2.74 | 0.07 | 2.13 | 2.50 | 1.76 | 0.47 | 0.84 | 0.41 | 0.42 | 0.48 | 0.31 |
| ROYAL FREE LONDON NHS FOUNDATION TRUST | 1.26 | 2.52 | 0.00 | 0.57 | 2.28 | 0.41 | 0.50 | 0.57 | 0.46 | 0.69 | 0.88 | 0.43 |
| ROYAL NATIONAL ORTHOPAEDIC HOSPITAL NHS TRUST | 0.45 | 0.82 | 0.08 | 0.98 | 1.55 | 0.64 | 0.11 | 0.98 | 0.00 | 0.00 | 0.00 | 0.00 |
| ROYAL PAPWORTH HOSPITAL NHS FOUNDATION TRUST | 1.03 | 2.05 | 0.00 | 1.22 | 2.31 | 0.56 | 0.51 | 0.70 | 0.09 | 0.00 | 0.00 | 0.00 |
| ROYAL SURREY COUNTY HOSPITAL NHS FOUNDATION TRUST | 0.71 | 1.42 | 0.00 | 5.58 | 7.22 | 3.75 | 4.60 | 5.43 | 4.14 | 5.47 | 7.36 | 4.99 |
| ROYAL UNITED HOSPITALS BATH NHS FOUNDATION TRUST | 0.56 | 1.12 | 0.01 | 0.59 | 1.03 | 0.41 | 0.46 | 0.49 | 0.43 | 0.72 | 0.91 | 0.65 |
| SALFORD ROYAL NHS FOUNDATION TRUST | 0.36 | 0.71 | 0.00 | 0.53 | 1.13 | 0.38 | 0.37 | 0.45 | 0.23 | 0.29 | 0.39 | 0.20 |
| SALISBURY NHS FOUNDATION TRUST | 1.39 | 2.79 | 0.00 | 3.38 | 5.26 | 2.45 | 0.52 | 1.51 | 0.47 | 0.79 | 0.98 | 0.73 |
| SANDWELL AND WEST BIRMINGHAM HOSPITALS NHS TRUST | 0.52 | 1.04 | 0.00 | 0.07 | 0.99 | 0.04 | 0.05 | 0.05 | 0.04 | 0.11 | 0.21 | 0.08 |
| SHEFFIELD CHILDREN'S NHS FOUNDATION TRUST | 1.03 | 2.02 | 0.05 | 0.66 | 1.32 | 0.47 | 0.02 | 0.10 | 0.02 | 0.04 | 0.05 | 0.03 |
| SHEFFIELD TEACHING HOSPITALS NHS FOUNDATION TRUST | 0.41 | 0.82 | 0.00 | 1.94 | 2.32 | 1.44 | 1.49 | 1.62 | 1.34 | 2.08 | 2.49 | 1.37 |
| SHERWOOD FOREST HOSPITALS NHS FOUNDATION TRUST | 0.31 | 0.63 | 0.00 | 3.82 | 4.14 | 3.09 | 3.28 | 3.50 | 2.87 | 3.09 | 3.50 | 2.05 |
| SHREWSBURY AND TELFORD HOSPITAL NHS TRUST | 1.37 | 2.74 | 0.01 | 1.40 | 2.23 | 0.83 | 0.98 | 1.07 | 0.90 | 1.11 | 1.67 | 0.90 |
| SOMERSET NHS FOUNDATION TRUST | 1.09 | 2.10 | 0.07 | 1.00 | 1.58 | 0.75 | 0.46 | 0.73 | 0.31 | 0.27 | 0.31 | 0.19 |
| SOUTH TEES HOSPITALS NHS FOUNDATION TRUST | 2.04 | 4.07 | 0.00 | 0.68 | 5.36 | 0.43 | 0.25 | 0.41 | 0.21 | 0.22 | 0.35 | 0.17 |
| SOUTH TYNESIDE AND SUNDERLAND NHS FOUNDATION TRUST | 0.42 | 0.84 | 0.00 | 0.47 | 0.91 | 0.37 | 0.23 | 0.30 | 0.20 | 0.26 | 0.46 | 0.17 |
| SOUTH WARWICKSHIRE NHS FOUNDATION TRUST | 0.83 | 1.66 | 0.00 | 1.46 | 1.60 | 0.88 | 0.82 | 1.02 | 0.73 | 0.63 | 0.95 | 0.29 |
| SOUTHPORT AND ORMSKIRK HOSPITAL NHS TRUST | 0.64 | 1.28 | 0.00 | 0.47 | 0.95 | 0.14 | 0.08 | 0.09 | 0.08 | 0.14 | 0.25 | 0.12 |
| ST GEORGE'S UNIVERSITY HOSPITALS NHS FOUNDATION TRUST | 0.52 | 1.04 | 0.00 | 0.38 | 1.11 | 0.23 | 0.07 | 0.20 | 0.07 | 0.21 | 0.28 | 0.16 |
| ST HELENS AND KNOWSLEY TEACHING HOSPITALS NHS TRUST | 0.20 | 0.39 | 0.00 | 0.05 | 0.58 | 0.03 | 0.06 | 0.07 | 0.06 | 0.11 | 0.21 | 0.10 |
| STOCKPORT NHS FOUNDATION TRUST | 1.69 | 3.37 | 0.00 | 2.25 | 3.22 | 1.77 | 1.25 | 1.53 | 1.10 | 0.95 | 1.34 | 0.41 |
| SURREY AND SUSSEX HEALTHCARE NHS TRUST | 0.94 | 1.87 | 0.00 | 3.32 | 4.78 | 1.95 | 2.86 | 3.23 | 1.99 | 3.34 | 4.18 | 3.12 |
| SUSSEX COMMUNITY NHS FOUNDATION TRUST | 0.00 | 0.00 | 0.00 | 0.00 | 0.00 | 0.00 | 0.00 | 0.00 | 0.00 | 0.01 | 0.01 | 0.01 |
| TAMESIDE AND GLOSSOP INTEGRATED CARE NHS FOUNDATION TRUST | 0.44 | 0.88 | 0.00 | 1.04 | 1.24 | 0.94 | 1.07 | 1.27 | 0.95 | 1.54 | 2.33 | 1.28 |
| THE CLATTERBRIDGE CANCER CENTRE NHS FOUNDATION TRUST | 1.65 | 3.27 | 0.04 | 0.10 | 3.22 | 0.00 | 0.15 | 0.15 | 0.01 | 0.15 | 0.24 | 0.13 |
| THE DUDLEY GROUP NHS FOUNDATION TRUST | 1.29 | 2.57 | 0.01 | 0.43 | 2.78 | 0.33 | 0.27 | 0.28 | 0.24 | 0.41 | 0.67 | 0.32 |
| THE HILLINGDON HOSPITALS NHS FOUNDATION TRUST | 1.20 | 2.26 | 0.14 | 1.13 | 2.13 | 0.64 | 0.57 | 0.98 | 0.18 | 0.50 | 0.73 | 0.33 |
| THE NEWCASTLE UPON TYNE HOSPITALS NHS FOUNDATION TRUST | 0.22 | 0.44 | 0.00 | 0.58 | 1.31 | 0.43 | 0.42 | 0.47 | 0.39 | 0.48 | 0.81 | 0.35 |
| THE PRINCESS ALEXANDRA HOSPITAL NHS TRUST | 0.29 | 0.55 | 0.03 | 0.83 | 0.99 | 0.49 | 0.31 | 0.49 | 0.25 | 0.48 | 0.77 | 0.37 |
| THE QUEEN ELIZABETH HOSPITAL, KING'S LYNN, NHS FOUNDATION TRUST | 0.39 | 0.78 | 0.00 | 0.69 | 1.90 | 0.50 | 0.41 | 0.54 | 0.28 | 0.46 | 0.49 | 0.37 |
| THE ROBERT JONES AND AGNES HUNT ORTHOPAEDIC HOSPITAL NHS FOUNDATION TRUST | 0.00 | 0.00 | 0.00 | 1.73 | 2.37 | 0.46 | 0.00 | 0.00 | 0.00 | 0.00 | 0.00 | 0.00 |
| THE ROTHERHAM NHS FOUNDATION TRUST | 0.92 | 1.72 | 0.13 | 1.23 | 1.43 | 0.63 | 0.96 | 1.28 | 0.70 | 0.66 | 0.91 | 0.40 |
| THE ROYAL MARSDEN NHS FOUNDATION TRUST | 0.02 | 0.03 | 0.00 | 0.09 | 0.14 | 0.05 | 0.08 | 0.11 | 0.08 | 0.13 | 0.18 | 0.03 |
| THE ROYAL ORTHOPAEDIC HOSPITAL NHS FOUNDATION TRUST | 0.59 | 1.17 | 0.00 | 0.92 | 1.64 | 0.49 | 0.40 | 0.59 | 0.28 | 0.14 | 0.31 | 0.00 |
| THE ROYAL WOLVERHAMPTON NHS TRUST | 0.31 | 0.62 | 0.01 | 0.16 | 1.08 | 0.07 | 0.06 | 0.07 | 0.06 | 1.06 | 1.58 | 0.07 |
| THE WALTON CENTRE NHS FOUNDATION TRUST | 0.56 | 1.05 | 0.07 | 0.00 | 1.06 | 0.00 | 0.00 | 0.00 | 0.00 | 0.00 | 0.00 | 0.00 |
| TORBAY AND SOUTH DEVON NHS FOUNDATION TRUST | 0.76 | 1.51 | 0.00 | 2.53 | 3.78 | 2.01 | 0.85 | 1.82 | 0.53 | 0.69 | 0.78 | 0.57 |
| UNITED LINCOLNSHIRE HOSPITALS NHS TRUST | 1.82 | 3.63 | 0.00 | 1.54 | 3.80 | 1.07 | 0.95 | 1.22 | 0.82 | 0.66 | 0.94 | 0.46 |
| UNIVERSITY COLLEGE LONDON HOSPITALS NHS FOUNDATION TRUST | 0.15 | 0.30 | 0.00 | 0.07 | 0.59 | 0.06 | 0.02 | 0.03 | 0.02 | 0.06 | 0.08 | 0.04 |
| UNIVERSITY HOSPITAL SOUTHAMPTON NHS FOUNDATION TRUST | 0.23 | 0.46 | 0.00 | 1.41 | 1.54 | 0.78 | 1.28 | 1.45 | 1.14 | 0.97 | 1.34 | 0.61 |
| UNIVERSITY HOSPITALS BIRMINGHAM NHS FOUNDATION TRUST | 0.96 | 1.83 | 0.09 | 0.92 | 1.62 | 0.61 | 0.49 | 0.58 | 0.37 | 0.46 | 0.66 | 0.30 |
| UNIVERSITY HOSPITALS BRISTOL AND WESTON NHS FOUNDATION TRUST | 1.11 | 2.21 | 0.01 | 1.11 | 1.93 | 0.39 | 0.54 | 0.65 | 0.42 | 0.97 | 1.24 | 0.85 |
| UNIVERSITY HOSPITALS COVENTRY AND WARWICKSHIRE NHS TRUST | 0.70 | 1.40 | 0.00 | 0.35 | 1.65 | 0.25 | 0.13 | 0.22 | 0.08 | 0.14 | 0.23 | 0.10 |
| UNIVERSITY HOSPITALS DORSET NHS FOUNDATION TRUST | 0.51 | 1.02 | 0.00 | 1.44 | 2.25 | 1.13 | 0.90 | 1.11 | 0.86 | 1.17 | 1.28 | 0.88 |
| UNIVERSITY HOSPITALS OF DERBY AND BURTON NHS FOUNDATION TRUST | 1.69 | 3.09 | 0.29 | 1.59 | 2.50 | 1.09 | 0.37 | 0.49 | 0.32 | 0.43 | 0.60 | 0.32 |
| UNIVERSITY HOSPITALS OF LEICESTER NHS TRUST | 0.47 | 0.94 | 0.00 | 0.52 | 1.24 | 0.39 | 0.57 | 0.58 | 0.57 | 0.94 | 1.31 | 0.81 |
| UNIVERSITY HOSPITALS OF MORECAMBE BAY NHS FOUNDATION TRUST | 0.13 | 0.27 | 0.00 | 0.28 | 0.39 | 0.20 | 0.13 | 0.16 | 0.12 | 0.14 | 0.20 | 0.12 |
| UNIVERSITY HOSPITALS OF NORTH MIDLANDS NHS TRUST | 0.29 | 0.59 | 0.00 | 1.00 | 1.32 | 0.88 | 0.30 | 0.52 | 0.28 | 0.40 | 0.60 | 0.31 |
| UNIVERSITY HOSPITALS PLYMOUTH NHS TRUST | 0.61 | 1.17 | 0.04 | 0.41 | 0.76 | 0.27 | 0.30 | 0.33 | 0.29 | 0.50 | 0.55 | 0.33 |
| WALSALL HEALTHCARE NHS TRUST | 1.39 | 2.71 | 0.07 | 0.11 | 1.98 | 0.02 | 0.05 | 0.05 | 0.05 | 0.16 | 0.25 | 0.08 |
| WARRINGTON AND HALTON TEACHING HOSPITALS NHS FOUNDATION TRUST | 1.21 | 2.22 | 0.20 | 0.60 | 1.72 | 0.02 | 0.04 | 0.05 | 0.03 | 0.12 | 0.20 | 0.09 |
| WEST HERTFORDSHIRE HOSPITALS NHS TRUST | 0.00 | 0.00 | 0.00 | 0.89 | 1.27 | 0.00 | 1.05 | 1.24 | 0.71 | 0.70 | 0.98 | 0.61 |
| WEST SUFFOLK NHS FOUNDATION TRUST | 1.13 | 2.25 | 0.00 | 1.77 | 3.90 | 0.90 | 0.50 | 0.70 | 0.41 | 0.53 | 0.63 | 0.37 |
| WESTERN SUSSEX HOSPITALS NHS FOUNDATION TRUST | 1.33 | 2.66 | 0.00 | 1.33 | 2.38 | 0.88 | 0.77 | 0.96 | 0.52 | 0.77 | 0.90 | 0.62 |
| WHITTINGTON HEALTH NHS TRUST | 0.44 | 0.88 | 0.00 | 0.96 | 1.53 | 0.44 | 0.65 | 0.91 | 0.61 | 0.68 | 1.18 | 0.40 |
| WIRRAL UNIVERSITY TEACHING HOSPITAL NHS FOUNDATION TRUST | 0.89 | 1.77 | 0.00 | 0.18 | 1.66 | 0.03 | 0.05 | 0.06 | 0.04 | 0.17 | 0.28 | 0.11 |
| WORCESTERSHIRE ACUTE HOSPITALS NHS TRUST | 1.38 | 2.75 | 0.00 | 1.77 | 2.62 | 0.87 | 1.27 | 1.34 | 1.15 | 1.01 | 1.19 | 0.82 |
| WRIGHTINGTON, WIGAN AND LEIGH NHS FOUNDATION TRUST | 0.95 | 1.90 | 0.00 | 0.60 | 1.39 | 0.34 | 0.31 | 0.37 | 0.29 | 0.34 | 0.50 | 0.27 |
| WYE VALLEY NHS TRUST | 0.69 | 1.36 | 0.02 | 1.46 | 2.57 | 1.27 | 0.80 | 0.96 | 0.68 | 0.64 | 1.00 | 0.47 |
| YEOVIL DISTRICT HOSPITAL NHS FOUNDATION TRUST | 0.00 | 0.00 | 0.00 | 1.28 | 1.52 | 0.46 | 1.01 | 1.35 | 0.74 | 0.83 | 1.17 | 0.41 |
| YORK TEACHING HOSPITAL NHS FOUNDATION TRUST | 0.56 | 1.12 | 0.00 | 1.43 | 2.35 | 0.47 | 0.19 | 0.43 | 0.07 | 0.49 | 0.59 | 0.12 |

## Table S4. Summary of the numbers of nosocomial infections in 136 NHS trusts during the entire evaluation period, obtained from the ISARIC data.

|  | **Trust name** | **Number of nosocomial infections using 7 days as a cut off** | **Number of nosocomial infections using 14 days as a cut off** |
| --- | --- | --- | --- |
| 1 | AINTREE UNIVERSITY HOSPITAL NHS FOUNDATION TRUST | 149 | 93 |
| 2 | AIREDALE NHS FOUNDATION TRUST | 10 | 9 |
| 3 | ALDER HEY CHILDREN'S NHS FOUNDATION TRUST | 4 | 4 |
| 4 | ASHFORD AND ST PETER'S HOSPITALS NHS FOUNDATION TRUST | 42 | 25 |
| 5 | BARNSLEY HOSPITAL NHS FOUNDATION TRUST | 23 | 15 |
| 6 | BARTS HEALTH NHS TRUST | 218 | 70 |
| 7 | BASILDON AND THURROCK UNIVERSITY HOSPITALS NHS FOUNDATION TRUST | 72 | 39 |
| 8 | BASINGSTOKE AND NORTH HAMPSHIRE NHS FOUNDATION TRUST | 80 | 44 |
| 9 | BEDFORDSHIRE HOSPITALS NHS FOUNDATION TRUST | 27 | 8 |
| 10 | BIRMINGHAM CHILDREN'S HOSPITAL NHS FOUNDATION TRUST | 6 | 6 |
| 11 | BLACKPOOL TEACHING HOSPITALS NHS FOUNDATION TRUST | 2 | 1 |
| 12 | BOLTON NHS FOUNDATION TRUST | 32 | 21 |
| 13 | BRADFORD TEACHING HOSPITALS NHS FOUNDATION TRUST | 33 | 24 |
| 14 | BRIGHTON AND SUSSEX UNIVERSITY HOSPITALS NHS TRUST | 26 | 12 |
| 15 | BUCKINGHAMSHIRE HEALTHCARE NHS TRUST | 53 | 40 |
| 16 | CALDERDALE AND HUDDERSFIELD NHS FOUNDATION TRUST | 23 | 16 |
| 17 | CAMBRIDGE UNIVERSITY HOSPITALS NHS FOUNDATION TRUST | 21 | 15 |
| 18 | CHELSEA AND WESTMINSTER HOSPITAL NHS FOUNDATION TRUST | 5 | 3 |
| 19 | CHESTERFIELD ROYAL HOSPITAL NHS FOUNDATION TRUST | 21 | 12 |
| 20 | CHRISTIE NHS FOUNDATION TRUST | 9 | 6 |
| 21 | CLATTERBRIDGE CANCER CENTRE NHS FOUNDATION TRUST | 0 | 0 |
| 22 | COLCHESTER HOSPITAL UNIVERSITY NHS FOUNDATION TRUST | 70 | 31 |
| 23 | COUNTESS OF CHESTER HOSPITAL NHS FOUNDATION TRUST | 122 | 62 |
| 24 | COUNTY DURHAM AND DARLINGTON NHS FOUNDATION TRUST | 22 | 7 |
| 25 | CROYDON HEALTH SERVICES NHS TRUST | 2 | 2 |
| 26 | DARTFORD AND GRAVESHAM NHS TRUST | 2 | 2 |
| 27 | DERBY HOSPITALS NHS FOUNDATION TRUST | 73 | 30 |
| 28 | DONCASTER AND BASSETLAW HOSPITALS NHS FOUNDATION TRUST | 43 | 24 |
| 29 | DORSET COUNTY HOSPITAL NHS FOUNDATION TRUST | 14 | 10 |
| 30 | DUDLEY GROUP NHS FOUNDATION TRUST | 29 | 14 |
| 31 | EAST AND NORTH HERTFORDSHIRE NHS TRUST | 2 | 1 |
| 32 | EAST CHESHIRE NHS TRUST | 32 | 17 |
| 33 | EAST KENT HOSPITALS UNIVERSITY NHS FOUNDATION TRUST | 52 | 25 |
| 34 | EAST LANCASHIRE HOSPITALS NHS TRUST | 91 | 64 |
| 35 | EAST SUSSEX HEALTHCARE NHS TRUST | 77 | 40 |
| 36 | EPSOM AND ST HELIER UNIVERSITY HOSPITALS NHS TRUST | 5 | 5 |
| 37 | FRIMLEY HEALTH NHS FOUNDATION TRUST | 9 | 6 |
| 38 | GATESHEAD HEALTH NHS FOUNDATION TRUST | 24 | 11 |
| 39 | GEORGE ELIOT HOSPITAL NHS TRUST | 17 | 13 |
| 40 | GLOUCESTERSHIRE HOSPITALS NHS FOUNDATION TRUST | 17 | 11 |
| 41 | GREAT ORMOND STREET HOSPITAL FOR CHILDREN NHS FOUNDATION TRUST | 0 | 0 |
| 42 | GREAT WESTERN HOSPITALS NHS FOUNDATION TRUST | 2 | 2 |
| 43 | GUY'S AND ST THOMAS' NHS FOUNDATION TRUST | 12 | 10 |
| 44 | HARROGATE AND DISTRICT NHS FOUNDATION TRUST | 30 | 18 |
| 45 | HEREFORD HOSPITALS NHS TRUST | 9 | 4 |
| 46 | HILLINGDON HOSPITAL NHS TRUST | 8 | 5 |
| 47 | HOMERTON UNIVERSITY HOSPITAL NHS FOUNDATION TRUST | 4 | 4 |
| 48 | HULL UNIVERSITY TEACHING HOSPITALS NHS TRUST | 0 | 0 |
| 49 | IMPERIAL COLLEGE HEALTHCARE NHS TRUST | 5 | 3 |
| 50 | ISLE OF WIGHT NHS TRUST | 5 | 2 |
| 51 | JAMES PAGET UNIVERSITY HOSPITALS NHS FOUNDATION TRUST | 5 | 2 |
| 52 | KETTERING GENERAL HOSPITAL NHS FOUNDATION TRUST | 9 | 3 |
| 53 | KING'S COLLEGE HOSPITAL NHS FOUNDATION TRUST | 2 | 1 |
| 54 | KINGSTON HOSPITAL NHS FOUNDATION TRUST | 10 | 7 |
| 55 | LANCASHIRE TEACHING HOSPITALS NHS FOUNDATION TRUST | 42 | 23 |
| 56 | LEEDS TEACHING HOSPITALS NHS TRUST | 31 | 26 |
| 57 | LEWISHAM HEALTHCARE NHS TRUST | 0 | 0 |
| 58 | LIVERPOOL HEART AND CHEST HOSPITAL NHS FOUNDATION TRUST | 9 | 3 |
| 59 | LIVERPOOL WOMEN'S NHS FOUNDATION TRUST | 0 | 0 |
| 60 | LONDON NORTH WEST HEALTHCARE NHS TRUST | 10 | 1 |
| 61 | MAIDSTONE AND TUNBRIDGE WELLS NHS TRUST | 3 | 2 |
| 62 | MANCHESTER UNIVERSITY NHS FOUNDATION TRUST | 80 | 47 |
| 63 | MID AND SOUTH ESSEX NHS FOUNDATION TRUST | 23 | 14 |
| 64 | MID CHESHIRE HOSPITALS NHS FOUNDATION TRUST | 12 | 9 |
| 65 | MID YORKSHIRE HOSPITALS NHS TRUST | 31 | 16 |
| 66 | MILTON KEYNES HOSPITAL NHS FOUNDATION TRUST | 32 | 13 |
| 67 | NEWCASTLE UPON TYNE HOSPITALS NHS FOUNDATION TRUST | 25 | 18 |
| 68 | NORFOLK AND NORWICH UNIVERSITY HOSPITALS NHS FOUNDATION TRUST | 1 | 0 |
| 69 | NORTH BRISTOL NHS TRUST | 13 | 11 |
| 70 | NORTH CUMBRIA INTEGRATED CARE NHS FOUNDATION TRUST | 10 | 3 |
| 71 | NORTH MIDDLESEX UNIVERSITY HOSPITAL NHS TRUST | 6 | 4 |
| 72 | NORTH TEES AND HARTLEPOOL NHS FOUNDATION TRUST | 4 | 1 |
| 73 | NORTH WEST ANGLIA NHS FOUNDATION TRUST | 30 | 15 |
| 74 | NORTHAMPTON GENERAL HOSPITAL NHS TRUST | 48 | 27 |
| 75 | NORTHERN DEVON HEALTHCARE NHS TRUST | 18 | 7 |
| 76 | NORTHERN LINCOLNSHIRE AND GOOLE HOSPITALS NHS FOUNDATION TRUST | 9 | 5 |
| 77 | NORTHUMBRIA HEALTHCARE NHS FOUNDATION TRUST | 0 | 0 |
| 78 | NOTTINGHAM UNIVERSITY HOSPITALS NHS TRUST | 60 | 23 |
| 79 | OXFORD RADCLIFFE HOSPITALS NHS TRUST | 13 | 9 |
| 80 | PAPWORTH HOSPITAL NHS FOUNDATION TRUST | 1 | 1 |
| 81 | PENNINE ACUTE HOSPITALS NHS TRUST | 67 | 40 |
| 82 | PLYMOUTH HOSPITALS NHS TRUST | 10 | 7 |
| 83 | POOLE HOSPITAL NHS FOUNDATION TRUST | 58 | 29 |
| 84 | PORTSMOUTH HOSPITALS NHS TRUST | 93 | 42 |
| 85 | PRINCESS ALEXANDRA HOSPITAL NHS TRUST | 0 | 0 |
| 86 | QUEEN ELIZABETH HOSPITAL KING'S LYNN NHS FOUNDATION TRUST | 104 | 46 |
| 87 | ROTHERHAM NHS FOUNDATION TRUST | 41 | 27 |
| 88 | ROYAL BERKSHIRE NHS FOUNDATION TRUST | 41 | 24 |
| 89 | ROYAL BOURNEMOUTH AND CHRISTCHURCH HOSPITALS NHS FOUNDATION TRUST | 2 | 1 |
| 90 | ROYAL BROMPTON AND HAREFIELD NHS FOUNDATION TRUST | 4 | 2 |
| 91 | ROYAL CORNWALL HOSPITALS NHS TRUST | 8 | 4 |
| 92 | ROYAL DEVON AND EXETER NHS FOUNDATION TRUST | 12 | 9 |
| 93 | ROYAL FREE HAMPSTEAD NHS TRUST | 0 | 0 |
| 94 | ROYAL MARSDEN NHS FOUNDATION TRUST | 2 | 1 |
| 95 | ROYAL ORTHOPAEDIC HOSPITAL NHS FOUNDATION TRUST | 0 | 0 |
| 96 | ROYAL SURREY COUNTY HOSPITAL NHS FOUNDATION TRUST | 8 | 5 |
| 97 | ROYAL UNITED HOSPITAL BATH NHS TRUST | 34 | 18 |
| 98 | ROYAL WOLVERHAMPTON HOSPITALS NHS TRUST | 21 | 12 |
| 99 | SALFORD ROYAL NHS FOUNDATION TRUST | 54 | 31 |
| 100 | SALISBURY NHS FOUNDATION TRUST | 0 | 0 |
| 101 | SANDWELL AND WEST BIRMINGHAM HOSPITALS NHS TRUST | 11 | 5 |
| 102 | SHEFFIELD CHILDREN'S NHS FOUNDATION TRUST | 0 | 0 |
| 103 | SHEFFIELD TEACHING HOSPITALS NHS FOUNDATION TRUST | 5 | 5 |
| 104 | SHERWOOD FOREST HOSPITALS NHS FOUNDATION TRUST | 2 | 1 |
| 105 | SHREWSBURY AND TELFORD HOSPITAL NHS TRUST | 6 | 5 |
| 106 | SOMERSET NHS FOUNDATION TRUST | 71 | 49 |
| 107 | SOUTH DEVON HEALTHCARE NHS FOUNDATION TRUST | 5 | 4 |
| 108 | SOUTH TEES HOSPITALS NHS FOUNDATION TRUST | 3 | 2 |
| 109 | SOUTH TYNESIDE AND SUNDERLAND NHS FOUNDATION TRUST | 17 | 10 |
| 110 | SOUTH WARWICKSHIRE GENERAL HOSPITALS NHS TRUST | 17 | 8 |
| 111 | SOUTHAMPTON UNIVERSITY HOSPITALS NHS TRUST | 10 | 4 |
| 112 | SOUTHPORT AND ORMSKIRK HOSPITAL NHS TRUST | 0 | 0 |
| 113 | ST GEORGE'S HEALTHCARE NHS TRUST | 20 | 12 |
| 114 | ST HELENS AND KNOWSLEY HOSPITALS NHS TRUST | 7 | 5 |
| 115 | STOCKPORT NHS FOUNDATION TRUST | 5 | 5 |
| 116 | SURREY AND SUSSEX HEALTHCARE NHS TRUST | 61 | 31 |
| 117 | TAMESIDE HOSPITAL NHS FOUNDATION TRUST | 3 | 2 |
| 118 | UNITED LINCOLNSHIRE HOSPITALS NHS TRUST | 285 | 121 |
| 119 | UNIVERSITY HOSPITAL OF NORTH STAFFORDSHIRE NHS TRUST | 41 | 26 |
| 120 | UNIVERSITY HOSPITALS BIRMINGHAM NHS FOUNDATION TRUST | 87 | 43 |
| 121 | UNIVERSITY HOSPITALS BRISTOL NHS FOUNDATION TRUST | 39 | 23 |
| 122 | UNIVERSITY HOSPITALS COVENTRY AND WARWICKSHIRE NHS TRUST | 62 | 45 |
| 123 | UNIVERSITY HOSPITALS OF LEICESTER NHS TRUST | 36 | 17 |
| 124 | UNIVERSITY HOSPITALS OF MORECAMBE BAY NHS FOUNDATION TRUST | 7 | 6 |
| 125 | WALSALL HEALTHCARE NHS TRUST | 3 | 2 |
| 126 | WALTON CENTRE NHS FOUNDATION TRUST | 0 | 0 |
| 127 | WARRINGTON AND HALTON HOSPITALS NHS FOUNDATION TRUST | 4 | 2 |
| 128 | WEST HERTFORDSHIRE HOSPITALS NHS TRUST | 40 | 29 |
| 129 | WEST SUFFOLK HOSPITALS NHS TRUST | 11 | 4 |
| 130 | WESTERN SUSSEX HOSPITALS NHS FOUNDATION TRUST | 13 | 8 |
| 131 | WHITTINGTON HEALTH NHS TRUST | 6 | 3 |
| 132 | WIRRAL UNIVERSITY TEACHING HOSPITAL NHS FOUNDATION TRUST | 45 | 26 |
| 133 | WORCESTERSHIRE ACUTE HOSPITALS NHS TRUST | 19 | 12 |
| 134 | WRIGHTINGTON, WIGAN AND LEIGH NHS FOUNDATION TRUST | 63 | 24 |
| 135 | YEOVIL DISTRICT HOSPITAL NHS FOUNDATION TRUST | 2 | 2 |
| 136 | YORK HOSPITALS NHS FOUNDATION TRUST | 58 | 33 |

## Table S5. Summary of hospitalised COVID-19 cases per week and per trust over the four evaluation periods.

| **Trust name** | **November 2020–December 2020** | | | **January 2021–July 2021** | | | **August 2021–November 2021** | | | **December 2021–March 2022** | | |
| --- | --- | --- | --- | --- | --- | --- | --- | --- | --- | --- | --- | --- |
|  |  | | |  | | |  | | |  | | |
|  |  |  |  |  |  |  |  |  |  |  |  |  |
|  | 51 | 121 | 2 | 32.5 | 150 | 1 | 25 | 53 | 9 | 7 | 20 | 1 |
| AIREDALE NHS FOUNDATION TRUST | 12 | 32 | 1 | 4 | 30 | 1 | 10 | 18 | 5 | 9 | 17 | 3 |
| ALDER HEY CHILDREN'S NHS FOUNDATION TRUST | 2 | 8 | 1 | 2 | 10 | 1 | 1.5 | 4 | 1 | 1 | 4 | 1 |
| ASHFORD AND ST PETER'S HOSPITALS NHS FOUNDATION TRUST | 19 | 33 | 4 | 6.5 | 35 | 1 | 7.5 | 21 | 1 | 3 | 10 | 1 |
| BARNSLEY HOSPITAL NHS FOUNDATION TRUST | 32 | 105 | 10 | 9 | 51 | 1 | 7 | 22 | 1 | 3 | 6 | 2 |
| BARTS HEALTH NHS TRUST | 84 | 340 | 2 | 20 | 531 | 1 | 38 | 80 | 1 | 73.5 | 182 | 16 |
| BASILDON AND THURROCK UNIVERSITY HOSPITALS NHS FOUNDATION TRUST | 41 | 109 | 10 | 3 | 29 | 1 | 18 | 39 | 7 | 30 | 62 | 8 |
| BASINGSTOKE AND NORTH HAMPSHIRE NHS FOUNDATION TRUST | 21 | 45 | 4 | 10.5 | 89 | 1 | 18 | 33 | 8 | 24 | 29 | 13 |
| BEDFORDSHIRE HOSPITALS NHS FOUNDATION TRUST | 7 | 29 | 2 | 3.5 | 83 | 1 | 18.5 | 42 | 2 | 30 | 37 | 20 |
| BIRMINGHAM CHILDREN'S HOSPITAL NHS FOUNDATION TRUST | 3 | 10 | 1 | 3 | 13 | 1 | 4 | 7 | 1 | 10 | 35 | 4 |
| BLACKPOOL TEACHING HOSPITALS NHS FOUNDATION TRUST | NA | NA | NA | 3 | 19 | 1 | 6 | 13 | 1 | 1 | 1 | 1 |
| BOLTON NHS FOUNDATION TRUST | 30 | 69 | 9 | 12 | 33 | 1 | 17 | 25 | 12 | 19 | 38 | 1 |
| BRADFORD TEACHING HOSPITALS NHS FOUNDATION TRUST | 22.5 | 51 | 1 | 8 | 53 | 1 | 22 | 33 | 7 | 30 | 48 | 6 |
| BRIGHTON AND SUSSEX UNIVERSITY HOSPITALS NHS TRUST | 11.5 | 37 | 5 | 3 | 39 | 1 | 8 | 19 | 1 | 5 | 8 | 2 |
| BUCKINGHAMSHIRE HEALTHCARE NHS TRUST | 18 | 45 | 5 | 10.5 | 80 | 1 | 6 | 11 | 4 | NA | NA | NA |
| CALDERDALE AND HUDDERSFIELD NHS FOUNDATION TRUST | 26 | 55 | 5 | 7 | 45 | 1 | 26 | 40 | 10 | 18 | 37 | 2 |
| CAMBRIDGE UNIVERSITY HOSPITALS NHS FOUNDATION TRUST | 13 | 35 | 1 | 54 | 101 | 1 | 4 | 4 | 4 | NA | NA | NA |
| CHELSEA AND WESTMINSTER HOSPITAL NHS FOUNDATION TRUST | 1 | 1 | 1 | 2 | 17 | 1 | NA | NA | NA | NA | NA | NA |
| CHESTERFIELD ROYAL HOSPITAL NHS FOUNDATION TRUST | 17.5 | 43 | 1 | 7 | 65 | 1 | 1 | 12 | 1 | 6 | 17 | 1 |
| CHRISTIE NHS FOUNDATION TRUST | 1.5 | 4 | 1 | 2 | 4 | 1 | 2 | 4 | 1 | 1 | 1 | 1 |
| CLATTERBRIDGE CANCER CENTRE NHS FOUNDATION TRUST | 1 | 3 | 1 | 2 | 3 | 1 | 1 | 1 | 1 | 1 | 3 | 1 |
| COLCHESTER HOSPITAL UNIVERSITY NHS FOUNDATION TRUST | 35 | 96 | 7 | 6 | 216 | 1 | 24 | 45 | 13 | 18.5 | 33 | 2 |
| COUNTESS OF CHESTER HOSPITAL NHS FOUNDATION TRUST | 31 | 42 | 3 | 8 | 58 | 1 | 11 | 25 | 5 | 16 | 37 | 1 |
| COUNTY DURHAM AND DARLINGTON NHS FOUNDATION TRUST | 35.5 | 49 | 11 | 11 | 54 | 1 | 23 | 34 | 3 | 18 | 22 | 2 |
| CROYDON HEALTH SERVICES NHS TRUST | 1 | 7 | 1 | NA | NA | NA | NA | NA | NA | NA | NA | NA |
| DARTFORD AND GRAVESHAM NHS TRUST | 4 | 10 | 1 | 2 | 7 | 1 | 5.5 | 7 | 1 | 1 | 1 | 1 |
| DERBY HOSPITALS NHS FOUNDATION TRUST | 9 | 85 | 1 | 5 | 41 | 1 | 6 | 34 | 1 | 15.5 | 30 | 3 |
| DONCASTER AND BASSETLAW HOSPITALS NHS FOUNDATION TRUST | 27.5 | 61 | 11 | 5 | 26 | 1 | 12 | 18 | 1 | NA | NA | NA |
| DORSET COUNTY HOSPITAL NHS FOUNDATION TRUST | 4 | 9 | 1 | 3 | 37 | 1 | 9 | 17 | 2 | 6 | 13 | 1 |
| DUDLEY GROUP NHS FOUNDATION TRUST | 30 | 74 | 6 | 33 | 48 | 1 | 17 | 28 | 2 | 19 | 48 | 5 |
| EAST AND NORTH HERTFORDSHIRE NHS TRUST | 7 | 11 | 3 | 2 | 13 | 1 | 2 | 4 | 1 | NA | NA | NA |
| EAST CHESHIRE NHS TRUST | 11.5 | 16 | 6 | 5 | 24 | 1 | 9 | 18 | 3 | 3 | 7 | 1 |
| EAST KENT HOSPITALS UNIVERSITY NHS FOUNDATION TRUST | 31 | 98 | 6 | 4 | 55 | 1 | NA | NA | NA | NA | NA | NA |
| EAST LANCASHIRE HOSPITALS NHS TRUST | 56 | 84 | 3 | 24 | 139 | 2 | 30 | 50 | 20 | 28 | 83 | 2 |
| EAST SUSSEX HEALTHCARE NHS TRUST | 19 | 88 | 5 | 6 | 161 | 1 | 12 | 37 | 4 | 12 | 21 | 5 |
| EPSOM AND ST HELIER UNIVERSITY HOSPITALS NHS TRUST | 3 | 3 | 3 | 5.5 | 45 | 1 | 3 | 3 | 1 | 12 | 14 | 2 |
| FRIMLEY HEALTH NHS FOUNDATION TRUST | 6 | 21 | 1 | 5 | 15 | 1 | 9 | 17 | 1 | NA | NA | NA |
| GATESHEAD HEALTH NHS FOUNDATION TRUST | 29 | 67 | 7 | 3 | 64 | 1 | 16.5 | 27 | 8 | NA | NA | NA |
| GEORGE ELIOT HOSPITAL NHS TRUST | 16 | 40 | 4 | 6 | 45 | 1 | 12 | 20 | 3 | 15 | 32 | 2 |
| GLOUCESTERSHIRE HOSPITALS NHS FOUNDATION TRUST | 8 | 34 | 1 | 5 | 20 | 1 | 13 | 25 | 1 | 7 | 7 | 7 |
| GREAT ORMOND STREET HOSPITAL FOR CHILDREN NHS FOUNDATION TRUST | 1 | 3 | 1 | 2 | 5 | 1 | 1 | 2 | 1 | NA | NA | NA |
| GREAT WESTERN HOSPITALS NHS FOUNDATION TRUST | 7 | 15 | 2 | 2 | 8 | 1 | 1 | 1 | 1 | NA | NA | NA |
| GUY'S AND ST THOMAS' NHS FOUNDATION TRUST | 10 | 99 | 2 | 3 | 126 | 1 | NA | NA | NA | 2 | 10 | 1 |
| HARROGATE AND DISTRICT NHS FOUNDATION TRUST | 8 | 22 | 1 | 4 | 26 | 1 | 3.5 | 9 | 1 | 7 | 13 | 1 |
| HEREFORD HOSPITALS NHS TRUST | 17 | 25 | 3 | 5 | 52 | 1 | 11 | 21 | 5 | NA | NA | NA |
| HILLINGDON HOSPITAL NHS TRUST | 22 | 48 | 1 | 10 | 53 | 1 | 9 | 21 | 1 | 13.5 | 26 | 1 |
| HOMERTON UNIVERSITY HOSPITAL NHS FOUNDATION TRUST | 11 | 113 | 1 | 4 | 168 | 1 | 11 | 26 | 5 | 15 | 37 | 5 |
| HULL UNIVERSITY TEACHING HOSPITALS NHS TRUST | 3 | 3 | 2 | 3 | 22 | 1 | NA | NA | NA | NA | NA | NA |
| IMPERIAL COLLEGE HEALTHCARE NHS TRUST | 2 | 16 | 1 | 4.5 | 35 | 1 | 1.5 | 3 | 1 | NA | NA | NA |
| ISLE OF WIGHT NHS TRUST | 3 | 19 | 1 | 4.5 | 52 | 1 | 8 | 19 | 2 | NA | NA | NA |
| JAMES PAGET UNIVERSITY HOSPITALS NHS FOUNDATION TRUST | 10.5 | 19 | 1 | 6 | 53 | 1 | 13 | 23 | 8 | 10 | 13 | 6 |
| KETTERING GENERAL HOSPITAL NHS FOUNDATION TRUST | 8.5 | 20 | 4 | 5.5 | 31 | 1 | 5.5 | 27 | 1 | 4 | 8 | 1 |
| KING'S COLLEGE HOSPITAL NHS FOUNDATION TRUST | 3 | 8 | 1 | 2 | 4 | 1 | NA | NA | NA | NA | NA | NA |
| KINGSTON HOSPITAL NHS FOUNDATION TRUST | NA | NA | NA | 2 | 10 | 1 | 4 | 8 | 1 | 4 | 6 | 1 |
| LANCASHIRE TEACHING HOSPITALS NHS FOUNDATION TRUST | 8 | 27 | 1 | 9 | 48 | 1 | 3 | 11 | 1 | 6 | 13 | 3 |
| LEEDS TEACHING HOSPITALS NHS TRUST | 39 | 82 | 5 | 5 | 59 | 1 | 28 | 56 | 1 | 9.5 | 31 | 1 |
| LEWISHAM HEALTHCARE NHS TRUST | 4.5 | 19 | 1 | 6.5 | 18 | 2 | 2 | 2 | 2 | NA | NA | NA |
| LIVERPOOL HEART AND CHEST HOSPITAL NHS FOUNDATION TRUST | 1.5 | 4 | 1 | 2 | 7 | 2 | NA | NA | NA | NA | NA | NA |
| LIVERPOOL WOMEN'S NHS FOUNDATION TRUST | 1 | 1 | 1 | 1 | 4 | 1 | 1 | 1 | 1 | NA | NA | NA |
| LONDON NORTH WEST HEALTHCARE NHS TRUST | 2.5 | 6 | 1 | 4 | 122 | 1 | 5 | 8 | 1 | 12 | 29 | 1 |
| MAIDSTONE AND TUNBRIDGE WELLS NHS TRUST | 12 | 18 | 1 | 5 | 22 | 1 | 6.5 | 14 | 1 | 1 | 1 | 1 |
| MANCHESTER UNIVERSITY NHS FOUNDATION TRUST | 36 | 77 | 3 | 19 | 109 | 2 | 1 | 5 | 1 | 28 | 89 | 1 |
| MID AND SOUTH ESSEX NHS FOUNDATION TRUST | 8 | 36 | 1 | 4 | 12 | 1 | 12 | 21 | 4 | 8 | 24 | 2 |
| MID CHESHIRE HOSPITALS NHS FOUNDATION TRUST | 10.5 | 14 | 4 | 5.5 | 21 | 1 | 6 | 10 | 1 | NA | NA | NA |
| MID YORKSHIRE HOSPITALS NHS TRUST | 50 | 122 | 1 | 11 | 44 | 1 | 10 | 43 | 1 | 5 | 6 | 4 |
| MILTON KEYNES HOSPITAL NHS FOUNDATION TRUST | 7.5 | 33 | 1 | 2 | 22 | 1 | 9.5 | 22 | 1 | 21 | 27 | 11 |
| NEWCASTLE UPON TYNE HOSPITALS NHS FOUNDATION TRUST | 10.5 | 32 | 1 | 6 | 37 | 1 | 12 | 26 | 3 | 9 | 23 | 2 |
| NORFOLK AND NORWICH UNIVERSITY HOSPITALS NHS FOUNDATION TRUST | 6 | 10 | 4 | 4 | 21 | 1 | 2 | 3 | 1 | NA | NA | NA |
| NORTH BRISTOL NHS TRUST | 21 | 37 | 1 | 9 | 44 | 1 | 20 | 35 | 10 | 9 | 19 | 2 |
| NORTH CUMBRIA INTEGRATED CARE NHS FOUNDATION TRUST | 12 | 17 | 2 | 8 | 64 | 1 | 6 | 12 | 1 | 2 | 3 | 1 |
| NORTH MIDDLESEX UNIVERSITY HOSPITAL NHS TRUST | 27.5 | 64 | 1 | 10 | 132 | 1 | 2 | 30 | 1 | 35.5 | 92 | 2 |
| NORTH TEES AND HARTLEPOOL NHS FOUNDATION TRUST | 12 | 22 | 1 | 3.5 | 11 | 1 | 3 | 8 | 1 | 4 | 8 | 3 |
| NORTH WEST ANGLIA NHS FOUNDATION TRUST | 24 | 30 | 1 | 9 | 44 | 1 | 2 | 4 | 1 | 4 | 4 | 4 |
| NORTHAMPTON GENERAL HOSPITAL NHS TRUST | 19 | 32 | 6 | 4 | 115 | 1 | 21 | 38 | 7 | 25 | 45 | 16 |
| NORTHERN DEVON HEALTHCARE NHS TRUST | 8 | 19 | 1 | 3 | 10 | 1 | 10 | 26 | 4 | 10 | 16 | 4 |
| NORTHERN LINCOLNSHIRE AND GOOLE HOSPITALS NHS FOUNDATION TRUST | 4.5 | 24 | 1 | 4 | 31 | 1 | 9.5 | 21 | 1 | 5 | 10 | 1 |
| NORTHUMBRIA HEALTHCARE NHS FOUNDATION TRUST | 1 | 5 | 1 | 2 | 5 | 1 | NA | NA | NA | NA | NA | NA |
| NOTTINGHAM UNIVERSITY HOSPITALS NHS TRUST | 62 | 99 | 3 | 11 | 113 | 2 | 26 | 45 | 10 | 14 | 98 | 2 |
| OXFORD RADCLIFFE HOSPITALS NHS TRUST | 3 | 15 | 1 | 10 | 55 | 1 | 10 | 23 | 1 | 6 | 9 | 2 |
| PAPWORTH HOSPITAL NHS FOUNDATION TRUST | 3.5 | 7 | 1 | 2 | 24 | 1 | 2 | 5 | 1 | NA | NA | NA |
| PENNINE ACUTE HOSPITALS NHS TRUST | 56 | 134 | 3 | 10 | 69 | 2 | 10 | 44 | 4 | 13 | 22 | 5 |
| PLYMOUTH HOSPITALS NHS TRUST | 2 | 3 | 1 | 6.5 | 31 | 1 | 13 | 25 | 2 | 14.5 | 34 | 3 |
| POOLE HOSPITAL NHS FOUNDATION TRUST | 14.5 | 29 | 4 | 5 | 82 | 1 | 12 | 26 | 2 | 8.5 | 18 | 4 |
| PORTSMOUTH HOSPITALS NHS TRUST | 55 | 134 | 4 | 11.5 | 243 | 1 | 50 | 66 | 24 | 67 | 103 | 10 |
| PRINCESS ALEXANDRA HOSPITAL NHS TRUST | 2.5 | 4 | 1 | 2 | 3 | 1 | 2 | 5 | 1 | 4.5 | 18 | 2 |
| QUEEN ELIZABETH HOSPITAL KING'S LYNN NHS FOUNDATION TRUST | 19 | 56 | 4 | 3 | 97 | 1 | 12 | 26 | 1 | 13 | 27 | 2 |
| ROTHERHAM NHS FOUNDATION TRUST | 49.5 | 82 | 6 | 9 | 42 | 1 | 28 | 41 | 10 | 21 | 56 | 2 |
| ROYAL BERKSHIRE NHS FOUNDATION TRUST | 25.5 | 79 | 4 | 4 | 121 | 1 | 13.5 | 27 | 1 | 17 | 31 | 4 |
| ROYAL BOURNEMOUTH AND CHRISTCHURCH HOSPITALS NHS FOUNDATION TRUST | 6.5 | 14 | 1 | 7.5 | 29 | 1 | 7.5 | 13 | 2 | 3 | 7 | 1 |
| ROYAL BROMPTON AND HAREFIELD NHS FOUNDATION TRUST | 2 | 4 | 1 | 2 | 8 | 1 | 1 | 2 | 1 | NA | NA | NA |
| ROYAL CORNWALL HOSPITALS NHS TRUST | 2 | 6 | 1 | 4 | 18 | 1 | 14 | 30 | 5 | 2 | 2 | 2 |
| ROYAL DEVON AND EXETER NHS FOUNDATION TRUST | 17.5 | 32 | 1 | 3 | 22 | 1 | 6.5 | 22 | 1 | NA | NA | NA |
| ROYAL FREE HAMPSTEAD NHS TRUST | NA | NA | NA | 4 | 16 | 1 | 2 | 5 | 1 | 1 | 9 | 1 |
| ROYAL MARSDEN NHS FOUNDATION TRUST | 1 | 3 | 1 | NA | NA | NA | NA | NA | NA | NA | NA | NA |
| ROYAL ORTHOPAEDIC HOSPITAL NHS FOUNDATION TRUST | 1 | 1 | 1 | NA | NA | NA | NA | NA | NA | NA | NA | NA |
| ROYAL SURREY COUNTY HOSPITAL NHS FOUNDATION TRUST | 8 | 16 | 1 | 6 | 42 | 1 | 8 | 14 | 2 | 3 | 8 | 1 |
| ROYAL UNITED HOSPITAL BATH NHS TRUST | 16 | 26 | 2 | 6.5 | 47 | 1 | 14 | 30 | 5 | 9 | 15 | 1 |
| ROYAL WOLVERHAMPTON HOSPITALS NHS TRUST | 12 | 43 | 1 | 4 | 83 | 1 | 5 | 19 | 1 | 13.5 | 35 | 2 |
| SALFORD ROYAL NHS FOUNDATION TRUST | 15 | 45 | 1 | 11 | 49 | 1 | 24 | 58 | 5 | 26 | 57 | 2 |
| SALISBURY NHS FOUNDATION TRUST | 1 | 1 | 1 | 2 | 2 | 1 | NA | NA | NA | NA | NA | NA |
| SANDWELL AND WEST BIRMINGHAM HOSPITALS NHS TRUST | 6 | 75 | 1 | 9 | 50 | 1 | 20 | 32 | 1 | 13 | 57 | 4 |
| SHEFFIELD CHILDREN'S NHS FOUNDATION TRUST | 1 | 2 | 1 | 1 | 2 | 1 | 1 | 1 | 1 | 3 | 6 | 1 |
| SHEFFIELD TEACHING HOSPITALS NHS FOUNDATION TRUST | 1.5 | 2 | 1 | 3 | 15 | 1 | 1.5 | 6 | 1 | 1 | 3 | 1 |
| SHERWOOD FOREST HOSPITALS NHS FOUNDATION TRUST | 7.5 | 15 | 3 | 3 | 19 | 1 | 6.5 | 12 | 1 | 11 | 23 | 7 |
| SHREWSBURY AND TELFORD HOSPITAL NHS TRUST | 1 | 1 | 1 | 3 | 21 | 1 | 12 | 24 | 1 | 3 | 10 | 1 |
| SOMERSET NHS FOUNDATION TRUST | 19 | 27 | 2 | 6 | 82 | 1 | 23 | 30 | 2 | 1.5 | 4 | 1 |
| SOUTH DEVON HEALTHCARE NHS FOUNDATION TRUST | 4.5 | 8 | 1 | 6.5 | 15 | 1 | 10.5 | 23 | 3 | 2 | 5 | 1 |
| SOUTH TEES HOSPITALS NHS FOUNDATION TRUST | 16 | 25 | 1 | 2 | 14 | 1 | 2 | 10 | 1 | 1 | 1 | 1 |
| SOUTH TYNESIDE AND SUNDERLAND NHS FOUNDATION TRUST | 12 | 35 | 1 | 4.5 | 65 | 1 | 15 | 33 | 3 | 12 | 47 | 3 |
| SOUTH WARWICKSHIRE GENERAL HOSPITALS NHS TRUST | 15 | 22 | 2 | 7 | 41 | 1 | 5 | 14 | 1 | 3.5 | 8 | 1 |
| SOUTHAMPTON UNIVERSITY HOSPITALS NHS TRUST | 5 | 19 | 1 | 5 | 46 | 1 | 7 | 18 | 1 | 4 | 15 | 1 |
| SOUTHPORT AND ORMSKIRK HOSPITAL NHS TRUST | 1.5 | 2 | 1 | 2 | 6 | 1 | 1 | 3 | 1 | 1 | 1 | 1 |
| ST GEORGE'S HEALTHCARE NHS TRUST | 12 | 37 | 4 | 4 | 64 | 1 | 3 | 4 | 2 | NA | NA | NA |
| ST HELENS AND KNOWSLEY HOSPITALS NHS TRUST | 15.5 | 37 | 3 | 11.5 | 76 | 1 | 2 | 21 | 1 | 5 | 22 | 2 |
| STOCKPORT NHS FOUNDATION TRUST | 12 | 26 | 1 | 4 | 30 | 1 | 11 | 18 | 2 | 6 | 6 | 1 |
| SURREY AND SUSSEX HEALTHCARE NHS TRUST | 23 | 35 | 2 | 5 | 56 | 1 | 15 | 23 | 4 | 16.5 | 21 | 12 |
| TAMESIDE HOSPITAL NHS FOUNDATION TRUST | 8 | 24 | 1 | 7 | 27 | 1 | 5 | 17 | 1 | 2 | 6 | 1 |
| UNITED LINCOLNSHIRE HOSPITALS NHS TRUST | 45 | 138 | 4 | 13.5 | 78 | 1 | 32 | 52 | 24 | 29 | 49 | 20 |
| UNIVERSITY HOSPITAL OF NORTH STAFFORDSHIRE NHS TRUST | 60 | 140 | 7 | 5 | 111 | 1 | 23 | 34 | 6 | 18 | 37 | 1 |
| UNIVERSITY HOSPITALS BIRMINGHAM NHS FOUNDATION TRUST | 39 | 113 | 1 | 30 | 291 | 2 | 39 | 60 | 10 | 17.5 | 57 | 1 |
| UNIVERSITY HOSPITALS BRISTOL NHS FOUNDATION TRUST | 33.5 | 62 | 2 | 5 | 110 | 1 | NA | NA | NA | NA | NA | NA |
| UNIVERSITY HOSPITALS COVENTRY AND WARWICKSHIRE NHS TRUST | 40 | 62 | 9 | 9 | 146 | 1 | 26 | 35 | 14 | 19.5 | 49 | 1 |
| UNIVERSITY HOSPITALS OF LEICESTER NHS TRUST | 63 | 85 | 3 | 51 | 104 | 1 | NA | NA | NA | NA | NA | NA |
| UNIVERSITY HOSPITALS OF MORECAMBE BAY NHS FOUNDATION TRUST | 1 | 3 | 1 | 2 | 13 | 1 | 4.5 | 10 | 1 | 3 | 4 | 1 |
| WALSALL HEALTHCARE NHS TRUST | 7 | 36 | 1 | 2.5 | 15 | 1 | NA | NA | NA | NA | NA | NA |
| WALTON CENTRE NHS FOUNDATION TRUST | NA | NA | NA | 1 | 1 | 1 | NA | NA | NA | NA | NA | NA |
| WARRINGTON AND HALTON HOSPITALS NHS FOUNDATION TRUST | 24 | 36 | 7 | 10 | 79 | 1 | 7.5 | 21 | 4 | 5 | 12 | 5 |
| WEST HERTFORDSHIRE HOSPITALS NHS TRUST | 17 | 27 | 1 | 3 | 33 | 1 | 11 | 18 | 3 | 13 | 32 | 2 |
| WEST SUFFOLK HOSPITALS NHS TRUST | 7.5 | 28 | 1 | 4 | 60 | 1 | 22 | 29 | 13 | 9.5 | 30 | 1 |
| WESTERN SUSSEX HOSPITALS NHS FOUNDATION TRUST | 11 | 27 | 1 | 4 | 172 | 1 | 7 | 15 | 1 | 1.5 | 2 | 1 |
| WHITTINGTON HEALTH NHS TRUST | 7.5 | 27 | 1 | 4.5 | 21 | 1 | 11 | 22 | 4 | 14.5 | 31 | 2 |
| WIRRAL UNIVERSITY TEACHING HOSPITAL NHS FOUNDATION TRUST | 23 | 41 | 1 | 10 | 84 | 1 | 22 | 32 | 8 | 15.5 | 42 | 2 |
| WORCESTERSHIRE ACUTE HOSPITALS NHS TRUST | 9 | 19 | 7 | 6 | 45 | 1 | 10 | 18 | 2 | 7.5 | 18 | 2 |
| WRIGHTINGTON, WIGAN AND LEIGH NHS FOUNDATION TRUST | 43 | 80 | 12 | 7 | 34 | 1 | 11 | 21 | 5 | 12 | 38 | 2 |
| YEOVIL DISTRICT HOSPITAL NHS FOUNDATION TRUST | 10 | 19 | 2 | 3 | 34 | 1 | 7 | 18 | 3 | 6 | 10 | 1 |
| YORK HOSPITALS NHS FOUNDATION TRUST | 32 | 65 | 2 | 7 | 92 | 1 | 22 | 30 | 16 | 18 | 28 | 12 |

## Table S6. Predicted total number of nosocomial infections in the acute trusts studied.

These estimates do not include patients who were infected in the hospital but discharged before developing COVID-19 symptoms.

| Testing/reporting levels | Estimate | Lower 95% confidence interval | Upper 95% confidence interval |
| --- | --- | --- | --- |
| 200% of actual testing | 10,931 | 3496 | 18,377 |
| 175% of actual testing | 11,089 | 3564 | 18,623 |
| 150% of actual testing | 11,278 | 3646 | 18,926 |
| 125% of actual testing | 11,512 | 3740 | 19,314 |
| Actual testing | 11,816 | 3847 | 19,838 |
| 75% of actual testing | 12,236 | 3966 | 20,605 |
| 50% of actual testing | 12,891 | 4093 | 21,895 |
| 25% of actual testing | 14,207 | 4189 | 24,437 |

## Table S7. Sensitivity analysis.

**Logistic regression model for the prevalence of nosocomial COVID-19 infections, defined using a 14-day cut-off, among all COVID-19 infections in hospitalised patients.**

|  | **November–December 2020 (pre-vaccination) (1)** | **January–July 2021 (vaccine rollout) (2)** | **August–November 2021 (Delta) (3)** | **December 2021–March 2022 (Omicron) (4)** |
| --- | --- | --- | --- | --- |
| Number of observations^1^ | 1314 | 2852 | 1489 | 829 |
| LFD coverage (per 100% increase) | 0.99  (0.96, 1.01) | 0.92  (0.88, 0.97) | 1.00  (0.78, 1.29) | 0.74  (0.57, 0.98) |
| Trust LFD positivity (per 1/1000 increase) | 0.98  (0.97, 1.00) | 1.00  (0.99, 1.01) | 0.99  (0.96, 1.03) | 1.00  (0.99, 1.01) |
| Local COVID-19 prevalence (per 1/1000 increase) | 0.99  (0.96, 1.02) | 0.97  (0.92, 1.02) | 0.99  (0.92, 1.07) | 0.99  (0.97, 1.02) |
| Alpha variant prevalence (per 0.1 increase) | 1.02  (0.94, 1.11) | 0.98  (0.88, 1.09) | N/A | N/A |
| Delta variant prevalence (per 0.1 increase) | N/A | 1.10  (0.85, 1.41) | Delta variant prevalent | 1.23  (0.46, 3.33) |
| Omicron variant prevalence (per 0.1 increase) | N/A | N/A | N/A | 1.43  (0.56, 3.67) |
| Average income deprivation score^2^ per trust (per 0.01 unit increase) | 0.11  (0.05, 0.25) | 0.39  (0.17, 0.90) | N/A | N/A |

Odds ratios (95% CI) are shown.

^1^Number of trust/week observations included in the model.

^2^Average income deprivation score, median (range) 0.130 (0.053–0.239) for trusts included in the analysis. A higher score means greater deprivation; 95% CI are shown in brackets. Adjusted for trust and calendar week (as fixed effects) and the number of new COVID-19 cases identified at the trust that week.

## Table S8. Data inputs and assumptions for the testing service in healthcare

| **Parameter** | **Value** | **Source** |
| --- | --- | --- |
| Reduction in nosocomial infections | 16.8% (sensitivity analysis with range of 8–20 %) | Statistical analysis |
| Hospitalisation fatality ratio (HFR) | 20.13 | Calculated from ONS data (deaths/hospitalisations) [9] |
| QALYs for death | 6.78 (4.98–8.8) | [8] |
| QALYs for hospitalisations | 0.201 | [8] |
| QALYs for ICU admission | 0.15 | (Sandmann, Davies et al. 2021 |
| QALYs for symptomatic COVID-19 infections | 0.008 | [10] |
| Proportion of hospitalised patients with major manifestations | 0.41 (≥19 years)  0.2 (≤18 years) | Statistical analysis (healthcare) |
| Proportion of hospitalised patients with pneumonia | 0.42 (≥19 years)  0.11 (≤18 years) | Statistical analysis (healthcare) |
| Proportion of hospitalised patients with major manifestations or pneumonia in ICU | 0.11 (≥19 years)  0.9 (≤18 years) | Statistical analysis (healthcare) |
| Cost per hospitalisation (GBP) due to a COVID-19 infection | 2771 (≥19 years)  3138 (≤18 years) | NHS Schedule of Costs [11] |
| Cost per hospitalisation (GBP) due to a COVID-19 infection, with major manifestations | 4507 (≥19 years)  8606 (≤18 years) | NHS Schedule of Costs [11] |

## Table S9. Summary of the cost effectiveness of weekly and daily testing with respect to nosocomial infections averted during the evaluation period (October 2020 to March 2022).

|  | Weekly testing | | | Daily testing | | |  |
| --- | --- | --- | --- | --- | --- | --- | --- |
| Testing level | 8% | 16% | 20% | 25% | 15% | 30% | |
| Number of nosocomial infections averted | 17,500 | 35,000 | 43,800 | 50,400 | 32,800 | 65,700 | |
| Number of deaths averted due to nosocomial infections averted | 5,500 | 11,000 | 13,800 | 15,800 | 10,300 | 20,700 | |
| Cost per nosocomial infection averted (GBP) | 101,100 | 50,600 | 40,5500 | 35,200 | 53,900 | 27,000 | |
| Cost per death averted (GBP) | 320,800 | 160,000 | 127,900 | 410,800 | 630,300 | 314,800 | |
| Number of QALYs gained | 38,000 | 76,100 | 95,100 | 109,400 | 71,400 | 142,700 | |
| Cost per QALY gained (GBP) | 46,400 | 23,200 | 18,500 | 59,500 | 91,300 | 45,600 | |

The statistical analysis presented estimated that HCW testing was associated with a 16.8% (95% CI 8.2%, 18.8%) reduction in nosocomial infections compared with a testing scenario at 25% of actual levels.

# Supporting information – references

1. UK Health Security Agency. NHS Test and Trace statistics (England): methodology 2022 [26 March 2024]. Available from: <https://www.gov.uk/government/publications/nhs-test-and-trace-statistics-england-methodology/nhs-test-and-trace-statistics-england-methodology>.
2. Meakin S, Abbott S, Choi H, Funk S. NHS trust level Covid-19 data aggregated to a range of spatial scales 2020 [2 May 2024]. Available from: https://epiforecasts.io/covid19.nhs.data/index.html.
3. Riley S, Atchison C, Ashby D, Donnelly CA, Barclay W, Cooke GS, et al. REal-time Assessment of Community Transmission (REACT) of SARS-CoV-2 virus: Study protocol. Wellcome Open Res. 2020;5:200. Epub 2021/05/19. doi: 10.12688/wellcomeopenres.16228.2. PubMed PMID: 33997297; PubMed Central PMCID: PMCPMC8095190.
4. Nicholson G, Lehmann B, Padellini T, Pouwels KB, Jersakova R, Lomax J, et al. Improving local prevalence estimates of SARS-CoV-2 infections using a causal debiasing framework. Nature Microbiology. 2022;7(1):97-107. doi: 10.1038/s41564-021-01029-0.
5. Bajaj S, Chen S, Creswell R, Naidoo R, Tsui JL, Kolade O, Nicholson G, Lehmann B, Hay JA, Kraemer MU, Aguas R. COVID-19 testing and reporting behaviours in England across different sociodemographic groups: a population-based study using testing data and data from community prevalence surveillance surveys. The Lancet Digital Health. 2024 Nov 1;6(11):e778-90.
6. Chen S, Creswell R, Hounsell R, Cantrell L, Bajaj S, Dahal P, Hei JT, Kolade O, Amswych MA, Naidoo R, Fowler T. Mass testing for discovery and control of COVID-19 outbreaks in adult social care: an observational study and cost-effectiveness analysis of 14 805 care homes in England. BMJ Public Health. 2025 Mar 28;3(1).
7. Office for National Statistics. Mapping income deprivation at a local authority level 2021 [6 May 2024]. Available from: <https://www.ons.gov.uk/peoplepopulationandcommunity/personalandhouseholdfinances/incomeandwealth/articles/mappingincomedeprivationatalocalauthoritylevel/2021-05-24>.
8. Office for National Statistics. Deaths registered weekly in England and Wales, provisional Statistical bulletins [16 June 2025]. Available from <https://www.ons.gov.uk/peoplepopulationandcommunity/birthsdeathsandmarriages/deaths/bulletins/deathsregisteredweeklyinenglandandwalesprovisional/previousreleases?page=22>
9. Office for National Statistics. Deaths registered weekly in England and Wales provisional: week ending 16 April 2021 [16 June 2025]. Available from <https://www.ons.gov.uk/peoplepopulationandcommunity/birthsdeathsandmarriages/deaths/bulletins/deathsregisteredweeklyinenglandandwalesprovisional/weekending16april2021>
10. Office for National Statistics. Coronavirus (COVID-19) latest insights: Hospitals [16 June 2025]. Available from <https://www.ons.gov.uk/peoplepopulationandcommunity/healthandsocialcare/conditionsanddiseases/articles/coronaviruscovid19latestinsights/hospitals>
11. GOV.UK. UKHSA data dashboard [16 June 2025]. Available from <https://ukhsa-dashboard.data.gov.uk>
12. GOV.UK. National flu and COVID-19 surveillance reports: 2022 to 2023 season. Available from <https://www.gov.uk/government/statistics/national-flu-and-covid-19-surveillance-reports-2022-to-2023-season>
13. Grant RL. Converting an odds ratio to a range of plausible relative risks for better communication of research findings. Bmj. 2014;348:f7450. Epub 2014/01/28. doi: 10.1136/bmj.f7450. PubMed PMID: 24464277.
14. Office for National Statistics. Coronavirus (COVID-19) [6 May 2024]. Available from: <https://www.ons.gov.uk/peoplepopulationandcommunity/healthandsocialcare/conditionsanddiseases>.
15. Sandmann FG, Davies NG, Vassall A, Edmunds WJ, Jit M. The potential health and economic value of SARS-CoV-2 vaccination alongside physical distancing in the UK: a transmission model-based future scenario analysis and economic evaluation. Lancet Infect Dis. 2021;21(7):962-74. Epub 2021/03/22. doi: 10.1016/s1473-3099(21)00079-7. PubMed PMID: 33743846; PubMed Central PMCID: PMCPMC7972313.
16. NHS England and NHS Improvement. Novel coronavirus (COVID-19) standard operating procedure — NHS England and NHS Improvement rollout of lateral flow devices for asymptomatic staff testing for SARS-CoV-2 (phase 2: trusts). 2020 18 October 2022]. Available from: [https://www.england.nhs.uk/coronavirus/wp-content/uploads/sites/52/2020/11/C0873_i_SOP_LFD-rollout-forasymptomatic staff-testing_phase-2-trusts-v1.1_16-nov20.pdf](https://www.england.nhs.uk/coronavirus/wp-content/uploads/sites/52/2020/11/C0873_i_SOP_LFD-rollout-forasymptomatic%20staff-testing_phase-2-trusts-v1.1_16-nov20.pdf).
17. UK Health Security Agency (confidential internal document), Supporting information for future evaluations of effectiveness of national testing services in England. 2022.
18. NHS England. NHS asymptomatic staff testing: Lateral flow device distribution and assuring compliance to testing regimes (letter). 2021 [18 October 2022]. Available from: <https://www.england.nhs.uk/coronavirus/wpcontent/uploads/sites/52/2020/12/C1276_Staff-testing-letter_29june21.pdf>.
19. NHS England and NHS Improvement. Novel coronavirus (COVID-19) standard operating procedure — Use of lateral flow devices for asymptomatic staff testing for SARS CoV-2 in all NHS Staff. 2021 17 October 2022]; Available from: <https://www.england.nhs.uk/coronavirus/wpcontent/uploads/sites/52/2020/11/C1385_Use-of-lateral-flow-devices-for-SARS-CoV-2-in-all-NHS-staff-standard-operating-procedure_September_v2.pdf>.
20. Department of Health and Social Care. Daily rapid testing for COVID-19 contacts launches this week. 2021 21 January 2023]. Available from: <https://www.gov.uk/government/news/daily-rapid-testing-forcovid-19-contacts-launches-this-week>.
21. Bow, S.M.A., et al., An evaluation of a pilot of daily testing of SARS-CoV-2 contacts in acute hospital and ambulance trusts in England. Public Health, 2022. 209: p. 46-51.
22. NHS England. NHS asymptomatic staff testing: Lateral flow device distribution and assuring compliance to testing regimes (letter). 2021 18 October 2022]; Available from: <https://www.england.nhs.uk/coronavirus/wpcontent/uploads/sites/52/2020/12/C1276_Staff-testing-letter_29june21.pdf>.
23. Carr, J. DfE doubles down on policy that in-school Covid test positives don’t need confirmatory PCR. 2021 [2 January 2023]; Available from: <https://schoolsweek.co.uk/dfe-doubles-down-on-policy-thatin-school-covid-test-positives-dont-need-confirmatorypcr/>.
24. NHS Digital. NHS workforce statistics [9 May 2024]. Available from: <https://digital.nhs.uk/data-and-information/publications/statistical/nhs-workforce-statistics>.
